# The Current Status of Telemedicine Technology Use Across the World Health Organization European Region: An Overview of Systematic Reviews

**DOI:** 10.2196/40877

**Published:** 2022-10-27

**Authors:** Francesc Saigí-Rubió, Israel Júnior Borges do Nascimento, Noemí Robles, Keti Ivanovska, Che Katz, Natasha Azzopardi-Muscat, David Novillo Ortiz

**Affiliations:** 1 Faculty of Health Sciences Universitat Oberta de Catalunya Barcelona Spain; 2 Research Group in Epidemiology and Public Health in the Digital Health context Universitat Oberta de Catalunya Barcelona Spain; 3 School of Medicine and University Hospital Federal University of Minas Gerais Belo Horizonte Brazil; 4 School of Medicine Medical College of Wisconsin Milwaukee, WI United States; 5 Division of Country Health Policies and Systems World Health Organization, Regional Office for Europe Copenhagen Denmark

**Keywords:** telemedicine, Europe, World Health Organization, mobile phone

## Abstract

**Background:**

Several systematic reviews evaluating the use of telemedicine by clinicians, patients, and health authorities to improve the delivery of care in the 53 member states of the World Health Organization (WHO) European Region have been conducted in recent years. However, a study summarizing the findings of these reviews has not been conducted.

**Objective:**

This overview of systematic reviews aimed to summarize findings regarding the use of telemedicine across the 53 member states and identify the medical fields and levels of care in and at which the effectiveness, feasibility, and applicability of telemedicine have been demonstrated. The barriers to and facilitators of telemedicine use were also evaluated and collated to help with the design and implementation of telemedicine interventions.

**Methods:**

Through a comprehensive systematic evaluation of the published and unpublished literature, we extracted clinical, epidemiological, and technology-related data from each review included in the study. We focused on evaluating the barriers to and facilitators of the use of telemedicine apps across the 53 member states considered. We rated the methodological quality of each of the included reviews based on A Measurement Tool to Assess Systematic Review 2 approach and judged the overall certainty of evidence by using the Grading of Recommendations, Assessment, Development, and Evaluations methodology. The entire process was performed by 2 independent authors.

**Results:**

This overview drew on data from >2239 primary studies, with >20,000 enrolled patients in total, within the WHO European Region. On the basis of data from randomized trials, observational studies, and economic evaluations from several countries, the results show a clear benefit of telemedicine technologies in the screening, diagnosis, management, treatment, and long-term follow-up of a series of chronic diseases. However, we were unable to pool the results into a reliable numeric parameter because of the high heterogeneity of intervention methodologies, scheduling, primary study design discrepancies, settings, and geographical locations. In addition to the clinical outcomes of the interventions, the social and economic outcomes are highlighted.

**Conclusions:**

The application of telemedicine is well established across countries in the WHO European Region; however, some countries could still benefit from the many uses of these digital solutions. Barriers related to users, technology, and infrastructure were the largest. Conversely, the provision of health services using technological devices was found to significantly enhance patients’ clinical outcomes, improve the long-term follow-up of patients by medical professionals, and offer logistical benefits for both patients and health workers.

**Trial Registration:**

PROSPERO (International Prospective Register of Systematic Reviews) CRD42022309375; https://www.crd.york.ac.uk/prospero/display_record.php?RecordID=309375

## Introduction

Telemedicine is an accessible, cost-effective medical system, delivering high-quality care and reducing overall morbidity and mortality [1,2]. Telecommunications have benefited patient-related outcomes, improved health workers’ performance, reduced health workers’ workload, and decreased the isolation of health care professionals in remote locations [3,4]. Remote clinical care has increased, particularly during the COVID-19 pandemic [5-7]. The pandemic decreased in-person outpatient consultations and consequently increased telehealth legislation and public health guidance, which indirectly contributed to decline in transmissibility and mortality rates [8-10].

In the World Health Organization (WHO) European Region, an extensive body of literature has recently been produced, evidencing multiple positive health-related outcomes and the creation of integrated and finely structured remote health counseling programs [11-15]. European Union countries are covered by the European Commission’s digital policies and priorities, which provide a common framework for digital interventions [16]. In addition, the European Commission provides funding programs to develop and implement these guidelines. No study has collated and summarized the available evidence to indicate the status of telemedicine in Europe. Therefore, this overview of systematic reviews aims to summarize findings regarding the use of telemedicine across the 53 member states of the WHO European Region and to identify the medical fields and levels of care in and at which the effectiveness, feasibility, and applicability of telemedicine have been demonstrated. The barriers to and facilitators of telemedicine use were also evaluated and collated to help with the design and implementation of telemedicine interventions.

## Methods

### Overview

The protocol for this overview of systematic reviews was published on February 17, 2022, in PROSPERO (International Prospective Register of Systematic Reviews; CRD42022309375; Multimedia Appendix 1). There were no substantial deviations from the proposed methodology. We adhered to an adapted version of the guidelines of the Cochrane Handbook for Systematic Reviews of Interventions and followed the PRISMA (Preferred Reporting Items for Systematic Reviews and Meta-Analyses) statement and the Preferred Reporting Items for Overviews of Systematic Reviews checklist [17-19].

### Ethical Considerations

This study relied on secondary data; therefore, no ethics approval or patient consent was required.

### Search Strategy

Five databases (PubMed, Embase, Web of Science, the Cochrane Library, and Scopus) were searched from their inception to February 14, 2022. The search strategy is presented in Multimedia Appendix 2. All studies were processed in EndNote X9 (Clarivate) and subsequently imported into Covidence. In addition, we manually searched the first 2 pages of Google Scholar results and reviewed shortlisted records to identify additional studies. If a full-text study could not be obtained, a ResearchGate request was sent to gain full access.

### Selection Criteria

Two investigators independently assessed titles and abstracts and analyzed appropriate studies through full-text evaluation. Cochrane and non-Cochrane systematic reviews with or without meta-analyses were included if they had adequately displayed the status of telemedicine among the 53 member states of the WHO European Region or reported on the barriers to and facilitators of the use of such technologies, regardless of publication data and the primary language. Reviews were considered eligible if >50% of the primary studies originated from the WHO European Region [20]. As telemedicine solutions and the publication time for manuscripts increased during the pandemic, we considered preprints and unpublished data. Exclusion criteria were (1) study unrelated to telemedicine, (2) study full text unavailable on the web, and (3) the scope of interventions does not include the WHO European Region. Any disagreement was resolved by discussion a third reviewer. We classified selected studies into systematic reviews (with or without meta-analyses), scoping reviews, and “others” (studies that were not classified into either of the 2 previously mentioned designs yet used a comprehensive execution methodology).

### Data Extraction and Management

Two investigators independently extracted data by using Excel (Microsoft). A third party resolved discrepancies. The data extraction form (Multimedia Appendix 3) contains review identification features, telemedicine specialty, medical specialty or disease focus, countries and settings of focus, sample size, the main findings, barriers, facilitators, and the main challenges associated with the use of telemedicine.

### Assessment of Methodological Quality

Two investigators independently appraised methodological quality by using A Measurement Tool to Assess Systematic Review 2 (AMSTAR 2). Discrepancies were resolved through consensus. In addition to the systematic reviews of intervention trials, additional types of literature were included. Some AMSTAR 2 ratings were therefore adjusted (Multimedia Appendix 4 [21-53]). After rating each domain, overall confidence in the results of the review were judged as “critically low,” “low,” “moderate,” or “high.” Adherence ratings for the transparency of the researchers’ judgments were reported, with explanations for each item.

### Data Synthesis and Evaluation of the Level of Evidence

Evidence was synthesized based on the core disease or condition by using the *International Classification of Diseases*, 10th edition (ICD-10). A comprehensive narrative description of the characteristics and main findings was created and displayed in summarization tables. Furthermore, significant barriers and facilitators were presented, categorized, and discussed using the tree-mapping method, which displays hierarchical data as a set of nested rectangles. Limitations were also evaluated, and the effect of publication and small-study biases on results was considered. Finally, using an adapted version of the Grading of Recommendations, Assessment, Development, and Evaluations methodology, the evidence was assessed considering 5 modifiers: risk of bias in studies, inconsistency, imprecision, indirectness, and publication bias [54].

## Results

### Overview

**Figure 1:**
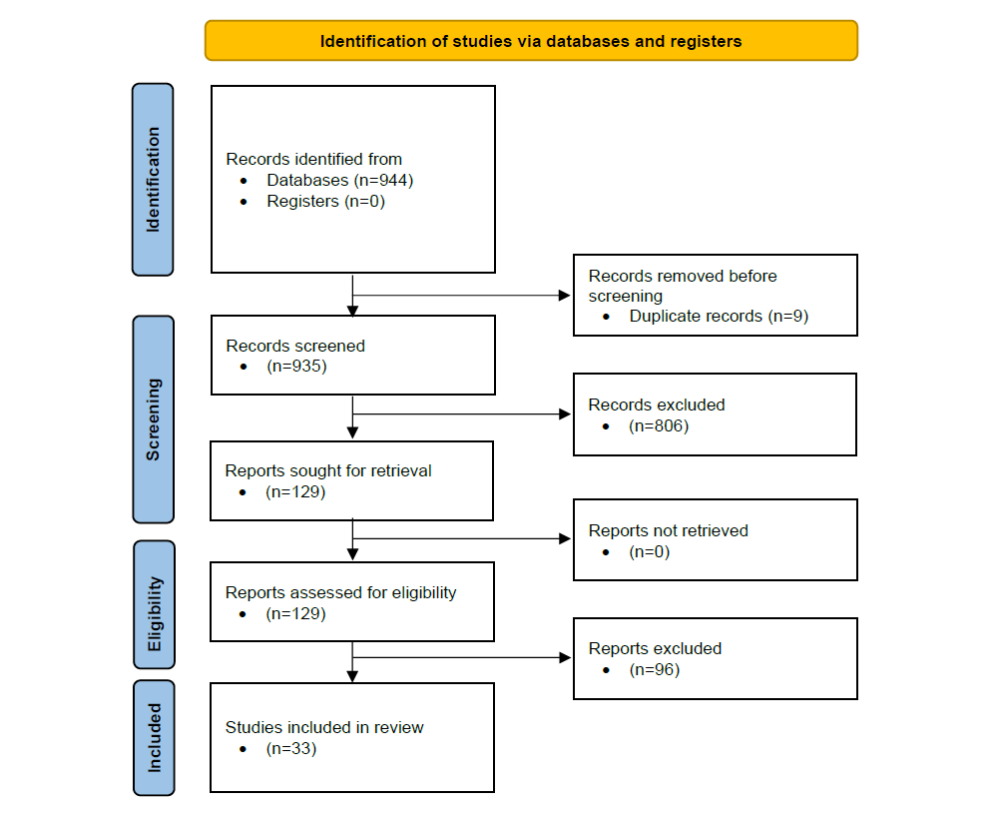
PRISMA (Preferred Reporting Items for Systematic Reviews and Meta-Analyses) 2020 flow diagram for new systematic reviews that include searches of databases and registers only.

In total, 944 records were retrieved, including 9 duplicates. In title and abstract screening, 806 publications were excluded. Of the remaining studies, 96 were excluded. Therefore, 33 articles were included in the final analysis. Additional records were found after checking the reference lists of included reviews. The overview flowchart is shown in Figure 1.

### Characteristics of the Included Reviews

The characteristics of the included reviews are reported in in Multimedia Appendix 5 [21-53]. A total of 33 reviews were published between 2011 and 2022, mostly (17/33, 51%) between 2020 and 2021. The reviews were published in a range of indexed journals in English, Portuguese, or German. Translation support was sought through Cochrane TaskExchange. Regarding primary studies, 23 out of 53 member states of the European Region had at least one study evaluating the status of telemedicine (the United Kingdom, 17/33, 52%; Italy, 15/33, 45%; Denmark, 13/33, 39%; the Netherlands, 13/33, 39%; Germany, 8/33, 24%; Norway, 8/33, 24%; Belgium, 6/33, 18%; Austria, Finland, France, and Sweden, 5/33, 15%; Spain, 4/33, 12%; Greece, 3/33, 9%; Iceland, Poland, Switzerland, and Türkiye, 2/33, 6%; and Albania, Ireland, Ukraine, Romania, and the Russian Federation, 1/33, 3%). The included reviews focused on various conditions or diseases, mainly those related to mental and behavioral disorders (Chapter V of ICD-10; 4/33, 12%), diseases of the circulatory system (Chapter IX of ICD-10; 4/33, 12%), diseases of the respiratory system (Chapter X of ICD-10; 4/33, 12%), diseases of the nervous system (Chapter VI of ICD-10; 3/33, 9%), and diseases of the skin and subcutaneous tissue (Chapter XII of ICD-10; 3/33, 9%). Of the 33 studies, 12 (33%) were classified as “multifocal studies” because they assessed multiple conditions or diseases and could not be assigned to just 1 chapter of the ICD-10.

### Population and Study Designs

A total of 2239 primary studies were characterized as observational, interventional, medical, and economic modeling-based analyses and mixed methods studies. Publication designs were mostly “systematic reviews without meta-analyses” (19/33, 58%), “scoping reviews” (8/33, 24%), “others” (3/33, 9%), and “systematic reviews with meta-analyses” (3/33, 9%). Not all reviews specified the number of patients, but the data suggest that there were 61,589 patients.

### Quality and Certainty of Evidence in Individual Systematic Reviews

The results of the AMSTAR 2 assessment showed that the main methodological weaknesses were a lack of protocol registration, no evaluation of the overall risk of bias by using validated approaches, a lack of disclosure and justification of excluded studies, and the absence of detailed reporting of the critical characteristics of the included reviews (Table 1). In summary, 88% (29/33) of the systematic reviews were judged to deliver “critically low” quality evidence and 12% (4/33) “low” quality evidence. None of the reviews produced high- or moderate-quality evidence. Therefore, confidence in the overall tendency of the effect was limited (Multimedia Appendix 6).

**Table 1:** Reliability of included reviews based on A Measurement Tool to Assess Systematic Review (AMSTAR 2) judgments^a^.

Review ID (reference)	1^b^	2^c^	3^d^	4^e^	5^f^	6^g^	7^h^	8^i^	9^j^	10^k^	11^l^	12^m^	13^n^	14^o^	15^p^	16^q^	Overall quality
Allner et al [21]	Y^r^	PY^s^	N^t^	PY	Y	Y	N	N	N	N	NMAC^u^	NMAC	N	N	NMAC	Y	Very Low^v^
Brunetti al [22]	Y	N	Y	PY	Y	Y	N	N	Y	N	Y	N	N	N	Y	Y	Very Low^v^
Carbo et al [23]	Y	PY	Y	N	Y	Y	N	PY	Y	Y	Y	N	N	Y	N	Y	Very Low^v^
Cordes et al [24]	Y	N	Y	PY	N	N	N	PY	Y	N	NMAC	NMAC	N	N	NMAC	Y	Very Low^v^
Cruz et al [25]	Y	N	Y	PY	Y	Y	N	Y	N	N	NMAC	NMAC	N	N	NMAC	Y	Low^w^
Elbaz et al [26]	Y	N	Y	PY	Y	N	N	PY	N	N	NMAC	NMAC	N	N	NMAC	Y	Very Low^v^
Farabi et al [27]	Y	N	Y	PY	Y	Y	N	PY	Y	N	NMAC	NMAC	Y	N	NMAC	Y	Very Low^v^
Gaveikaite et al [28]	Y	N	Y	PY	Y	Y	N	Y	Y	N	NMAC	NMAC	N	Y	NMAC	Y	Very Low^v^
Glinkowski et al [29]	Y	N	Y	PY	Y	Y	N	N	N	N	NMAC	NMAC	N	N	NMAC	Y	Very Low^v^
Hallensleben et al [30]	Y	N	Y	PY	Y	Y	N	Y	N	N	NMAC	NMAC	N	N	NMAC	Y	Very Low^v^
Hartasanchez et al [31]	Y	N	Y	PY	Y	Y	N	PY	N	N	NMAC	NMAC	N	N	NMAC	Y	Very Low^v^
Hrynyschyn et al [32]	Y	N	Y	PY	Y	Y	N	Y	Y	N	NMAC	NMAC	Y	N	NMAC	Y	Very Low^v^
Karamanidou et al [33]	Y	PY	Y	PY	Y	Y	N	PY	N	Y	NMAC	NMAC	N	Y	NMAC	Y	Very Low^v^
Kierkegaard et al [34]	Y	PY	Y	PY	Y	N	N	N	N	N	NMAC	NMAC	N	Y	NMAC	Y	Very Low^v^
Kingsdorf et al [35]	Y	PY	Y	PY	Y	Y	N	PY	N	N	NMAC	NMAC	N	Y	NMAC	Y	Very Low^v^
Labiris et al [36]	Y	N	Y	PY	N	N	N	PY	N	N	NMAC	NMAC	N	N	NMAC	Y	Very Low^v^
Maresca et al [37]	Y	PY	Y	N	N	N	N	PY	N	N	NMAC	NMAC	N	N	NMAC	N	Very Low^v^
Martin et al [38]	Y	PY	Y	N	Y	Y	N	Y	N	N	NMAC	NMAC	N	Y	NMAC	Y	Very Low^v^
McFarland et al [39]	Y	Y	Y	PY	Y	Y	N	Y	PY	N	Y	Y	Y	Y	Y	Y	Low^w^
Mold et al [40]	Y	PY	N	PY	Y	Y	Y	Y	N	N	NMAC	NMAC	N	Y	NMAC	Y	Very Low^v^
Nielsen et al [41]	Y	PY	Y	PY	N	N	N	PY	N	N	NMAC	NMAC	N	Y	NMAC	Y	Very Low^v^
O’Cathail et al [42]	Y	PY	Y	PY	Y	Y	N	PY	N	N	NMAC	NMAC	N	Y	NMAC	Y	Very Low^v^
Ohannessian et al [43]	Y	N	Y	PY	Y	Y	N	PY	N	N	NMAC	NMAC	N	N	NMAC	Y	Very Low^v^
Pron et al [44]	Y	PY	Y	PY	N	N	N	PY	PY	N	NMAC	NMAC	N	Y	NMAC	Y	Very Low^v^
Raja et al [45]	Y	N	Y	PY	Y	Y	N	PY	N	N	NMAC	NMAC	N	N	NMAC	Y	Very Low^v^
Simmonds-Buckley et al [46]	Y	PY	Y	Y	Y	Y	N	Y	PY	N	Y	Y	Y	Y	Y	Y	Low^w^
Singh et al [47]	Y	N	Y	PY	N	N	N	Y	N	N	NMAC	NMAC	N	N	NMAC	Y	Very Low^v^
Tokgoz et al [48]	Y	Y	Y	PY	Y	Y	N	PY	Y	Y	NMAC	NMAC	Y	Y	NMAC	Y	Low^w^
Trettel et al [49]	Y	N	N	PY	N	N	N	N	N	N	NMAC	NMAC	N	N	NMAC	Y	Very Low^v^
Udsen et al [50]	Y	PY	Y	PY	Y	Y	N	Y	N	N	NMAC	NMAC	N	Y	NMAC	N	Very Low^v^
Verma et al [51]	Y	N	Y	PY	Y	Y	Y	N	N	Y	NMAC	NMAC	N	N	NMAC	Y	Very Low^v^
Willard et al [52]	N	PY	N	PY	Y	Y	N	N	N	N	NMAC	NMAC	N	N	NMAC	Y	Very Low^v^
Zanin et al [53]	Y	PY	Y	PY	Y	Y	N	PY	PY	N	NMAC	NMAC	N	Y	NMAC	Y	Very Low^v^

^a^Judgments were made by 2 overview authors based on AMSTAR 2, a critical appraisal tool for systematic reviews that include randomized or nonrandomized studies of health care interventions or both.

^b^Domain 1—Did the research questions and inclusion criteria for the review include the components of PICO (Patients, Intervention, Comparator, and Outcomes)

^c^Domain 2—Did the report of the review contain an explicit statement that the review methods were established before the conduct of the review and did the report justify any significant deviations from the protocol?

^d^Domain 3—Did the review authors explain their selection of the study designs for inclusion in the review?

^e^Domain 4—Did the review authors use a comprehensive literature search strategy?

^f^Domain 5—Did the review authors perform study selection in duplicate?

^g^Domain 6—Did the review authors perform data extraction in duplicate?

^h^Domain 7—Did the review authors provide a list of excluded studies and justify the exclusions?

^i^Domain 8—Did the review authors describe the included studies in adequate detail?

^j^Domain 9—Did the review authors use a satisfactory technique for assessing the risk of bias (RoB) in individual studies that were included in the review?

^k^Domain 10—Did the review authors report on the sources of funding for the studies included in the review?

^l^Domain 11—If meta-analysis was performed, did the review authors use appropriate methods for statistical combination of results?

^m^Domain 12—If meta-analysis was performed, did the review authors assess the potential impact of RoB in individual studies on the results of the meta-analysis or other evidence synthesis?

^n^Domain 13—Did the review authors account for RoB in individual studies when interpreting or discussing the results of the review?

^o^Domain 14—Did the review authors provide a satisfactory explanation for, and discussion of, any heterogeneity observed in the results of the review?

^p^Domain 15—If they performed quantitative synthesis, did the review authors carry out an adequate investigation of publication bias (small-study bias) and discuss its likely impact on the results of the review?

^q^Domain 16—Did the review authors report any potential sources of conflict of interest, including any funding they received for conducting the review?

^r^Y: methodological requirements met.

^s^PY: methodological requirements partly met.

^t^N: methodological requirements not met.

^u^NMAC: no meta-analysis conducted.

^v^XX: studies rated as “critically low.”

^w^X: studies rated as “low.”

### Systematic Review Findings

The identified interventions were mainly telephone- and videoconferencing-based methodologies, although they also included mobile apps and exchanges of medical test results (Multimedia Appendix 7 [21-53]). Most studies focused on the effectiveness of telemedicine interventions. The studies demonstrated that, as telemedicine was effective in reducing time to access treatment (1/33, 3%; Chapter IX of ICD-10), time for clinical decisions (1/33, 3%; Chapter IX of ICD-10), unnecessary repeated examinations (1/33, 3%; Chapter VII of ICD-10), length of stay in hospital (1/33, 3%; Chapter IX of ICD-10), number of emergency visits (1/33, 3%; Chapter X of ICD-10), and the number of false positives (1/33, 3%; Chapter VII of ICD-10) and also provided more accurate diagnoses (2/33, 6%; Chapter XII of ICD-10), it improved some clinical outcomes such as anxiety and depression in mental health disorders (2/33, 6%; Chapter V of ICD-10), neurological symptoms (1/33, 3%; Chapter VI of ICD-10), , exacerbation rates in patients with chronic obstructive pulmonary disease (1/33, 3%; Chapter X of ICD-10), wound healing time in some skin diseases (1/33, 3%; Chapter XII of ICD-10), and aphasia symptoms (1/33, 3%; Chapter XVIII of ICD-10). Moreover, telemedicine was reliable and sensitive for detecting changes in cognition over time (1/33, 3%; Chapter V of ICD-10) and improving patients’ quality of life (2/33, 6%; Chapter X of ICD-10; 1/33, 3%; Chapter VI of ICD-10; and 1/33, 3%; Chapter XXI of ICD-10) and quality-adjusted life years (1/33, 3%; Chapter IX of ICD-10).

Nonsignificant or inconclusive effects were found for other outcomes, such as mortality rates in circulatory and skin diseases (2/33, 6%; Chapter IX of ICD-10; 1/33, 3%; Chapter XII of ICD-10), the number of excisions in skin diseases (1/33, 3%; Chapter XII of ICD-10), and the number of hospital admissions (1/33, 3%; Chapter IX of ICD-10; 2/33, 6%; Chapter X of ICD-10).

A total of 5 studies evaluated the usability and acceptance of telemedicine by medical personnel and patients with multiple morbidities, as well as their satisfaction with it. High acceptability was primarily because of cost reduction compared with standard care, convenience, improved follow-up, adherence to planned treatment, and time-saving. A study reported telehealth’s cost-effectiveness, finding no statistically significant difference between standard care and telehealth care.

### Barriers to and Facilitators of the Use of Telemedicine Interventions

The barriers, facilitators, and main challenges associated with the use of telemedicine are reported in Multimedia Appendix 8 [21-53]. Barriers and facilitators were grouped into the following domains: individual; organizational; clinical; economic; technological; and ethics, security, and privacy issues. Most barriers were in the individual domain, followed by technological; organizational; clinical; and ethics, security, and privacy issues domains, and finally the economic domain. Most facilitators were in the individual domain, followed by organizational and clinical domains, and then technological; economic; and ethics, security, and privacy issues domains (Table 2).

**Table 2:** List of barriers to and facilitators of the implementation of telemedicine across the 53 member states of the World Health Organization European Region and the main methodological limitations of the included studies.

Domain	Barriers	Facilitators
Individual domain	Shortcomings in technology-related knowledge and skill [21,24,41,45,51] Resistance to change [40,42,49,51] Patients’ age [24,40,41,45] Lack of motivation or support [36,45,51] Lack of confidence [49,51] Challenges for individuals with disabilities [26,51] Patients’ preference for face-to-face consultations [40,41,51] Low satisfaction [21,51] Language barriers [41,51] Lack of acceptance [52] Lack of usefulness [41] Less personal contact through telemedicine [39] Invasiveness [39] High attrition rate [35]	Patient empowerment [31,35,51] Participatory design [33,35,41,42] Motivation and engagement [31,36,42,51] Convenience [40,51,53] Patients’ age [24,41,45] Trust in technology [35,45] Patients feel safe and empowered to discuss personal issues [51] Physicians’ training and skills [31,45] Satisfaction [36,51] Adoption of digital culture [52] Patients sharing their experiences [45]
Organizational domain	The lack of integration into clinicians’ workflows [31,42,44,51] Socioeconomic aspects (financial limitations) [24,25,31,40] Lack of access to a helpful caregiver [26,39,45] Sociocultural aspects [21,40] Increase in workload [51] Scheduling conflicts [31] Lack of governance [52] No appropriate Health Information Systems framework [49] Organizational issues creating barriers to long-term implementation [26]	Reduction in response time [40,51,53] Integration into clinicians’ workflows [31,42] Decrease in workload [37,51] Access to a helpful caregiver and insights into patient’s home environment [45,51] Pandemic- created acceptance of technology [51] Increased adherence [51] Coordination between healthcare levels [52] Telemedicine champions [34]
Clinical domain	Limited scientific evidence [29,30,32,33,35] Patient recruitment barriers and low rates of patient participation [28,46] Difficulty in making clinical decisions [49,51] Changes to consultation protocols [51] Insufficient consultation time [51] Loss of physical and visual assessment of symptoms [51]	Clinical and professional benefits [33,34,51] Assessment after a specified period with service evaluations, including feedback from key stakeholders [33,42] Multidisciplinary care team interventions [33] The establishment of guidelines [49] Reduction in the number of visits [36] Frequent and multimodal communication between the health care professional and patient [38] Greater safety and efficacy [42] Better monitoring of cases [51]
Economic domain	Elevated cost of implementation [23,27,45] Lack of funding model [34,42,43,51] Scarce economic benefits [21]	Financial framework [49,51] Financial benefits [34,45] Cost savings [36]
Technological domain	Issues with internet access [24,26,31,35] Technology needs further development [23,37,45] Usability factors [25,31,41] Issues with information technology and systems infrastructure [25,42] Concerns about the reliability of the technology [21,41,45] Issues surrounding infrastructure [44] Conflicts of interoperability [52] Difficulties in implementation and follow-up over a longer period [27] Difficulties in readability [25] Limited accessibility to electronic devices [24]	Usability and user satisfaction factors [31,33,45] Internet availability [31,35] Possibilities of technology development [52] Accessibility support [31,35] Adaptable and self-configurable [52]
Ethics, security, and privacy issues	Private data security concerns or issues [38,45,51] Regulatory concerns or issues [21,44,49] Concerns about patient and staff safety [21,41] Ethical aspects [21]	Legal framework [49]

## Discussion

### Principal Findings

This overview of systematic reviews shows a substantial and unprecedented collection of findings, as it included relevant data from >2239 primary studies, with >20,000 enrolled patients in total, within the WHO European Region. On the basis of data from observational studies, randomized trials, and economic evaluations from several European countries, the results showed a clear benefit of telemedicine interventions in the screening, diagnosis, management, treatment, and long-term follow-up of a range of clinically and epidemiologically significant diseases.

The telemedicine technological solutions addressed have proven to be valid, reliable, and accurate in providing faster access to expert advice, decreasing the number of unnecessary specialist referrals and in-office consultations, as well as increasing patient satisfaction experience. In a comprehensive literature review of studies from the United States, Canada, Brazil, and Australia, Liddy et al [55] reported an increasing number of medical specialties adopting innovative health solutions in daily practice. This overview of systematic reviews has highlighted the scientific priority of research in the evaluation of disease-related clinical, economic, and social outcomes, focusing on medical conditions considered chronic diseases, such as mental, cardiovascular, and respiratory diseases [56].

Most studies were concentrated in European countries (such as Germany, Italy, Spain, and the United Kingdom), while Eastern Europe (such as Albania, Croatia, and Ukraine) was not evaluated in any study. Countries developing digital health implementation must consider leadership, governance, strategy, investment, infrastructure, legislation, policy, compliance, workforce, services, and apps in their digital health strategies. The 2015 WHO Global Survey on eHealth [57] revealed that 38% of the member states had not developed national telehealth policies or strategies, and 49% did not have mHealth programs [58,59].

Several studies reported barriers and facilitators that should be considered when planning and implementing telemedicine interventions. The individual domain was found to be the most influential in the use of telemedicine interventions, giving place to a greater number of barriers and facilitators. Shortcoming in technology-related knowledge and skills was the main challenge cited, followed by health care professionals’ resistance to procedural change [60-62]. According to some studies, the lack of technological applications integrated into clinicians’ practice exacerbated this, resulting in scheduling conflicts and affecting the quality of delivery. The presence of clinician champions working alongside other health care professionals might promote service adoption [63,64].

In this overview, health care professionals had heavy workloads that seemed to influence resistance by overshadowing benefits [61,65]. Conversely, integrating telemedicine into clinicians’ workflows, establishing guidelines, and increasing coordination among the levels of care were organizational changes required for the proper adoption of telemedicine [66]. In addition, training and skills mitigated the shortcomings in knowledge and skills, enabling health care professionals to use telemedicine easily [67,68]. These potential barriers should be identified early in the process of planning the implementation of changes. Physicians’ and patients’ needs, characteristics, acceptance, and satisfaction must be further assessed through research informed by the technology acceptance model [69], unified theory of acceptance and use of technology [70], theory of planned behavior [71], and theory of organization and environment [72] and so too must the reliability, usefulness, and ease of use of technologies [73]. This will enable the formulation of strategies to avoid resistance to change [74].

Many clinical factors have been shown to influence the success of telemedicine in the WHO European Region, mainly the lack of definitive scientific evidence on its clinical contribution. Others included management, care delivery, and outcomes for a particular pathology. More research was considered necessary to provide evidence of both the clinical benefits of telemedicine and improved case monitoring [75].

Telemedicine resistance was reported as often being due to patients’ lack of confidence, lack of motivation or support, or sociocultural aspects [76-78]. A feeling of having less personal contact with the clinician, lack of access to a helpful caregiver, and face-to-face preference hindered patients’ use of telemedicine [79-81], but shortcomings in technology-related knowledge and skills posed the main challenge [62,82]. Conversely, patient motivation, engagement, and empowerment were considered the main facilitators, enabling patients to use telemedicine easily [78,83-85]. Access to a helpful caregiver, insight into the home environment, and adoption of digital culture might reduce resistance, as the pandemic has shown [86]. Patients who trusted technology and were satisfied with web-based consultations showed no resistance to telemedicine, felt safe and empowered to discuss personal issues, and had experiences similar to those with face-to-face consultations. However, better response time was one of the largest facilitators of telemedicine. Web-based consultation also promoted increased adherence, indicating a correlation between telemedicine compliance and convenience [87,88].

However, patients with disabilities or older patients encountered difficulties when using telemedicine [89], which increased their reluctance to use it. Patients’ age was also considered a potential facilitator, especially among younger individuals [90]. Frequent and multimodal communication between health care professionals and patients, as well as patients sharing their experiences, might reduce the aforementioned difficulties [91,92].

Access to funding and the high costs associated with implementation were economic barriers. Similarly, socioeconomic aspects emerged as obstacles to the functional integration of telemedicine apps. The implementation of a financial framework must be considered. However, outcomes were positive when technology was financially beneficial [93-95].

Internet access, technology development, usability, infrastructure, and interoperability were the main barriers to telemedicine intervention delivery, usability, and user satisfaction, while the availability of technology development was a mediator and facilitator [96].

The most common barriers associated with ethics, security, and privacy issues were privacy and data security and data-related regulatory concerns [97,98]. Health care professionals and patients also raised concerns about safety, especially with mobile medical apps. According to the 2015 WHO Global Survey on eHealth, 80% of the member states had laws to protect individual health data, but 53% had none in place to allow individuals to access their own data; only 43% had policies or legislation regarding medical jurisdiction, liability, or compensation [58,79]. However, only 1 study highlighted the need to establish a legal framework to ensure that new telemedicine technologies complied with the constitution, legislation, regulations, and existing contracts [49]. The WHO European Region and European Commission have focused their policies on data exchange and regulatory aspects and now offer a series of frameworks and recommendations that national health plans should include to ensure the success of telemedicine and digital health as a whole. These are the cases of the European Health Information Initiative fostered by the WHO European Region, which aims to harmonize the health information gathered in European countries, and the European Commission’s European data strategy, which promotes the creation of a single market for data, including health data [99]. However, other relevant aspects, such as clinical, organizational, and human factors, have either been disregarded or do not have a clear direction. This scenario poses a considerable challenge for the formulation of public policies and strategies by health care institutions, where decisions on telemedicine use should not be overlooked.

Finally, based on the solid effectiveness telemedicine technologies can deliver, policy makers and stakeholders should not only facilitate the implementation of these applications but also recognize and tackle drawbacks to maximize the likelihood of use success. Research is confronted with the challenge of producing such evidence, a prerequisite for the generalized adoption of telemedicine. Nevertheless, none of the included studies reached “moderate” or “high” reporting quality based on the AMSTAR 2 methodology. Studies have rarely reported items considered critical for assessing the methodological quality of systematic reviews. The existence of such reporting inappropriateness significantly affected our results, as the overall quality of the evidence was directly affected by the overall limited reporting quality of the included reviews. Notably, several other evidence makers have emphasized the occurrence of systematic reviews with poor or very poor reporting completeness [100,101]. As a partial solution to this issue, we strongly suggest the need to adhere to the basic principles used among high-quality evidence researchers, with considerable attention paid to critical features (protocol registration before project initiation, appropriate search strategy and literature search, rationale for excluding studies, risk of bias appraisal of included studies, appropriateness of meta-analytical methods [when pertinent], consideration of risk of bias in interpreting review findings, and assessment of publication bias). Thus, by appropriately using core reporting features, systematic reviews (and consequently, overviews of systematic reviews) can guide decisions on accurate, succinct, credible, and comprehensive summaries of the best available evidence on a topic.

### Limitations

A total of 5 databases were explored, focusing only on systematic reviews, meta-analyses, and bibliometric analyses, thus limiting the exhaustivity of the search. Furthermore, although we initially identified almost 1000 studies for screening, our overview found only 33 reviews meeting our inclusion and exclusion criteria. Consequently, the representativeness of our findings can be questioned considering the number of primarily identified records. However, despite using a highly sensitive search strategy, designed with collaboration between a field specialist and librarian, the “over retrieval” of records might not only associate with wrong selection of identifiers and keywords by systematic review authors but also reflect indexation issues. In addition, this could also reflect the absence of a reliable description of methods used throughout study execution (resulting in the exclusion of shortlisted records) and the scarcity of investigations on this particular subject of study. The information sources were peer-reviewed publications; therefore, some relevant information from other sources (eg, gray literature) may have been missed. Lower quality scores based on AMSTAR 2 may have reflected incomplete reports rather than unqualified review methods, such as some aspects not considered by the authors; for example, a lack of protocol registration or clarity on the characteristics of the included and excluded studies.

### Conclusions

The results underscore the need to design dynamic approaches for telemedicine interventions in the WHO European Region. Potential barriers should be identified early in the process. The barriers and facilitators identified in this overview, as well as their influence, should be further investigated because only clear evidence will support the formulation of strategies to avoid resistance to change [74]. Poorer nations should also be included to benefit from emerging health technologies and to avoid geoeconomic research bias [102-104]. The WHO European Region and European Commission have developed several initiatives to foster the development and implementation of telemedicine. These include some that are more general, such as the inclusion of telemedicine and digital health as a key aspect in their policy frameworks (eg, Global Strategy on Digital Health 2020-2025 by the WHO) and others that are more focused on implementation (Horizon 2020 and Horizon Europe funding programs and the European Reference Networks) [99]. The WHO European Region will continue leveraging the potential of telemedicine in the context of the Digital Health Action Plan for the WHO European Region (2023-2030), which was adopted in September 2022. In the context of these policy frameworks, these initiatives recognize not only the power of telemedicine to break down geographical barriers and expand access to health services but also the need for mechanisms to mitigate barriers and risks.

## Appendix

Multimedia Appendix 1 Protocol for this overview of systematic reviews published in PROSPERO (International Prospective Register of Systematic Reviews).

Multimedia Appendix 2 Search strategy.

Multimedia Appendix 3 Data extraction form.

Multimedia Appendix 4 A Measurement Tool to Assess Systematic Review 2 judgment for each included study.

Multimedia Appendix 5 Main characteristics of included reviews.

Multimedia Appendix 6 Summary of findings table for main outcomes.

Multimedia Appendix 7 Main findings from included reviews.

Multimedia Appendix 8 List of barriers, facilitators, limitations, and current challenges.

## Abbreviations

AMSTAR 2: A Measurement Tool to Assess Systematic Review 2
ICD-10: International Classification of Diseases, 10th edition
PRISMA: Preferred Reporting Items for Systematic Reviews and Meta-analyses
PROSPERO: International Prospective Register of Systematic Reviews
WHO: World Health Organization
