# Supplementary material for: The Current Status of Telemedicine Technology Use Across the World Health Organization European Region: An Overview of Systematic Reviews
Source: J Med Internet Res. 2022 Oct 27;24(10):e40877. doi: 10.2196/40877 (PMC9650581; doi:10.2196/40877)
Supplement: Multimedia Appendix 1 [file jmir_v24i10e40877_app1.pdf]

Systematic review

A list of fields that can be edited in an update can be found here

1. * Review title.

Give the title of the review in English

The status of telemedicine in the European region: a systematic review from 53 national territories.

2. Original language title.

For reviews in languages other than English, give the title in the original language. This will be displayed with the English language title.

3. * Anticipated or actual start date.

Give the date the systematic review started or is expected to start.

11/02/2022

4. * Anticipated completion date.

Give the date by which the review is expected to be completed.

11/03/2022

5. * Stage of review at time of this submission.

This field uses answers to initial screening questions. It cannot be edited until after registration.

Tick the boxes to show which review tasks have been started and which have been completed.

Update this field each time any amendments are made to a published record.

The review has not yet started: Yes

Review stage	Started	Completed
Preliminary searches	No	No
Piloting of the study selection process	No	No
Formal screening of search results against eligibility criteria	No	No
Data extraction	No	No
Risk of bias (quality) assessment	No	No
Data analysis	No	No

Provide any other relevant information about the stage of the review here.

6. * Named contact.

The named contact is the guarantor for the accuracy of the information in the register record. This may be any member of the review team.

Israel Júnior Borges do Nascimento

Email salutation (e.g. "Dr Smith" or "Joanne") for correspondence:

Dr Borges do Nascimento

7. * Named contact email.

Give the electronic email address of the named contact.

israeljrbn@gmail.com

8. Named contact address

Give the full institutional/organisational postal address for the named contact.

Medical Sciences Divisional Office - University of Oxford - Level 3, John Radcliffe Hospital Oxford OX3 9DU,

United Kingdom

9. Named contact phone number.

Give the telephone number for the named contact, including international dialling code.

+ 1 860 869 7285

10. * Organisational affiliation of the review.

Full title of the organisational affiliations for this review and website address if available. This field may be completed as 'None' if the review is not affiliated to any organisation.

Medical Sciences Divisional Office - University of Oxford

Organisation web address:

11. * Review team members and their organisational affiliations.

Give the personal details and the organisational affiliations of each member of the review team. Affiliation refers to groups or organisations to which review team members belong. **NOTE: email and country now MUST be entered for each person, unless you are amending a published record.**

Mr Israel Júnior Borges do Nascimento. Medical Sciences Divisional Office - University of Oxford
Dr Francesc Saigí Rubió. Department of Health Sciences, Universitat Oberta de Catalunya, Barcelona, Spain
Dr Noemí Robles Muñoz. eHealth Center, Universitat Oberta de Catalunya, Barcelona, Spain
Dr Che Katz. National University of Kyiv-Mohyla Academy, School of Public Health, Kyiv, Ukraine
Dr Keti Ivanovska. School Nurse, Franconian International School, Erlangen, Germany
Dr David Novillo-Ortiz. Division of Country Health Policies and Systems, World Health Organization, regional office for Europe, Copenhagen, Denmark

12. * Funding sources/sponsors.

Details of the individuals, organizations, groups, companies or other legal entities who have funded or sponsored the review.

None

Grant number(s)

State the funder, grant or award number and the date of award

None

13. * Conflicts of interest.

List actual or perceived conflicts of interest (financial or academic).

None

14. Collaborators.

Give the name and affiliation of any individuals or organisations who are working on the review but who are not listed as review team members. **NOTE: email and country must be completed for each person, unless you are amending a published record.**

15. * Review question.

State the review question(s) clearly and precisely. It may be appropriate to break very broad questions down into a series of related more specific questions. Questions may be framed or refined using PI(E)COS or similar where relevant.

What is the status of the use of telemedicine across European countries? In which medical fields and levels of care telemedicine has evidencing its effectiveness and applicability? What are the main challenges that telemedicine must overcome in order to result in the finest delivery of care? Are there recommendations to be highlighted regarding the creation, adoption, implementation, and useability of telemedicine in the European region?

16. * Searches.

State the sources that will be searched (e.g. Medline). Give the search dates, and any restrictions (e.g. language or publication date). Do NOT enter the full search strategy (it may be provided as a link or attachment below.)

Five databases will be used in this systematic review (PubMed, Embase, Web of Sciences, the Cochrane Library, and Scopus). We will include quantitative, qualitative, and mixed-method studies, regardless the publication date and primary language of publication. However, the study must adequately include and display the status of telemedicine in the respective European country or national territory. We will consider the 53 countries associated with the Regional Office for Europe registered by the World Health Organization. As the development of telemedicine tools had considerably increased with the ongoing SARS-CoV-2 pandemic as well as the waiting time for publication of manuscripts, if retrieved and eligible for inclusion, we will include preprints and unpublished data.

17. URL to search strategy.

Upload a file with your search strategy, or an example of a search strategy for a specific database, (including the keywords) in pdf or word format. In doing so you are consenting to the file being made publicly accessible. Or provide a URL or link to the strategy. Do NOT provide links to your search **results**.

Alternatively, upload your search strategy to CRD in pdf format. Please note that by doing so you are consenting to the file being made publicly accessible.

Do not make this file publicly available until the review is complete

18. * Condition or domain being studied.

Give a short description of the disease, condition or healthcare domain being studied in your systematic review.

Digital health solutions, Telemedicine, Quantitative studies, Qualitative studies, Systematic review, Evidence-based medicine.

19. * Participants/population.

Specify the participants or populations being studied in the review. The preferred format includes details of both inclusion and exclusion criteria.

We do not intend to analyze a specific population. However, we will focus on studies that directly assess the use of telemedicine tools and interfaces in 53 European territories (which can be seen as the targeted setting).

20. * Intervention(s), exposure(s).

Give full and clear descriptions or definitions of the interventions or the exposures to be reviewed. The preferred format includes details of both inclusion and exclusion criteria.

Studies that evaluate the use of telemedicine, with (telemedicine versus usual care, in all care levels) or without a suitable comparator factor.

21. * Comparator(s)/control.

Where relevant, give details of the alternatives against which the intervention/exposure will be compared (e.g. another intervention or a non-exposed control group). The preferred format includes details of both inclusion and exclusion criteria.

We will accept any type of comparator or control group, including the comparison with traditional care/face-to-face patient care. Nevertheless, we will consider eligible for inclusion studies without a control group.

22. * Types of study to be included.

Give details of the study designs (e.g. RCT) that are eligible for inclusion in the review. The preferred format includes both inclusion and exclusion criteria. If there are no restrictions on the types of study, this should be stated.

We will include both quantitative (such as randomized trials and observational studies), qualitative (studies which collect and analyze non-numerical data), and mixed-method studies, including studies under revision or published in pre-prints hosting systems.

23. Context.

Give summary details of the setting or other relevant characteristics, which help define the inclusion or exclusion criteria.

Telemedicine has substantially impacted the quality and structure of the healthcare systems and how care is subsequently delivered to a targeted population. Worldwide, there is a sensible and solid evidence suggesting the effectiveness, applicability, and status of telemedicine. However, to the best of our knowledge, no previous systematic review has collated and comprehensively summarized data from multiple studies about the status of telemedicine in the 53 European national territories. Therefore, the aim of this systematic review is to create a summary of findings regarding the status of the use of telemedicine across European countries and to identify which medical fields and levels of care telemedicine has evidencing its effectiveness and applicability. In addition, we plan to highlight the main challenges that telemedicine must overcome in order to result in the finest delivery of care and to raise recommendations associated with the creation, adoption, implementation, and useability of telemedicine.

24. * Main outcome(s).

Give the pre-specified main (most important) outcomes of the review, including details of how the outcome is defined and measured and when these measurement are made, if these are part of the review inclusion criteria.

The primary outcome to be assessed in this review will be the effectiveness, feasibility, applicability, and security of the use of telemedicine in the 53 European national territories. In addition, we will consider the following outcomes: 1. overall healthcare professionals' perception, acceptability, credibility, and satisfaction of using telemedicine tools, 2. the impact of telemedicine in improving quality of life among health professionals and patients, 3. Provider performance or any patient outcome, and 4. Economic assessment measures. Moreover, we will identify the main challenges that telemedicine must overcome to achieve the best delivery of care as well as highlight recommendation for policy makers working in the European region. It is worthwhile mentioning that we do not plan to restrict this systematic review based upon the before mentioned outcomes. Therefore, we will potentially include more outcomes than the ones initially described in this protocol.

Measures of effect

Please specify the effect measure(s) for you main outcome(s) e.g. relative risks, odds ratios, risk difference, and/or 'number needed to treat.

These characteristics must be described in the methodology and in the results of the included systematic reviews.

25. * Additional outcome(s).

List the pre-specified additional outcomes of the review, with a similar level of detail to that required for main outcomes. Where there are no additional outcomes please state 'None' or 'Not applicable' as appropriate to the review

None

Measures of effect

Please specify the effect measure(s) for you additional outcome(s) e.g. relative risks, odds ratios, risk difference, and/or 'number needed to treat.

26. * Data extraction (selection and coding).

Describe how studies will be selected for inclusion. State what data will be extracted or obtained. State how this will be done and recorded.

Studies selection (title/abstract and full text screening), data extraction, risk of bias assessment, and quality of evidence rating will be performed by two individual researchers. Interrater conflicts will be resolved by discussion or a third researcher. We plan to provide a supplementary material list showing the reasons for exclusion of studies short-listed in the full-text screening phase. The following data will be extracted, but no restricted to: study identification (first author, year of publication, country where the study was carried out), study design, telemedicine type, aim of the study, sample size, assessment period, and outcomes evaluated. Based on the methodological similarities across studies deemed eligible for inclusion as well as outcome type and measurement units, we plan to conduct a statistical assessment associated with meta-analysis. These analyses will be conducted using Review Manager v.5.4.1 (RevMan) software or in the R software using specific packages.

27. * Risk of bias (quality) assessment.

State which characteristics of the studies will be assessed and/or any formal risk of bias/quality assessment tools that will be used.

Randomized clinical trials will be assessed using the Cochrane risk of bias approach, while observational studies will be evaluated using the Risk of Bias In Non-randomized Studies of Interventions (ROBINS-I). In addition, mixed-methods studies will be appraised using the “mixed methods appraisal tool, version 18; and qualitative studies will be assessed using the COREQ scale.

28. * Strategy for data synthesis.

Describe the methods you plan to use to synthesise data. This **must not be generic text** but should be **specific to your review** and describe how the proposed approach will be applied to your data. If meta-analysis is planned, describe the models to be used, methods to explore statistical heterogeneity, and software package to be used.

If we retrieve and include enough records to be combined quantitively, we plan to use a fixed-effect model in the first instance to combine the data. For any meta-analyses, we plan to collate the extracted data using risk ratio, risk difference, number needed to treat, number needed to harm, mean difference, and 95% confidence intervals. Moreover, we plan to evaluate and interpret heterogeneity among retrieved studies using I^2, considering substantial heterogeneity if $I^2$50%. However, if not possible, we plan to create a qualitative synthesis, reporting and combining individual main results from studies, classified into groups of intervention. Moreover, we will create a graphic art (using Adobe Photoshop) with all identified results, to be included supplementary in the results section.

29. * Analysis of subgroups or subsets.

State any planned investigation of 'subgroups'. Be clear and specific about which type of study or participant will be included in each group or covariate investigated. State the planned analytic approach.

None

30. * Type and method of review.

Select the type of review, review method and health area from the lists below.

Type of review

Cost effectiveness
No

Diagnostic
No

Epidemiologic
Yes

Individual patient data (IPD) meta-analysis
No

Intervention
Yes

Living systematic review
No

Meta-analysis
No

Methodology
No

Narrative synthesis
Yes

Network meta-analysis
No

Pre-clinical
No

Prevention
No

Prognostic
No

Prospective meta-analysis (PMA)
No

Review of reviews
No

Service delivery
Yes

Synthesis of qualitative studies
Yes

Systematic review
Yes

Other
No

Health area of the review

Alcohol/substance misuse/abuse
No

Blood and immune system
No

Cancer
No

Cardiovascular
No

Care of the elderly
No

Child health
No

Complementary therapies
No

COVID-19
No

Crime and justice
No

Dental
No

Digestive system
No

Ear, nose and throat
No

Education
No

Endocrine and metabolic disorders
No

Eye disorders
No

General interest
Yes

Genetics
No

Health inequalities/health equity
No

Infections and infestations

No

International development
Yes

Mental health and behavioural conditions
No

Musculoskeletal
No

Neurological
No

Nursing
No

Obstetrics and gynaecology
No

Oral health
No

Palliative care
No

Perioperative care
No

Physiotherapy
No

Pregnancy and childbirth
No

Public health (including social determinants of health)
Yes

Rehabilitation
No

Respiratory disorders
No

Service delivery
No

Skin disorders
No

Social care
No

Surgery
No

Tropical Medicine
No

Urological
No

Wounds, injuries and accidents
No

Violence and abuse
No

31. Language.

Select each language individually to add it to the list below, use the bin icon to remove any added in error.
English

There is not an English language summary

32. * Country.

Select the country in which the review is being carried out. For multi-national collaborations select all the countries involved.

Brazil
Denmark
England
Germany
Ukraine

33. Other registration details.

Name any other organisation where the systematic review title or protocol is registered (e.g. Campbell, or The Joanna Briggs Institute) together with any unique identification number assigned by them. If extracted data will be stored and made available through a repository such as the Systematic Review Data Repository (SRDR), details and a link should be included here. If none, leave blank.

34. Reference and/or URL for published protocol.

If the protocol for this review is published provide details (authors, title and journal details, preferably in Vancouver format)

Add web link to the published protocol.

Or, upload your published protocol here in pdf format. Note that the upload will be publicly accessible.

No I do not make this file publicly available until the review is complete

Please note that the information required in the PROSPERO registration form must be completed in full even if access to a protocol is given.

35. Dissemination plans.

Do you intend to publish the review on completion?

Yes

Give brief details of plans for communicating review findings.?

The paper will be potentially submitted to the Journal of Telemedicine and Telecare.

36. Keywords.

Give words or phrases that best describe the review. Separate keywords with a semicolon or new line. Keywords help PROSPERO users find your review (keywords do not appear in the public record but are included in searches). Be as specific and precise as possible. Avoid acronyms and abbreviations unless these are in wide use.

Digital health solutions, telemedicine, quantitative studies, qualitative studies, systematic review, overview, evidence-based medicine.

37. Details of any existing review of the same topic by the same authors.

If you are registering an update of an existing review give details of the earlier versions and include a full bibliographic reference, if available.

There are no pre-existing reviews of the same topic carried out by the authors of the proposed Review.

38. * Current review status.

Update review status when the review is completed and when it is published.New registrations must be ongoing so this field is not editable for initial submission.
Please provide anticipated publication date

Review_Ongoing

39. Any additional information.

Provide any other information relevant to the registration of this review.

40. Details of final report/publication(s) or preprints if available.

Leave empty until publication details are available OR you have a link to a preprint (NOTE: this field is not editable for initial submission). List authors, title and journal details preferably in Vancouver format.

Give the link to the published review or preprint.
