# Supplementary material for: The Current Status of Telemedicine Technology Use Across the World Health Organization European Region: An Overview of Systematic Reviews
Source: J Med Internet Res. 2022 Oct 27;24(10):e40877. doi: 10.2196/40877 (PMC9650581; doi:10.2196/40877)
Supplement: Multimedia Appendix 2 [file jmir_v24i10e40877_app2.docx]

**Multimedia Appendix 2 – Search Strategy used in each scientific database**

Search details for

**The current state of use of telemedicine technologies across the European Region: an overview of systematic reviews**

Topic and methodology experts: Israel Júnior Borges do Nascimento, Francesc Saigí-Rubió, and David Novillo-Ortiz

Date: January 21, 2022

Databases: Based on the initial search strategy used for PubMed (below), we performed the “adaptation and translation” of each entry term for the other databases (Embase, Scopus, The Cochrane Library, and Web of Science). Full library containing all studies from the five databases can be access upon request.

**# Terms related to “Telemedicine” #**

(Telemedicine[TITLE/ABSTRACT]) OR (Telehealth[TITLE/ABSTRACT]) OR (Remote Consultation[TITLE/ABSTRACT]) OR (Consultation*, Remote[TITLE/ABSTRACT]) OR (Teleconsultation*[TITLE/ABSTRACT]) OR (Telepathology[TITLE/ABSTRACT]) OR (Teleradiology[TITLE/ABSTRACT]) OR (Telerehabilitation[TITLE/ABSTRACT]) OR (Telerehabilitation*[TITLE/ABSTRACT]) OR (Tele-rehabilitation*[TITLE/ABSTRACT]) OR (Remote Rehabilitation*[TITLE/ABSTRACT]) OR (Rehabilitation*, Remote [TITLE/ABSTRACT]) OR (Virtual Rehabilitation*[TITLE/ABSTRACT]) OR (Rehabilitation*, Virtual[TITLE/ABSTRACT]) OR (Teledentistry[TITLE/ABSTRACT]) OR (Telecare[TITLE/ABSTRACT]) OR (Online Consultation*[TITLE/ABSTRACT]) OR (Tele-medicine[TITLE/ABSTRACT]) OR (Tele-health[TITLE/ABSTRACT]) OR (Remote Health[TITLE/ABSTRACT]) OR (Virtual Health[TITLE/ABSTRACT]) OR (Mobile Medicine[TITLE/ABSTRACT]) OR (Remote Medicine[TITLE/ABSTRACT]) OR (Virtual Medicine[TITLE/ABSTRACT]) OR (Telemonitoring[TITLE/ABSTRACT]) OR (Remote Monitoring[TITLE/ABSTRACT]) AND ((Albania[TITLE/ABSTRACT]) OR (Andorra[TITLE/ABSTRACT]) OR (Armenia[TITLE/ABSTRACT]) OR (Austria[TITLE/ABSTRACT]) OR (Azerbaijan[TITLE/ABSTRACT]) OR (Belarus[TITLE/ABSTRACT]) OR (Belgium[TITLE/ABSTRACT]) OR (Bosnia and Herzegovina[TITLE/ABSTRACT]) OR (Bulgaria[TITLE/ABSTRACT]) OR (Croatia[TITLE/ABSTRACT]) OR (Cyprus[TITLE/ABSTRACT]) OR (Czechia[TITLE/ABSTRACT]) OR (Denmark[TITLE/ABSTRACT]) OR (Estonia[TITLE/ABSTRACT]) OR (Finland[TITLE/ABSTRACT]) OR (France[TITLE/ABSTRACT]) OR (Georgia[TITLE/ABSTRACT]) OR (Germany[TITLE/ABSTRACT]) OR (Greece[TITLE/ABSTRACT]) OR (Hungary[TITLE/ABSTRACT]) OR (Iceland[TITLE/ABSTRACT]) OR (Ireland[TITLE/ABSTRACT]) OR (Israel [TITLE/ABSTRACT]) OR (Italy[TITLE/ABSTRACT]) OR (Kazakhstan[TITLE/ABSTRACT]) OR (Kyrgyzstan[TITLE/ABSTRACT]) OR (Latvia[TITLE/ABSTRACT]) OR (Lithuania[TITLE/ABSTRACT]) OR (Luxembourg[TITLE/ABSTRACT]) OR (Malta[TITLE/ABSTRACT]) OR (Monaco[TITLE/ABSTRACT]) OR (Montenegro[TITLE/ABSTRACT]) OR (Netherlands[TITLE/ABSTRACT]) OR (North Macedonia[TITLE/ABSTRACT]) OR (Norway[TITLE/ABSTRACT]) OR (Poland[TITLE/ABSTRACT]) OR (Portugal[TITLE/ABSTRACT]) OR (Moldova[TITLE/ABSTRACT]) OR (Romania[TITLE/ABSTRACT]) OR (Russia[TITLE/ABSTRACT]) OR (San Marino[TITLE/ABSTRACT]) OR (Serbia[TITLE/ABSTRACT]) OR (Slovakia[TITLE/ABSTRACT]) OR (Slovenia[TITLE/ABSTRACT]) OR (Spain[TITLE/ABSTRACT]) OR (Sweden[TITLE/ABSTRACT]) OR (Switzerland[TITLE/ABSTRACT]) OR (Tajikistan[TITLE/ABSTRACT]) OR (Turkey[TITLE/ABSTRACT]) OR (Turkmenistan[TITLE/ABSTRACT]) OR (Ukraine[TITLE/ABSTRACT]) OR (United Kingdom[TITLE/ABSTRACT]) OR (Uzbekistan[TITLE/ABSTRACT]) OR (europe*[TITLE/ABSTRACT])))

**# Terms related to “Evidence-based Medicine” #**

AND ((systematic review[Title/Abstract]) OR ((systematic OR scoping[TITLE/ABSTRACT]) AND (review* OR overview*[TITLE/ABSTRACT])) OR (meta analy*[TITLE/ABSTRACT]) OR (metaanaly* OR meta-analy*[TITLE/ABSTRACT]))
