# Supplementary material for: The Current Status of Telemedicine Technology Use Across the World Health Organization European Region: An Overview of Systematic Reviews
Source: J Med Internet Res. 2022 Oct 27;24(10):e40877. doi: 10.2196/40877 (PMC9650581; doi:10.2196/40877)
Supplement: Multimedia Appendix 3 [file jmir_v24i10e40877_app3.docx]

**Multimedia Appendix 3 – Data extraction form**

| Column | Procedure |
| --- | --- |
| A – Study Title | Already extracted - None |
| B – Study ID, Publication Year | Already extracted - None |
| C – Journal Name | Already extracted - None |
| D – Included Countries | Please, insert the fully written countries’ names (not abbreviated) considered in the manuscript. Note that in several selected records included studies outside the EU. Please, identify these studies with a “*” and double-check if the study enrolls ≥ 50% of countries within the 53 WHO recognized EU countries. |
| E – Objective | Simply copy and paste the studies’ main objective. |
| F – Telemedicine Specialty | Please, insert the telemedicine specialties considered in the manuscript (i.e., teleophthalmology, telepathology, teledermatology, teleendoscopy, telepediatrics, telepsychiatry, telesurgery, teleotorhinolaryngology, teleneurophysiology, telemonitoring of diabetes and dialysis, teleoncology, telecardiology). |
| G – Medical Specialty or Medical Focus | Using the data validation list, select to which category the respective study is associated with in accordance to the ICD-10 classification list. For instance, if the study is evaluating the use of telemedicine in the stroke care, please select “Chapter 9 – Diseases of the circulatory system”. You can easily identify to which category a certain disease belongs to on the internet, by typing “disease/condition + ICD10”. For those studies addressing multiple diseases/conditions, kindly select the option “Multifocal”. |
| H – Setting, Scenario | Briefly describe the setting or scenario primarily focused on the study. |
| I – Included Databases | Kindly describe which databases were considered in the article. Please, do not abbreviate the name of the databases. |
| J – Number of Included Studies | Describe the number of included studies considered in the review (either quantitatively or qualitatively). |
| K – Primary Studies Design | List the primary studies’ designs considered in the review. |
| L – Publication Design | Using the data validation list, select to which publication type the study is related to. |
| M – Data Type and Collection | Type of data used in the study (vital signs, laboratory results, time of treatment, sociodemographic data, etc.) |
| N – Main results | List the main findings highlighted in the study. This might be a little bit challenging, as some studies tend to have an extensive result section. I usually share the following rationale with my colleagues while extracting data – “just include data that will attract the future reader and give knowledge to healthcare providers, policy-makers, and the scientific community”. |
| O – Listed Barriers | In this category, please insert the barriers associated with the telemedicine implementation process emphasized within the study. |
| P – Highlighted Facilitators | Please describe here the drivers (if any) that have facilitated the implementation and development of the telemedicine service (i.e., end-users have been included in the identification of key telemedicine functions; Strong, visible, and proactive leadership; etc.). |
| Q – Main Limitations Pinpointed | List the manuscript limitations. Frequently, high-quality systematic reviews describe the studies’ limitation in the last 2 paragraphs of the discussion section. If not or if you consider relevant, please, elaborate additional limitation and pitfalls. |
| R – Current Challenges | List the challenges of the use of telemedicine in the EU countries evidenced by the article. |
| S – Evaluation of Telemedicine Intervention | Please, describe here which mechanism of evaluation has been used to assess the telemedicine intervention (i.e., assessment based on theoretical frameworks and models (TAM, UTAUT…) to provide justification of hypothetical relationships and a guide for its verification; retrospective studies using large/short samples; survey research; case studies; triangulation and qualitative methods; assessment about technological infrastructure, physicians’ roles, etc.). |
| T – AMSTAR 2 Rate | Once we have the data extraction sheet completed, we can provide these data. |
