# Supplementary material for: The Current Status of Telemedicine Technology Use Across the World Health Organization European Region: An Overview of Systematic Reviews
Source: J Med Internet Res. 2022 Oct 27;24(10):e40877. doi: 10.2196/40877 (PMC9650581; doi:10.2196/40877)
Supplement: Multimedia Appendix 4 [file jmir_v24i10e40877_app4.docx]

Multimedia Appendix 4 – AMSTAR judgment for each included study

Table of Contents

[Study 1 – Allner et al 3](#_Toc105504849)

[Study 2 – Brunetti et al 7](#_Toc105504850)

[Study 3 – Carbo et al 11](#_Toc105504851)

[Study 4 – Cordes et al 15](#_Toc105504852)

[Study 5 – Cruz et al 19](#_Toc105504853)

[Study 6 – Elbaz et al 23](#_Toc105504854)

[Study 7 – Farabi et al 27](#_Toc105504855)

[Study 8 – Gaveikate et al 31](#_Toc105504856)

[Study 9 – Glinkowski et al 35](#_Toc105504857)

[Study 10 – Hallesleben et al 39](#_Toc105504858)

[Study 11 – Hartasanchez et al 43](#_Toc105504859)

[Study 12 – Hrynyschyn et al 47](#_Toc105504860)

[Study 13 – Karamanidou et al 51](#_Toc105504861)

[Study 14 – Keirkegaard et al 55](#_Toc105504862)

[Study 15 – Kingsdorf et al 59](#_Toc105504863)

[Study 16 - Labiris et al 63](#_Toc105504864)

[Study 17 - Maresca et al 67](#_Toc105504865)

[Study 18 – Martin et al 71](#_Toc105504866)

[Study 19 - McFarland et al 75](#_Toc105504867)

[Study 20 – Mold et al 79](#_Toc105504868)

[Study 21 – Nielsen et al 83](#_Toc105504869)

[Study 22 – OCathail et al 87](#_Toc105504870)

[Study 23 - Ohannessiam et al 91](#_Toc105504871)

[Study 24 – Pron et al 95](#_Toc105504872)

[Study 25 - Raja et al 99](#_Toc105504873)

[Study 26 – Simmonds-Buckley 2020 103](#_Toc105504874)

[Study 27 – Singh 2016 107](#_Toc105504875)

[Study 28 - Tokgoz 2022 111](#_Toc105504876)

[Study 29 – Trettel 2017 115](#_Toc105504877)

[Study 30 – Udsen et al 119](#_Toc105504878)

[Study 31 – Verma et al 123](#_Toc105504879)

[Study 32 - Willard et al 127](#_Toc105504880)

[Study 33 - Zanin et al 131](#_Toc105504881)

# Study 1 – Allner et al

| **1. Did the research questions and inclusion criteria for the review include the components of PICO?** | | | | | | | | |
| --- | --- | --- | --- | --- | --- | --- | --- | --- |
| For Yes:  🗷 Population  🗷 Intervention   Comparator group  🗷 Outcome | | | Optional (recommended)   Timeframe for follow-up | | 🗷 | | Yes No |  |
| Comments: In addition to the standard PICO features, a S standing for “setting” could be observed in this study, particularly related to rural areas of Germany. No comparator is applied for this review. | | | | | | | |  |
| **2. Did the report of the review contain an explicit statement that the review methods were established prior to the conduct of the review and did the report justify any significant deviations from the protocol?** | | | | | | | | |
|  | For Partial Yes:  The authors state that they had a written protocol or guide that included ALL the following:  🗷 review question(s)  🗷 a search strategy  🗷 inclusion/exclusion criteria  🗷 a risk of bias assessment | | For Yes:  As for partial yes, plus the protocol should be registered and should also have specified:   a meta-analysis/synthesis plan, if appropriate, *and*   a plan for investigating causes of heterogeneity   justification for any deviations from the protocol | |   🗷 | | Yes  Partial Yes  No |  |
|  | Comments: Authors stated that the protocol and additional information can be access upon request. However, no record locator is presented in the text. Therefore, we considered that a review protocol was not published. In addition, the search strategy presented was not supported by an experienced librarian and could be considered simple. The authors used the MAST approach to assess the study quality and the model of telemedicine. | | | | | | |  |
| **3. Did the review authors explain their selection of the study designs for inclusion in the review?** | | | | | | | | |
|  | For Yes, the review should satisfy ONE of the following:   *Explanation for* including only RCTs   OR *Explanation for* including only NRSI   OR *Explanation for* including both RCTs and NRSI | | | | 🗷 | | Yes No |  |
|  | Comments: There were no considerations presented in term of study designs of potentially eligible studies. | | | | | | |  |
| **4. Did the review authors use a comprehensive literature search strategy?** | | | | | | | | |
|  | For Partial Yes (all the following): | | For Yes, should also have (all the following):  🗷 searched the reference lists / bibliographies of included studies   searched trial/study registries   included/consulted content experts in the field  🗷 where relevant, searched for grey literature   conducted search within 24 months of completion of the review | |  | |  |  |
|  | 🗷 searched at least 2 databases (relevant to research question)  🗷 provided key word and/or search strategy  🗷 justified publication restrictions | |  |  |   🗷 | | Yes  Partial Yes No |  |
|  | (e.g. language) | |  |  |  | |  |  |
|  | Comments: There were no publication restrictions. | | | | | | |  |
|  | **5. Did the review authors perform study selection in duplicate?** | | | |  | | |  |
|  | For Yes, either ONE of the following:  🗷 at least two reviewers independently agreed on selection of eligible studies and achieved consensus on which studies to include   OR two reviewers selected a sample of eligible studies and achieved good agreement (at least 80 percent), with the remainder selected by one reviewer. | | | | 🗷 | | Yes No |  |
|  | Comments: None. | | | | | | |  |
| **6. Did the review authors perform data extraction in duplicate?** | | | | | | | | |
| For Yes, either ONE of the following:  🗷 at least two reviewers achieved consensus on which data to extract from included studies   OR two reviewers extracted data from a sample of eligible studies and achieved good agreement (at least 80 percent), with the remainder extracted by one reviewer. | | | | | | 🗷 Yes   No | | |
| Comments: | | | | | | | | |
| **7. Did the review authors provide a list of excluded studies and justify the exclusions?** | | | | | | | | |
|  | | For Partial Yes:   provided a list of all potentially relevant studies that were read in full-text form but excluded from the review | | For Yes, must also have:   Justified the exclusion from the review of each potentially relevant study | |  Yes   Partial Yes  🗷 No | | |
|  | | Comments: No list of excluded studies was presented. | | | | | | |
| **8. Did the review authors describe the included studies in adequate detail?** | | | | | | | | |
|  | | For Partial Yes (ALL the following):   described populations   described interventions   described comparators   described outcomes   described research designs | | For Yes, should also have ALL the following:   described population in detail   described intervention in detail (including doses where relevant)   described comparator in detail (including doses where relevant)   described study’s setting   timeframe for follow-up | |  Yes   Partial Yes  🗷 No | | |
|  | | Comments: Included studies were poorly described. In addition, there is not any table or supplementary material including basic studies’ characteristics. | | | | | | |
| **9. Did the review authors use a satisfactory technique for assessing the risk of bias (RoB) in individual studies that were included in the review?** | | | | | | | | |
|  | | **RCTs**  For Partial Yes, must have assessed RoB from   unconcealed allocation, *and*   lack of blinding of patients and assessors when assessing outcomes (unnecessary for objective outcomes such as all-cause mortality) | | For Yes, must also have assessed RoB from:   allocation sequence that was not truly random, *and*   selection of the reported result from among multiple measurements or analyses of a specified outcome | |  Yes   Partial Yes   No  🗷 Includes only NRSI | | |
|  | | **NRSI**  For Partial Yes, must have assessed RoB:   from confounding, *and*   from selection bias | | For Yes, must also have assessed RoB:   methods used to ascertain exposures and outcomes, *and*   selection of the reported result from among multiple measurements or analyses of a specified | |  Yes   Partial Yes  🗷 No  Includes only RCTs | | |
| Comments: The authors used the MAST approach for assessing the overall quality of studies associated with telemedicine. This methodology does not consider any of the highlighted features beforementioned. Therefore, we considered this item as “not applicable”. | | | | | | | | |

| **10. Did the review authors report outcomes on the sources of funding for the studies included in the review?** | | | | | |  |
| --- | --- | --- | --- | --- | --- | --- |
| For Yes:   Must have reported on the sources of funding for individual studies included in the review.  Note: Reporting that the reviewers looked for this information but it was not reported by study authors also qualifies. | | | |  Yes  🗷 No |  |  |
| Comments: None | | | |  |  |  |
| **11. If meta-analysis was performed did the review authors use appropriate methods for statistical combination of results?** | | | | | |  |
|  | **RCTs**  For Yes:   The authors justified combining the data in a meta-analysis   AND they used an appropriate weighted technique to combine study results and adjusted for heterogeneity if present.   AND investigated the causes of any heterogeneity | |  Yes   No  🗷 No meta-analysis conducted | | |  |
|  | **For NRSI**  For Yes:   The authors justified combining the data in a meta-analysis   AND they used an appropriate weighted technique to combine study results, adjusting for heterogeneity if present   AND they statistically combined effect estimates from NRSI that were adjusted for confounding, rather than combining raw data, or justified combining raw data when adjusted effect estimates were not available   AND they reported separate summary estimates for RCTs and NRSI separately when both were included in the review | |  Yes   No  🗷 No meta-analysis conducted | | |  |
|  | Comments: | | | | |  |
| **12. If meta-analysis was performed, did the review authors assess the potential impact of RoB in individual studies on the results of the meta-analysis or other evidence synthesis?** | | | | | |  |
|  | For Yes:   included only low risk of bias RCTs   OR, if the pooled estimate was based on RCTs and/or NRSI at variable RoB, the authors performed analyses to investigate possible impact of RoB on summary estimates of effect. | |  Yes   No  🗷 No meta-analysis conducted | | |  |
|  | Comments: | | | | |  |
| **13. Did the review authors account for RoB in individual studies when interpreting/ discussing the results of the review?** | | | | | |  |
|  | For Yes:   included only low risk of bias RCTs   OR, if RCTs with moderate or high RoB, or NRSI were included the review provided a discussion of the likely impact of RoB on the results | |  Yes  🗷 No | | |  |
|  | Comments: None | | | | |  |
| **14. Did the review authors provide a satisfactory explanation for, and discussion of, any heterogeneity observed in the results of the review?** | | | | | |  |
|  | For Yes:   There was no significant heterogeneity in the results   OR if heterogeneity was present the authors performed an investigation of sources of any heterogeneity in the results and discussed the impact of this on the results of the review | |  Yes  🗷 No | | |  |
|  | Comments: None | | | | |  |
| **15. If they performed quantitative synthesis did the review authors carry out an adequate investigation of publication bias (small study bias) and discuss its likely impact on the results of the review?** | | | | | |  |
|  | For Yes:   performed graphical or statistical tests for publication bias and discussed the likelihood and magnitude of impact of publication bias | |  Yes   No  🗷 No meta-analysis conducted | | |  |
|  | Comments: | | | | |  |
| **16. Did the review authors report any potential sources of conflict of interest, including any funding they received for conducting the review?** | | | | | |  |
|  | For Yes:  🗷 The authors reported no competing interests OR   The authors described their funding sources and how they managed potential conflicts of interest | 🗷 Yes   No | | | |  |
|  | Comments: None | | | | |  |

# Study 2 – Brunetti et al

| **1. Did the research questions and inclusion criteria for the review include the components of PICO?** | | | | | | | | |
| --- | --- | --- | --- | --- | --- | --- | --- | --- |
| For Yes:  🗷 Population  🗷 Intervention   Comparator group  🗷 Outcome | | | Optional (recommended)   Timeframe for follow-up | | 🗷 | | Yes No |  |
| Comments: No comparator was considered in the analysis. | | | | | | | |  |
| **2. Did the report of the review contain an explicit statement that the review methods were established prior to the conduct of the review and did the report justify any significant deviations from the protocol?** | | | | | | | | |
|  | For Partial Yes:  The authors state that they had a written protocol or guide that included ALL the following:   review question(s)   a search strategy   inclusion/exclusion criteria   a risk of bias assessment | | For Yes:  As for partial yes, plus the protocol should be registered and should also have specified:   a meta-analysis/synthesis plan, if appropriate, *and*   a plan for investigating causes of heterogeneity   justification for any deviations from the protocol | |   🗷 | | Yes  Partial Yes  No |  |
|  | Comments: No protocol locator was presented in the manuscript. | | | | | | |  |
| **3. Did the review authors explain their selection of the study designs for inclusion in the review?** | | | | | | | | |
|  | For Yes, the review should satisfy ONE of the following:   *Explanation for* including only RCTs  🗷 OR *Explanation for* including only NRSI   OR *Explanation for* including both RCTs and NRSI | | | | 🗷 | | Yes No |  |
|  | Comments: Based on the quality assessment approach, we inferred that only NRSI was included. | | | | | | |  |
| **4. Did the review authors use a comprehensive literature search strategy?** | | | | | | | | |
|  | For Partial Yes (all the following): | | For Yes, should also have (all the following):  🗷 searched the reference lists / bibliographies of included studies   searched trial/study registries   included/consulted content experts in the field   where relevant, searched for grey literature   conducted search within 24 months of completion of the review | |  | |  |  |
|  | 🗷 searched at least 2 databases (relevant to research question)  🗷 provided key word and/or search strategy  🗷 justified publication restrictions | |  |  |   🗷 | | Yes  Partial Yes No |  |
|  | (e.g. language) | |  |  |  | |  |  |
|  | Comments: None. | | | | | | |  |
|  | **5. Did the review authors perform study selection in duplicate?** | | | |  | | |  |
|  | For Yes, either ONE of the following:  🗷 at least two reviewers independently agreed on selection of eligible studies and achieved consensus on which studies to include   OR two reviewers selected a sample of eligible studies and achieved good agreement (at least 80 percent), with the remainder selected by one reviewer. | | | | 🗷 | | Yes No |  |
|  | Comments: None. | | | | | | |  |
| **6. Did the review authors perform data extraction in duplicate?** | | | | | | | | |
| For Yes, either ONE of the following:  🗷 at least two reviewers achieved consensus on which data to extract from included studies   OR two reviewers extracted data from a sample of eligible studies and achieved good agreement (at least 80 percent), with the remainder extracted by one reviewer. | | | | | | 🗷 Yes   No | | |
| Comments: None. | | | | | | | | |
| **7. Did the review authors provide a list of excluded studies and justify the exclusions?** | | | | | | | | |
|  | | For Partial Yes:   provided a list of all potentially relevant studies that were read in full-text form but excluded from the review | | For Yes, must also have:   Justified the exclusion from the review of each potentially relevant study | |  Yes   Partial Yes  🗷 No | | |
|  | | Comments: No list of excluded studies is presented. | | | | | | |
| **8. Did the review authors describe the included studies in adequate detail?** | | | | | | | | |
|  | | For Partial Yes (ALL the following):   described populations   described interventions   described comparators   described outcomes   described research designs | | For Yes, should also have ALL the following:   described population in detail   described intervention in detail (including doses where relevant)   described comparator in detail (including doses where relevant)   described study’s setting   timeframe for follow-up | |  Yes   Partial Yes  🗷 No | | |
|  | | Comments: Included studies main characteristics is not presented whatsoever. | | | | | | |
| **9. Did the review authors use a satisfactory technique for assessing the risk of bias (RoB) in individual studies that were included in the review?** | | | | | | | | |
|  | | **RCTs**  For Partial Yes, must have assessed RoB from   unconcealed allocation, *and*   lack of blinding of patients and assessors when assessing outcomes (unnecessary for objective outcomes such as all-cause mortality) | | For Yes, must also have assessed RoB from:   allocation sequence that was not truly random, *and*   selection of the reported result from among multiple measurements or analyses of a specified outcome | |  Yes   Partial Yes   No  🗷 Includes only NRSI | | |
|  | | **NRSI**  For Partial Yes, must have assessed RoB:   from confounding, *and*   from selection bias | | For Yes, must also have assessed RoB:  🗷 methods used to ascertain exposures and outcomes, *and*  🗷 selection of the reported result from among multiple measurements or analyses of a specified | | 🗷 Yes   Partial Yes   No  Includes only RCTs | | |
| Comments: None. | | | | | | | | |

| **10. Did the review authors report outcomes on the sources of funding for the studies included in the review?** | | | | | | |  |  |
| --- | --- | --- | --- | --- | --- | --- | --- | --- |
| For Yes:   Must have reported on the sources of funding for individual studies included in the review.  Note: Reporting that the reviewers looked for this information but it was not reported by study authors also qualifies. | | | |  Yes  🗷 No |  | |  |  |
| Comments: None. | | | |  |  | |  |  |
| **11. If meta-analysis was performed did the review authors use appropriate methods for statistical combination of results?** | | | | | | |  |  |
|  | **RCTs**  For Yes:   The authors justified combining the data in a meta-analysis   AND they used an appropriate weighted technique to combine study results and adjusted for heterogeneity if present.   AND investigated the causes of any heterogeneity | |  Yes   No   No meta-analysis conducted | | | |  |  |
|  | **For NRSI**  For Yes:  🗷 The authors justified combining the data in a meta-analysis   AND they used an appropriate weighted technique to combine study results, adjusting for heterogeneity if present   AND they statistically combined effect estimates from NRSI that were adjusted for confounding, rather than combining raw data, or justified combining raw data when adjusted effect estimates were not available   AND they reported separate summary estimates for RCTs and NRSI separately when both were included in the review | | 🗷 Yes   No   No meta-analysis conducted | | | |  |  |
|  | Comments: None. | | | | |  |  |  |
| **12. If meta-analysis was performed, did the review authors assess the potential impact of RoB in individual studies on the results of the meta-analysis or other evidence synthesis?** | | | | | | |  |  |
|  | For Yes:   included only low risk of bias RCTs   OR, if the pooled estimate was based on RCTs and/or NRSI at variable RoB, the authors performed analyses to investigate possible impact of RoB on summary estimates of effect. | |  Yes  🗷 No   No meta-analysis conducted | | | |  |  |
|  | Comments: None. | | | | |  |  |  |
| **13. Did the review authors account for RoB in individual studies when interpreting/ discussing the results of the review?** | | | | | | |  |  |
|  | For Yes:   included only low risk of bias RCTs   OR, if RCTs with moderate or high RoB, or NRSI were included the review provided a discussion of the likely impact of RoB on the results | |  Yes  🗷 No | | | |  |  |
|  | Comments: None. | | | | |  |  |  |
| **14. Did the review authors provide a satisfactory explanation for, and discussion of, any heterogeneity observed in the results of the review?** | | | | | | |  |  |
|  | For Yes:   There was no significant heterogeneity in the results   OR if heterogeneity was present the authors performed an investigation of sources of any heterogeneity in the results and discussed the impact of this on the results of the review | |  Yes  🗷 No | | | |  |  |
|  | Comments: No discussion of heterogeneity and its impact in the obtained results was given. | | | | |  |  |  |
| **15. If they performed quantitative synthesis did the review authors carry out an adequate investigation of publication bias (small study bias) and discuss its likely impact on the results of the review?** | | | | | | |  |  |
|  | For Yes:  🗷 performed graphical or statistical tests for publication bias and discussed the likelihood and magnitude of impact of publication bias | | 🗷 Yes   No   No meta-analysis conducted | | | |  |  |
|  | Comments: None. | | | | |  |  |  |
| **16. Did the review authors report any potential sources of conflict of interest, including any funding they received for conducting the review?** | | | | | | |  |  |
|  | For Yes:  🗷 The authors reported no competing interests OR   The authors described their funding sources and how they managed potential conflicts of interest | 🗷 Yes   No | | | | |  |  |
|  | Comments: None. | | | | |  | | |

# Study 3 – Carbo et al

| **1. Did the research questions and inclusion criteria for the review include the components of PICO?** | | | | | | | | |
| --- | --- | --- | --- | --- | --- | --- | --- | --- |
| For Yes:  🗷 Population  🗷 Intervention   Comparator group  🗷 Outcome | | | Optional (recommended)   Timeframe for follow-up | | 🗷 | | Yes No |  |
| Comments: Comparator not applied to this study. | | | | | | | |  |
| **2. Did the report of the review contain an explicit statement that the review methods were established prior to the conduct of the review and did the report justify any significant deviations from the protocol?** | | | | | | | | |
|  | For Partial Yes:  The authors state that they had a written protocol or guide that included ALL the following:  🗷 review question(s)   a search strategy  🗷 inclusion/exclusion criteria  🗷 a risk of bias assessment | | For Yes:  As for partial yes, plus the protocol should be registered and should also have specified:  🗷 a meta-analysis/synthesis plan, if appropriate, *and*  🗷 a plan for investigating causes of heterogeneity   justification for any deviations from the protocol | |   🗷 | | Yes  Partial Yes  No |  |
|  | Comments: Protocol registered on PROSPERO. Search strategy not presented but available upon request | | | | | | |  |
| **3. Did the review authors explain their selection of the study designs for inclusion in the review?** | | | | | | | | |
|  | For Yes, the review should satisfy ONE of the following:  🗷 *Explanation for* including only RCTs   OR *Explanation for* including only NRSI   OR *Explanation for* including both RCTs and NRSI | | | | 🗷 | | Yes No |  |
|  | Comments: None | | | | | | |  |
| **4. Did the review authors use a comprehensive literature search strategy?** | | | | | | | | |
|  | For Partial Yes (all the following): | | For Yes, should also have (all the following):  🗷 searched the reference lists / bibliographies of included studies   searched trial/study registries   included/consulted content experts in the field   where relevant, searched for grey literature   conducted search within 24 months of completion of the review | |  | |  |  |
|  | 🗷 searched at least 2 databases (relevant to research question)   provided key word and/or search strategy  🗷 justified publication restrictions | |  |  |   🗷 | | Yes  Partial Yes No |  |
|  | (e.g. language) | |  |  |  | |  |  |
|  | Comments: Keywords just available upon request, therefore it is challenging to affirm the adequate existence of this item. | | | | | | |  |
|  | **5. Did the review authors perform study selection in duplicate?** | | | |  | | |  |
|  | For Yes, either ONE of the following:  🗷 at least two reviewers independently agreed on selection of eligible studies and achieved consensus on which studies to include   OR two reviewers selected a sample of eligible studies and achieved good agreement (at least 80 percent), with the remainder selected by one reviewer. | | | | 🗷 | | Yes No |  |
|  | Comments: None | | | | | | |  |
| **6. Did the review authors perform data extraction in duplicate?** | | | | | | | | |
| For Yes, either ONE of the following:  🗷 at least two reviewers achieved consensus on which data to extract from included studies   OR two reviewers extracted data from a sample of eligible studies and achieved good agreement (at least 80 percent), with the remainder extracted by one reviewer. | | | | | | 🗷 Yes   No | | |
| Comments: None | | | | | | | | |
| **7. Did the review authors provide a list of excluded studies and justify the exclusions?** | | | | | | | | |
|  | | For Partial Yes:   provided a list of all potentially relevant studies that were read in full-text form but excluded from the review | | For Yes, must also have:   Justified the exclusion from the review of each potentially relevant study | |  Yes   Partial Yes  🗷 No | | |
|  | | Comments: Just provide through the PRISMA flowchart diagram the reasons for study exclusion | | | | | | |
| **8. Did the review authors describe the included studies in adequate detail?** | | | | | | | | |
|  | | For Partial Yes (ALL the following):  🗷 described populations  🗷 described interventions  n/a described comparators  🗷 described outcomes  🗷 described research designs | | For Yes, should also have ALL the following:   described population in detail   described intervention in detail (including doses where relevant)   described comparator in detail (including doses where relevant)   described study’s setting   timeframe for follow-up | |  Yes  🗷 Partial Yes   No | | |
|  | | Comments: Comparator not applicable. | | | | | | |
| **9. Did the review authors use a satisfactory technique for assessing the risk of bias (RoB) in individual studies that were included in the review?** | | | | | | | | |
|  | | **RCTs**  For Partial Yes, must have assessed RoB from  🗷 unconcealed allocation, *and*  🗷 lack of blinding of patients and assessors when assessing outcomes (unnecessary for objective outcomes such as all-cause mortality) | | For Yes, must also have assessed RoB from:  🗷 allocation sequence that was not truly random, *and*  🗷 selection of the reported result from among multiple measurements or analyses of a specified outcome | | 🗷 Yes   Partial Yes   No   Includes only NRSI | | |
|  | | **NRSI**  For Partial Yes, must have assessed RoB:   from confounding, *and*   from selection bias | | For Yes, must also have assessed RoB:   methods used to ascertain exposures and outcomes, *and*   selection of the reported result from among multiple measurements or analyses of a specified | |  Yes   Partial Yes   No 🗷 Includes only RCTs | | |
| Comments: PEDro scale assessment was used. | | | | | | | | |

| **10. Did the review authors report outcomes on the sources of funding for the studies included in the review?** | | | | | | |
| --- | --- | --- | --- | --- | --- | --- |
| For Yes:  🗷 Must have reported on the sources of funding for individual studies included in the review.  Note: Reporting that the reviewers looked for this information but it was not reported by study authors also qualifies. | | | | 🗷 Yes   No |  |  |
| Comments: | | | |  |  |  |
| **11. If meta-analysis was performed did the review authors use appropriate methods for statistical combination of results?** | | | | | | |
|  | **RCTs**  For Yes:  🗷 The authors justified combining the data in a meta-analysis  🗷 AND they used an appropriate weighted technique to combine study results and adjusted for heterogeneity if present.   AND investigated the causes of any heterogeneity | | 🗷 Yes   No   No meta-analysis conducted | | |  |
|  | **For NRSI**  For Yes:   The authors justified combining the data in a meta-analysis   AND they used an appropriate weighted technique to combine study results, adjusting for heterogeneity if present   AND they statistically combined effect estimates from NRSI that were adjusted for confounding, rather than combining raw data, or justified combining raw data when adjusted effect estimates were not available   AND they reported separate summary estimates for RCTs and NRSI separately when both were included in the review | |  Yes   No   No meta-analysis conducted | | |  |
|  | Comments: Meta-analysis was conducted for categorical outcomes. | | | | |  |
| **12. If meta-analysis was performed, did the review authors assess the potential impact of RoB in individual studies on the results of the meta-analysis or other evidence synthesis?** | | | | | | |
|  | For Yes:   included only low risk of bias RCTs   OR, if the pooled estimate was based on RCTs and/or NRSI at variable RoB, the authors performed analyses to investigate possible impact of RoB on summary estimates of effect. | |  Yes  🗷 No   No meta-analysis conducted | | |  |
|  | Comments: None. | | | | |  |
| **13. Did the review authors account for RoB in individual studies when interpreting/ discussing the results of the review?** | | | | | | |
|  | For Yes:   included only low risk of bias RCTs   OR, if RCTs with moderate or high RoB, or NRSI were included the review provided a discussion of the likely impact of RoB on the results | |  Yes  🗷 No | | |  |
|  | Comments: None | | | | |  |
| **14. Did the review authors provide a satisfactory explanation for, and discussion of, any heterogeneity observed in the results of the review?** | | | | | | |
|  | For Yes:   There was no significant heterogeneity in the results  🗷 OR if heterogeneity was present the authors performed an investigation of sources of any heterogeneity in the results and discussed the impact of this on the results of the review | | 🗷 Yes   No | | |  |
|  | Comments: Discussion was provided to evaluate the potential causes of the great heterogeneity observed in the results. | | | | |  |
| **15. If they performed quantitative synthesis did the review authors carry out an adequate investigation of publication bias (small study bias) and discuss its likely impact on the results of the review?** | | | | | | |
|  | For Yes:   performed graphical or statistical tests for publication bias and discussed the likelihood and magnitude of impact of publication bias | |  Yes  🗷 No   No meta-analysis conducted | | |  |
|  | Comments: None. | | | | |  |
| **16. Did the review authors report any potential sources of conflict of interest, including any funding they received for conducting the review?** | | | | | | |
|  | For Yes:  🗷 The authors reported no competing interests OR   The authors described their funding sources and how they managed potential conflicts of interest | 🗷 Yes   No | | | |  |
|  | Comments: None. | | | | |  |

# Study 4 – Cordes et al

| **1. Did the research questions and inclusion criteria for the review include the components of PICO?** | | | | | | | | |
| --- | --- | --- | --- | --- | --- | --- | --- | --- |
| For Yes:  🗷 Population  🗷 Intervention  🗷 Comparator group  🗷 Outcome | | | Optional (recommended)   Timeframe for follow-up | | 🗷 | | Yes No |  |
| Comments: Intervention in this study is associated with screen-to-screen therapy and comparator is the face-to-face therapy. | | | | | | | |  |
| **2. Did the report of the review contain an explicit statement that the review methods were established prior to the conduct of the review and did the report justify any significant deviations from the protocol?** | | | | | | | | |
|  | For Partial Yes:  The authors state that they had a written protocol or guide that included ALL the following:  🗷 review question(s)  🗷 a search strategy  🗷 inclusion/exclusion criteria   a risk of bias assessment | | For Yes:  As for partial yes, plus the protocol should be registered and should also have specified:   a meta-analysis/synthesis plan, if appropriate, *and*   a plan for investigating causes of heterogeneity   justification for any deviations from the protocol | |   🗷 | | Yes  Partial Yes  No |  |
|  | Comments: Protocol was not mentioned within this scoping review. Risk of bias was not performed. | | | | | | |  |
| **3. Did the review authors explain their selection of the study designs for inclusion in the review?** | | | | | | | | |
|  | For Yes, the review should satisfy ONE of the following:   *Explanation for* including only RCTs   OR *Explanation for* including only NRSI  🗷 OR *Explanation for* including both RCTs and NRSI | | | | 🗷 | | Yes No |  |
|  | Comments: Wide variety of study designs included. | | | | | | |  |
| **4. Did the review authors use a comprehensive literature search strategy?** | | | | | | | | |
|  | For Partial Yes (all the following): | | For Yes, should also have (all the following):  🗷 searched the reference lists / bibliographies of included studies   searched trial/study registries   included/consulted content experts in the field   where relevant, searched for grey literature   conducted search within 24 months of completion of the review | |  | |  |  |
|  | 🗷 searched at least 2 databases (relevant to research question)  🗷 provided key word and/or search strategy  🗷 justified publication restrictions | |  |  |   🗷 | | Yes  Partial Yes No |  |
|  | (e.g. language) | |  |  |  | |  |  |
|  | Comments: Additionally to the search in the databases performed, the authors searched the website of the American Speech-Language-Hearing Association, which contains studies for the discipline of speech therapy. | | | | | | |  |
|  | **5. Did the review authors perform study selection in duplicate?** | | | |  | | |  |
|  | For Yes, either ONE of the following:   at least two reviewers independently agreed on selection of eligible studies and achieved consensus on which studies to include   OR two reviewers selected a sample of eligible studies and achieved good agreement (at least 80 percent), with the remainder selected by one reviewer. | | | | 🗷 | | Yes No |  |
|  | Comments: The selection process was not described in the methodology section. | | | | | | |  |
| **6. Did the review authors perform data extraction in duplicate?** | | | | | | | | |
| For Yes, either ONE of the following:   at least two reviewers achieved consensus on which data to extract from included studies   OR two reviewers extracted data from a sample of eligible studies and achieved good agreement (at least 80 percent), with the remainder extracted by one reviewer. | | | | | |  Yes  🗷 No | | |
| Comments: The extraction process was not described in the methodology section. | | | | | | | | |
| **7. Did the review authors provide a list of excluded studies and justify the exclusions?** | | | | | | | | |
|  | | For Partial Yes:   provided a list of all potentially relevant studies that were read in full-text form but excluded from the review | | For Yes, must also have:   Justified the exclusion from the review of each potentially relevant study | |  Yes   Partial Yes  🗷 No | | |
|  | | Comments: The excluded studies are only mentioned in the PRISMA flowchart figure. The authors did not make available the one-by-one reasons for exclusion. | | | | | | |
| **8. Did the review authors describe the included studies in adequate detail?** | | | | | | | | |
|  | | For Partial Yes (ALL the following):  🗷 described populations  🗷 described interventions  🗷 described comparators  🗷 described outcomes  🗷 described research designs | | For Yes, should also have ALL the following:   described population in detail   described intervention in detail (including doses where relevant)   described comparator in detail (including doses where relevant)   described study’s setting   timeframe for follow-up | |  Yes  🗷 Partial Yes   No | | |
|  | | Comments: None. | | | | | | |
| **9. Did the review authors use a satisfactory technique for assessing the risk of bias (RoB) in individual studies that were included in the review?** | | | | | | | | |
|  | | **RCTs**  For Partial Yes, must have assessed RoB from   unconcealed allocation, *and*   lack of blinding of patients and assessors when assessing outcomes (unnecessary for objective outcomes such as all-cause mortality) | | For Yes, must also have assessed RoB from:   allocation sequence that was not truly random, *and*   selection of the reported result from among multiple measurements or analyses of a specified outcome | |  Yes   Partial Yes  🗷 No   Includes only NRSI | | |
|  | | **NRSI**  For Partial Yes, must have assessed RoB:   from confounding, *and*   from selection bias | | For Yes, must also have assessed RoB:   methods used to ascertain exposures and outcomes, *and*   selection of the reported result from among multiple measurements or analyses of a specified | |  Yes   Partial Yes  🗷 No  Includes only RCTs | | |
| Comments: RoB not considered. | | | | | | | | |

| **10. Did the review authors report outcomes on the sources of funding for the studies included in the review?** | | | | | | |
| --- | --- | --- | --- | --- | --- | --- |
| For Yes:   Must have reported on the sources of funding for individual studies included in the review.  Note: Reporting that the reviewers looked for this information but it was not reported by study authors also qualifies. | | | |  Yes  🗷 No |  |  |
| Comments: None | | | |  |  |  |
| **11. If meta-analysis was performed did the review authors use appropriate methods for statistical combination of results?** | | | | | | |
|  | **RCTs**  For Yes:   The authors justified combining the data in a meta-analysis   AND they used an appropriate weighted technique to combine study results and adjusted for heterogeneity if present.   AND investigated the causes of any heterogeneity | |  Yes   No  🗷 No meta-analysis conducted | | |  |
|  | **For NRSI**  For Yes:   The authors justified combining the data in a meta-analysis   AND they used an appropriate weighted technique to combine study results, adjusting for heterogeneity if present   AND they statistically combined effect estimates from NRSI that were adjusted for confounding, rather than combining raw data, or justified combining raw data when adjusted effect estimates were not available   AND they reported separate summary estimates for RCTs and NRSI separately when both were included in the review | |  Yes   No  🗷 No meta-analysis conducted | | |  |
|  | Comments: None | | | | |  |
| **12. If meta-analysis was performed, did the review authors assess the potential impact of RoB in individual studies on the results of the meta-analysis or other evidence synthesis?** | | | | | | |
|  | For Yes:   included only low risk of bias RCTs   OR, if the pooled estimate was based on RCTs and/or NRSI at variable RoB, the authors performed analyses to investigate possible impact of RoB on summary estimates of effect. | |  Yes   No  🗷 No meta-analysis conducted | | |  |
|  | Comments: | | | | |  |
| **13. Did the review authors account for RoB in individual studies when interpreting/ discussing the results of the review?** | | | | | | |
|  | For Yes:   included only low risk of bias RCTs   OR, if RCTs with moderate or high RoB, or NRSI were included the review provided a discussion of the likely impact of RoB on the results | |  Yes  🗷 No | | |  |
|  | Comments: RoB was not considered in this scoping review. | | | | |  |
| **14. Did the review authors provide a satisfactory explanation for, and discussion of, any heterogeneity observed in the results of the review?** | | | | | | |
|  | For Yes:   There was no significant heterogeneity in the results   OR if heterogeneity was present the authors performed an investigation of sources of any heterogeneity in the results and discussed the impact of this on the results of the review | |  Yes  🗷 No | | |  |
|  | Comments: None | | | | |  |
| **15. If they performed quantitative synthesis did the review authors carry out an adequate investigation of publication bias (small study bias) and discuss its likely impact on the results of the review?** | | | | | | |
|  | For Yes:   performed graphical or statistical tests for publication bias and discussed the likelihood and magnitude of impact of publication bias | |  Yes   No  🗷 No meta-analysis conducted | | |  |
|  | Comments: | | | | |  |
| **16. Did the review authors report any potential sources of conflict of interest, including any funding they received for conducting the review?** | | | | | | |
|  | For Yes:  🗷 The authors reported no competing interests OR   The authors described their funding sources and how they managed potential conflicts of interest | 🗷 Yes   No | | | |  |
|  | Comments: None | | | | |  |

# Study 5 – Cruz et al

| **1. Did the research questions and inclusion criteria for the review include the components of PICO?** | | | | | | | | |
| --- | --- | --- | --- | --- | --- | --- | --- | --- |
| For Yes:  🗷 Population  🗷 Intervention  n/a Comparator group  🗷 Outcome | | | Optional (recommended)   Timeframe for follow-up | | 🗷 | | Yes No |  |
| Comments: In this review, there was no specific comparator in between telemonitoring methodologies; therefore, we considered this item as “not applicable”. | | | | | | | |  |
| **2. Did the report of the review contain an explicit statement that the review methods were established prior to the conduct of the review and did the report justify any significant deviations from the protocol?** | | | | | | | | |
|  | For Partial Yes:  The authors state that they had a written protocol or guide that included ALL the following:   review question(s)   a search strategy   inclusion/exclusion criteria   a risk of bias assessment | | For Yes:  As for partial yes, plus the protocol should be registered and should also have specified:   a meta-analysis/synthesis plan, if appropriate, *and*   a plan for investigating causes of heterogeneity   justification for any deviations from the protocol | |   🗷 | | Yes  Partial Yes  No |  |
|  | Comments: Searches were weekly updated until December 2012. There is not any reference to a published study protocol. | | | | | | |  |
| **3. Did the review authors explain their selection of the study designs for inclusion in the review?** | | | | | | | | |
|  | For Yes, the review should satisfy ONE of the following:   *Explanation for* including only RCTs   OR *Explanation for* including only NRSI  🗷 OR *Explanation for* including both RCTs and NRSI | | | | 🗷 | | Yes No |  |
|  | Comments: None | | | | | | |  |
| **4. Did the review authors use a comprehensive literature search strategy?** | | | | | | | | |
|  | For Partial Yes (all the following): | | For Yes, should also have (all the following):  🗷 searched the reference lists / bibliographies of included studies   searched trial/study registries   included/consulted content experts in the field   where relevant, searched for grey literature   conducted search within 24 months of completion of the review | |  | |  |  |
|  | 🗷 searched at least 2 databases (relevant to research question)  🗷 provided key word and/or search strategy  🗷 justified publication restrictions | |  |  |   🗷 | | Yes  Partial Yes No |  |
|  | (e.g. language) | |  |  |  | |  |  |
|  | Comments: Articles restricted to English, Portuguese, and Spanish. | | | | | | |  |
|  | **5. Did the review authors perform study selection in duplicate?** | | | |  | | |  |
|  | For Yes, either ONE of the following:  🗷 at least two reviewers independently agreed on selection of eligible studies and achieved consensus on which studies to include   OR two reviewers selected a sample of eligible studies and achieved good agreement (at least 80 percent), with the remainder selected by one reviewer. | | | | 🗷 | | Yes No |  |
|  | Comments: None | | | | | | |  |
| **6. Did the review authors perform data extraction in duplicate?** | | | | | | | | |
| For Yes, either ONE of the following:  🗷 at least two reviewers achieved consensus on which data to extract from included studies   OR two reviewers extracted data from a sample of eligible studies and achieved good agreement (at least 80 percent), with the remainder extracted by one reviewer. | | | | | | 🗷 Yes   No | | |
| Comments: In the quality assessment section, the authors stated the overall level of inter-rater agreement (k = 0.78). However, for the entire data extraction phase, there is any referral of this inter-rater agreement. | | | | | | | | |
| **7. Did the review authors provide a list of excluded studies and justify the exclusions?** | | | | | | | | |
|  | | For Partial Yes:   provided a list of all potentially relevant studies that were read in full-text form but excluded from the review | | For Yes, must also have:   Justified the exclusion from the review of each potentially relevant study | |  Yes   Partial Yes  🗷 No | | |
|  | | Comments: Just mentioned the overall reasons for study exclusion in the PRISMA flowchart. | | | | | | |
| **8. Did the review authors describe the included studies in adequate detail?** | | | | | | | | |
|  | | For Partial Yes (ALL the following):  🗷 described populations  🗷 described interventions  n/a described comparators  🗷 described outcomes  🗷 described research designs | | For Yes, should also have ALL the following:  🗷 described population in detail  🗷 described intervention in detail (including doses where relevant)  n/a described comparator in detail (including doses where relevant)  🗷 described study’s setting  🗷 timeframe for follow-up | | 🗷 Yes   Partial Yes   No | | |
|  | | Comments: Studies properly described in Table 1. | | | | | | |
| **9. Did the review authors use a satisfactory technique for assessing the risk of bias (RoB) in individual studies that were included in the review?** | | | | | | | | |
|  | | **RCTs**  For Partial Yes, must have assessed RoB from   unconcealed allocation, *and*   lack of blinding of patients and assessors when assessing outcomes (unnecessary for objective outcomes such as all-cause mortality) | | For Yes, must also have assessed RoB from:   allocation sequence that was not truly random, *and*   selection of the reported result from among multiple measurements or analyses of a specified outcome | |  Yes   Partial Yes  🗷 No   Includes only NRSI | | |
|  | | **NRSI**  For Partial Yes, must have assessed RoB:   from confounding, *and*   from selection bias | | For Yes, must also have assessed RoB:   methods used to ascertain exposures and outcomes, *and*   selection of the reported result from among multiple measurements or analyses of a specified | |  Yes   Partial Yes  🗷 No  Includes only RCTs | | |
| Comments: The authors considered the same approach as Hailey el al. used, which is a vague and confused methodology for assessing study quality and implications for decision making. Therefore, as this study did not used well-known techniques for risk of bias appraisal, we considered insufficient the methodology implemented in this review. | | | | | | | | |

| **10. Did the review authors report outcomes on the sources of funding for the studies included in the review?** | | | | | | |
| --- | --- | --- | --- | --- | --- | --- |
| For Yes:   Must have reported on the sources of funding for individual studies included in the review.  Note: Reporting that the reviewers looked for this information but it was not reported by study authors also qualifies. | | | |  Yes  🗷 No |  |  |
| Comments: None | | | |  |  |  |
| **11. If meta-analysis was performed did the review authors use appropriate methods for statistical combination of results?** | | | | | | |
|  | **RCTs**  For Yes:   The authors justified combining the data in a meta-analysis   AND they used an appropriate weighted technique to combine study results and adjusted for heterogeneity if present.   AND investigated the causes of any heterogeneity | |  Yes   No  🗷 No meta-analysis conducted | | |  |
|  | **For NRSI**  For Yes:   The authors justified combining the data in a meta-analysis   AND they used an appropriate weighted technique to combine study results, adjusting for heterogeneity if present   AND they statistically combined effect estimates from NRSI that were adjusted for confounding, rather than combining raw data, or justified combining raw data when adjusted effect estimates were not available   AND they reported separate summary estimates for RCTs and NRSI separately when both were included in the review | |  Yes   No  🗷 No meta-analysis conducted | | |  |
|  | Comments: None | | | | |  |
| **12. If meta-analysis was performed, did the review authors assess the potential impact of RoB in individual studies on the results of the meta-analysis or other evidence synthesis?** | | | | | | |
|  | For Yes:   included only low risk of bias RCTs   OR, if the pooled estimate was based on RCTs and/or NRSI at variable RoB, the authors performed analyses to investigate possible impact of RoB on summary estimates of effect. | |  Yes   No  🗷 No meta-analysis conducted | | |  |
|  | Comments: None | | | | |  |
| **13. Did the review authors account for RoB in individual studies when interpreting/ discussing the results of the review?** | | | | | | |
|  | For Yes:   included only low risk of bias RCTs   OR, if RCTs with moderate or high RoB, or NRSI were included the review provided a discussion of the likely impact of RoB on the results | |  Yes  🗷 No | | |  |
|  | Comments: None | | | | |  |
| **14. Did the review authors provide a satisfactory explanation for, and discussion of, any heterogeneity observed in the results of the review?** | | | | | | |
|  | For Yes:   There was no significant heterogeneity in the results   OR if heterogeneity was present the authors performed an investigation of sources of any heterogeneity in the results and discussed the impact of this on the results of the review | |  Yes  🗷 No | | |  |
|  | Comments: None | | | | |  |
| **15. If they performed quantitative synthesis did the review authors carry out an adequate investigation of publication bias (small study bias) and discuss its likely impact on the results of the review?** | | | | | | |
|  | For Yes:   performed graphical or statistical tests for publication bias and discussed the likelihood and magnitude of impact of publication bias | |  Yes   No  🗷 No meta-analysis conducted | | |  |
|  | Comments: None | | | | |  |
| **16. Did the review authors report any potential sources of conflict of interest, including any funding they received for conducting the review?** | | | | | | |
|  | For Yes:  🗷 The authors reported no competing interests OR   The authors described their funding sources and how they managed potential conflicts of interest | 🗷 Yes   No | | | |  |
|  | Comments: None | | | | |  |

# Study 6 – Elbaz et al

| **1. Did the research questions and inclusion criteria for the review include the components of PICO?** | | | | | | | | |
| --- | --- | --- | --- | --- | --- | --- | --- | --- |
| For Yes:  🗷 Population  🗷 Intervention  🗷 Comparator group  🗷 Outcome | | | Optional (recommended)   Timeframe for follow-up | | 🗷 | | Yes No |  |
| Comments: Not all included studies had a control/comparator group. However, in general, the study considered different arms composition as an eligible study. | | | | | | | |  |
| **2. Did the report of the review contain an explicit statement that the review methods were established prior to the conduct of the review and did the report justify any significant deviations from the protocol?** | | | | | | | | |
|  | For Partial Yes:  The authors state that they had a written protocol or guide that included ALL the following:   review question(s)   a search strategy   inclusion/exclusion criteria   a risk of bias assessment | | For Yes:  As for partial yes, plus the protocol should be registered and should also have specified:   a meta-analysis/synthesis plan, if appropriate, *and*   a plan for investigating causes of heterogeneity   justification for any deviations from the protocol | |   🗷 | | Yes  Partial Yes  No |  |
|  | Comments: There is not any reference to a published study protocol. | | | | | | |  |
| **3. Did the review authors explain their selection of the study designs for inclusion in the review?** | | | | | | | | |
|  | For Yes, the review should satisfy ONE of the following:   *Explanation for* including only RCTs   OR *Explanation for* including only NRSI  🗷 OR *Explanation for* including both RCTs and NRSI | | | | 🗷 | | Yes No |  |
|  | Comments: | | | | | | |  |
| **4. Did the review authors use a comprehensive literature search strategy?** | | | | | | | | |
|  | For Partial Yes (all the following): | | For Yes, should also have (all the following):  🗷 searched the reference lists / bibliographies of included studies   searched trial/study registries   included/consulted content experts in the field   where relevant, searched for grey literature   conducted search within 24 months of completion of the review | |  | |  |  |
|  | 🗷 searched at least 2 databases (relevant to research question)  🗷 provided key word and/or search strategy  🗷 justified publication restrictions | |  |  |   🗷 | | Yes  Partial Yes No |  |
|  | (e.g. language) | |  |  |  | |  |  |
|  | Comments: Only studies in English. | | | | | | |  |
|  | **5. Did the review authors perform study selection in duplicate?** | | | |  | | |  |
|  | For Yes, either ONE of the following:  🗷 at least two reviewers independently agreed on selection of eligible studies and achieved consensus on which studies to include   OR two reviewers selected a sample of eligible studies and achieved good agreement (at least 80 percent), with the remainder selected by one reviewer. | | | | 🗷 | | Yes No |  |
|  | Comments: None | | | | | | |  |
| **6. Did the review authors perform data extraction in duplicate?** | | | | | | | | |
| For Yes, either ONE of the following:   at least two reviewers achieved consensus on which data to extract from included studies   OR two reviewers extracted data from a sample of eligible studies and achieved good agreement (at least 80 percent), with the remainder extracted by one reviewer. | | | | | |  Yes  🗷 No | | |
| Comments: Extraction phase is not mentioned throughout the methodology section. | | | | | | | | |
| **7. Did the review authors provide a list of excluded studies and justify the exclusions?** | | | | | | | | |
|  | | For Partial Yes:   provided a list of all potentially relevant studies that were read in full-text form but excluded from the review | | For Yes, must also have:   Justified the exclusion from the review of each potentially relevant study | |  Yes   Partial Yes  🗷 No | | |
|  | | Comments: Just mentioned the overall reasons for study exclusion in the PRISMA flowchart. | | | | | | |
| **8. Did the review authors describe the included studies in adequate detail?** | | | | | | | | |
|  | | For Partial Yes (ALL the following):  🗷 described populations  🗷 described interventions  🗷 described comparators  🗷 described outcomes  🗷 described research designs | | For Yes, should also have ALL the following:  🗷 described population in detail  🗷 described intervention in detail (including doses where relevant)  🗷 described comparator in detail (including doses where relevant)  🗷 described study’s setting   timeframe for follow-up | |  Yes  🗷 Partial Yes   No | | |
|  | | Comments: Follow-up period not described for any of included studies. | | | | | | |
| **9. Did the review authors use a satisfactory technique for assessing the risk of bias (RoB) in individual studies that were included in the review?** | | | | | | | | |
|  | | **RCTs**  For Partial Yes, must have assessed RoB from   unconcealed allocation, *and*   lack of blinding of patients and assessors when assessing outcomes (unnecessary for objective outcomes such as all-cause mortality) | | For Yes, must also have assessed RoB from:   allocation sequence that was not truly random, *and*   selection of the reported result from among multiple measurements or analyses of a specified outcome | |  Yes   Partial Yes  🗷 No   Includes only NRSI | | |
|  | | **NRSI**  For Partial Yes, must have assessed RoB:   from confounding, *and*   from selection bias | | For Yes, must also have assessed RoB:   methods used to ascertain exposures and outcomes, *and*   selection of the reported result from among multiple measurements or analyses of a specified | |  Yes   Partial Yes  🗷 No  Includes only RCTs | | |
| Comments: No RoB tool was mentioned in the manuscript text. | | | | | | | | |

| **10. Did the review authors report outcomes on the sources of funding for the studies included in the review?** | | | | | | |
| --- | --- | --- | --- | --- | --- | --- |
| For Yes:   Must have reported on the sources of funding for individual studies included in the review.  Note: Reporting that the reviewers looked for this information but it was not reported by study authors also qualifies. | | | |  Yes  🗷 No |  |  |
| Comments: None | | | |  |  |  |
| **11. If meta-analysis was performed did the review authors use appropriate methods for statistical combination of results?** | | | | | | |
|  | **RCTs**  For Yes:   The authors justified combining the data in a meta-analysis   AND they used an appropriate weighted technique to combine study results and adjusted for heterogeneity if present.   AND investigated the causes of any heterogeneity | |  Yes   No  🗷 No meta-analysis conducted | | |  |
|  | **For NRSI**  For Yes:   The authors justified combining the data in a meta-analysis   AND they used an appropriate weighted technique to combine study results, adjusting for heterogeneity if present   AND they statistically combined effect estimates from NRSI that were adjusted for confounding, rather than combining raw data, or justified combining raw data when adjusted effect estimates were not available   AND they reported separate summary estimates for RCTs and NRSI separately when both were included in the review | |  Yes   No  🗷 No meta-analysis conducted | | |  |
|  | Comments: None | | | | |  |
| **12. If meta-analysis was performed, did the review authors assess the potential impact of RoB in individual studies on the results of the meta-analysis or other evidence synthesis?** | | | | | | |
|  | For Yes:   included only low risk of bias RCTs   OR, if the pooled estimate was based on RCTs and/or NRSI at variable RoB, the authors performed analyses to investigate possible impact of RoB on summary estimates of effect. | |  Yes   No  🗷 No meta-analysis conducted | | |  |
|  | Comments: None | | | | |  |
| **13. Did the review authors account for RoB in individual studies when interpreting/ discussing the results of the review?** | | | | | | |
|  | For Yes:   included only low risk of bias RCTs   OR, if RCTs with moderate or high RoB, or NRSI were included the review provided a discussion of the likely impact of RoB on the results | |  Yes  🗷 No | | |  |
|  | Comments: None | | | | |  |
| **14. Did the review authors provide a satisfactory explanation for, and discussion of, any heterogeneity observed in the results of the review?** | | | | | | |
|  | For Yes:   There was no significant heterogeneity in the results   OR if heterogeneity was present the authors performed an investigation of sources of any heterogeneity in the results and discussed the impact of this on the results of the review | |  Yes  🗷 No | | |  |
|  | Comments: None | | | | |  |
| **15. If they performed quantitative synthesis did the review authors carry out an adequate investigation of publication bias (small study bias) and discuss its likely impact on the results of the review?** | | | | | | |
|  | For Yes:   performed graphical or statistical tests for publication bias and discussed the likelihood and magnitude of impact of publication bias | |  Yes   No  🗷 No meta-analysis conducted | | |  |
|  | Comments: None | | | | |  |
| **16. Did the review authors report any potential sources of conflict of interest, including any funding they received for conducting the review?** | | | | | | |
|  | For Yes:  🗷 The authors reported no competing interests OR   The authors described their funding sources and how they managed potential conflicts of interest | 🗷 Yes   No | | | |  |
|  | Comments: None. | | | | |  |

# Study 7 – Farabi et al

| **1. Did the research questions and inclusion criteria for the review include the components of PICO?** | | | | | | | | |
| --- | --- | --- | --- | --- | --- | --- | --- | --- |
| For Yes:  🗷 Population  🗷 Intervention  🗷 Comparator group  🗷 Outcome | | | Optional (recommended)   Timeframe for follow-up | | 🗷 | | Yes No |  |
| Comments: The authors have stated that they had primarily focused on the PICO elements for the study preparation and execution. In addition, in Table 2, they provided a comprehensive description of all alternative options for comparison to the telemedicine tools. | | | | | | | |  |
| **2. Did the report of the review contain an explicit statement that the review methods were established prior to the conduct of the review and did the report justify any significant deviations from the protocol?** | | | | | | | | |
|  | For Partial Yes:  The authors state that they had a written protocol or guide that included ALL the following:   review question(s)   a search strategy   inclusion/exclusion criteria   a risk of bias assessment | | For Yes:  As for partial yes, plus the protocol should be registered and should also have specified:   a meta-analysis/synthesis plan, if appropriate, *and*   a plan for investigating causes of heterogeneity   justification for any deviations from the protocol | |   🗷 | | Yes  Partial Yes  No |  |
|  | Comments: There is not any reference to a published study protocol. | | | | | | |  |
| **3. Did the review authors explain their selection of the study designs for inclusion in the review?** | | | | | | | | |
|  | For Yes, the review should satisfy ONE of the following:   *Explanation for* including only RCTs   OR *Explanation for* including only NRSI  🗷 OR *Explanation for* including both RCTs and NRSI | | | | 🗷 | | Yes No |  |
|  | Comments: None | | | | | | |  |
| **4. Did the review authors use a comprehensive literature search strategy?** | | | | | | | | |
|  | For Partial Yes (all the following): | | For Yes, should also have (all the following):  🗷 searched the reference lists / bibliographies of included studies   searched trial/study registries   included/consulted content experts in the field  🗷 where relevant, searched for grey literature   conducted search within 24 months of completion of the review | |  | |  |  |
|  | 🗷 searched at least 2 databases (relevant to research question)  🗷 provided key word and/or search strategy  🗷 justified publication restrictions | |  |  |   🗷 | | Yes  Partial Yes No |  |
|  | (e.g. language) | |  |  |  | |  |  |
|  | Comments: Only articles in English and Persian. | | | | | | |  |
|  | **5. Did the review authors perform study selection in duplicate?** | | | |  | | |  |
|  | For Yes, either ONE of the following:  🗷 at least two reviewers independently agreed on selection of eligible studies and achieved consensus on which studies to include   OR two reviewers selected a sample of eligible studies and achieved good agreement (at least 80 percent), with the remainder selected by one reviewer. | | | | 🗷 | | Yes No |  |
|  | Comments: None | | | | | | |  |
| **6. Did the review authors perform data extraction in duplicate?** | | | | | | | | |
| For Yes, either ONE of the following:  🗷 at least two reviewers achieved consensus on which data to extract from included studies   OR two reviewers extracted data from a sample of eligible studies and achieved good agreement (at least 80 percent), with the remainder extracted by one reviewer. | | | | | | 🗷 Yes   No | | |
| Comments: None | | | | | | | | |
| **7. Did the review authors provide a list of excluded studies and justify the exclusions?** | | | | | | | | |
|  | | For Partial Yes:   provided a list of all potentially relevant studies that were read in full-text form but excluded from the review | | For Yes, must also have:   Justified the exclusion from the review of each potentially relevant study | |  Yes   Partial Yes  🗷 No | | |
|  | | Comments: Not even described/ mentioned the overall reasons for study exclusion in the PRISMA flowchart. | | | | | | |
| **8. Did the review authors describe the included studies in adequate detail?** | | | | | | | | |
|  | | For Partial Yes (ALL the following):  🗷 described populations  🗷 described interventions  🗷 described comparators  🗷 described outcomes  🗷 described research designs | | For Yes, should also have ALL the following:  🗷 described population in detail  🗷 described intervention in detail (including doses where relevant)  🗷 described comparator in detail (including doses where relevant)  🗷 described study’s setting   timeframe for follow-up | |  Yes  🗷 Partial Yes   No | | |
|  | | Comments: None | | | | | | |
| **9. Did the review authors use a satisfactory technique for assessing the risk of bias (RoB) in individual studies that were included in the review?** | | | | | | | | |
|  | | **RCTs**  For Partial Yes, must have assessed RoB from   unconcealed allocation, *and*   lack of blinding of patients and assessors when assessing outcomes (unnecessary for objective outcomes such as all-cause mortality) | | For Yes, must also have assessed RoB from:   allocation sequence that was not truly random, *and*   selection of the reported result from among multiple measurements or analyses of a specified outcome | | 🗷 Yes   Partial Yes   No   Includes only NRSI | | |
|  | | **NRSI**  For Partial Yes, must have assessed RoB:   from confounding, *and*   from selection bias | | For Yes, must also have assessed RoB:   methods used to ascertain exposures and outcomes, *and*   selection of the reported result from among multiple measurements or analyses of a specified | | 🗷 Yes   Partial Yes   No  Includes only RCTs | | |
| Comments: Indeed, this is not a typical systematic review of interventions. This review focuses on an economic evaluation of telemedicine tools in patients diagnosed with cardiovascular diseases. For that, this review uses a specific quality assessment methodology (Consolidated Health Economic Evaluation Reporting Standards). Therefore, we considered appropriate the evaluation presented, even though it is not essentially foreseen in this categorical quality assessment protocol. | | | | | | | | |

| **10. Did the review authors report outcomes on the sources of funding for the studies included in the review?** | | | | | | |
| --- | --- | --- | --- | --- | --- | --- |
| For Yes:   Must have reported on the sources of funding for individual studies included in the review.  Note: Reporting that the reviewers looked for this information but it was not reported by study authors also qualifies. | | | |  Yes  🗷 No |  |  |
| Comments: None | | | |  |  |  |
| **11. If meta-analysis was performed did the review authors use appropriate methods for statistical combination of results?** | | | | | | |
|  | **RCTs**  For Yes:   The authors justified combining the data in a meta-analysis   AND they used an appropriate weighted technique to combine study results and adjusted for heterogeneity if present.   AND investigated the causes of any heterogeneity | |  Yes   No  🗷 No meta-analysis conducted | | |  |
|  | **For NRSI**  For Yes:   The authors justified combining the data in a meta-analysis   AND they used an appropriate weighted technique to combine study results, adjusting for heterogeneity if present   AND they statistically combined effect estimates from NRSI that were adjusted for confounding, rather than combining raw data, or justified combining raw data when adjusted effect estimates were not available   AND they reported separate summary estimates for RCTs and NRSI separately when both were included in the review | |  Yes   No  🗷 No meta-analysis conducted | | |  |
|  | Comments: None | | | | |  |
| **12. If meta-analysis was performed, did the review authors assess the potential impact of RoB in individual studies on the results of the meta-analysis or other evidence synthesis?** | | | | | | |
|  | For Yes:   included only low risk of bias RCTs   OR, if the pooled estimate was based on RCTs and/or NRSI at variable RoB, the authors performed analyses to investigate possible impact of RoB on summary estimates of effect. | |  Yes   No  🗷 No meta-analysis conducted | | |  |
|  | Comments: None | | | | |  |
| **13. Did the review authors account for RoB in individual studies when interpreting/ discussing the results of the review?** | | | | | | |
|  | For Yes:   included only low risk of bias RCTs  🗷 OR, if RCTs with moderate or high RoB, or NRSI were included the review provided a discussion of the likely impact of RoB on the results | | 🗷 Yes   No | | |  |
|  | Comments: None | | | | |  |
| **14. Did the review authors provide a satisfactory explanation for, and discussion of, any heterogeneity observed in the results of the review?** | | | | | | |
|  | For Yes:   There was no significant heterogeneity in the results  🗷 OR if heterogeneity was present the authors performed an investigation of sources of any heterogeneity in the results and discussed the impact of this on the results of the review | | 🗷 Yes   No | | |  |
|  | Comments: None | | | | |  |
| **15. If they performed quantitative synthesis did the review authors carry out an adequate investigation of publication bias (small study bias) and discuss its likely impact on the results of the review?** | | | | | | |
|  | For Yes:   performed graphical or statistical tests for publication bias and discussed the likelihood and magnitude of impact of publication bias | |  Yes   No  🗷 No meta-analysis conducted | | |  |
|  | Comments: None | | | | |  |
| **16. Did the review authors report any potential sources of conflict of interest, including any funding they received for conducting the review?** | | | | | | |
|  | For Yes:  🗷 The authors reported no competing interests OR   The authors described their funding sources and how they managed potential conflicts of interest | 🗷 Yes   No | | | |  |
|  | Comments: None | | | | |  |

# Study 8 – Gaveikate et al

| **1. Did the research questions and inclusion criteria for the review include the components of PICO?** | | | | | | | | |
| --- | --- | --- | --- | --- | --- | --- | --- | --- |
| For Yes:  🗷 Population  🗷 Intervention  🗷 Comparator group  🗷 Outcome | | | Optional (recommended)   Timeframe for follow-up | | 🗷 | | Yes No |  |
| Comments: | | | | | | | |  |
| **2. Did the report of the review contain an explicit statement that the review methods were established prior to the conduct of the review and did the report justify any significant deviations from the protocol?** | | | | | | | | |
|  | For Partial Yes:  The authors state that they had a written protocol or guide that included ALL the following:   review question(s)   a search strategy   inclusion/exclusion criteria   a risk of bias assessment | | For Yes:  As for partial yes, plus the protocol should be registered and should also have specified:   a meta-analysis/synthesis plan, if appropriate, *and*   a plan for investigating causes of heterogeneity   justification for any deviations from the protocol | |   🗷 | | Yes  Partial Yes  No |  |
|  | Comments: There is not any reference to a published study protocol. | | | | | | |  |
| **3. Did the review authors explain their selection of the study designs for inclusion in the review?** | | | | | | | | |
|  | For Yes, the review should satisfy ONE of the following:   *Explanation for* including only RCTs   OR *Explanation for* including only NRSI  🗷 OR *Explanation for* including both RCTs and NRSI | | | | 🗷 | | Yes No |  |
|  | Comments: This is an overview of systematic reviews, which in a later stage evaluated primary studies. | | | | | | |  |
| **4. Did the review authors use a comprehensive literature search strategy?** | | | | | | | | |
|  | For Partial Yes (all the following): | | For Yes, should also have (all the following):  🗷 searched the reference lists / bibliographies of included studies   searched trial/study registries   included/consulted content experts in the field   where relevant, searched for grey literature   conducted search within 24 months of completion of the review | |  | |  |  |
|  | 🗷 searched at least 2 databases (relevant to research question)  🗷 provided key word and/or search strategy  🗷 justified publication restrictions | |  |  |   🗷 | | Yes  Partial Yes No |  |
|  | (e.g. language) | |  |  |  | |  |  |
|  | Comments: None | | | | | | |  |
|  | **5. Did the review authors perform study selection in duplicate?** | | | |  | | |  |
|  | For Yes, either ONE of the following:  🗷 at least two reviewers independently agreed on selection of eligible studies and achieved consensus on which studies to include   OR two reviewers selected a sample of eligible studies and achieved good agreement (at least 80 percent), with the remainder selected by one reviewer. | | | | 🗷 | | Yes No |  |
|  | Comments: None | | | | | | |  |
| **6. Did the review authors perform data extraction in duplicate?** | | | | | | | | |
| For Yes, either ONE of the following:  🗷 at least two reviewers achieved consensus on which data to extract from included studies   OR two reviewers extracted data from a sample of eligible studies and achieved good agreement (at least 80 percent), with the remainder extracted by one reviewer. | | | | | | 🗷 Yes   No | | |
| Comments: None | | | | | | | | |
| **7. Did the review authors provide a list of excluded studies and justify the exclusions?** | | | | | | | | |
|  | | For Partial Yes:   provided a list of all potentially relevant studies that were read in full-text form but excluded from the review | | For Yes, must also have:   Justified the exclusion from the review of each potentially relevant study | |  Yes   Partial Yes  🗷 No | | |
|  | | Comments: There is only a description of the overall reasons for study exclusion in the PRISMA flowchart. | | | | | | |
| **8. Did the review authors describe the included studies in adequate detail?** | | | | | | | | |
|  | | For Partial Yes (ALL the following):  🗷 described populations  🗷 described interventions  🗷 described comparators  🗷 described outcomes  🗷 described research designs | | For Yes, should also have ALL the following:  🗷 described population in detail  🗷 described intervention in detail (including doses where relevant)  🗷 described comparator in detail (including doses where relevant)  🗷 described study’s setting  🗷 timeframe for follow-up | | 🗷 Yes   Partial Yes   No | | |
|  | | Comments: None | | | | | | |
| **9. Did the review authors use a satisfactory technique for assessing the risk of bias (RoB) in individual studies that were included in the review?** | | | | | | | | |
|  | | **RCTs**  For Partial Yes, must have assessed RoB from   unconcealed allocation, *and*   lack of blinding of patients and assessors when assessing outcomes (unnecessary for objective outcomes such as all-cause mortality) | | For Yes, must also have assessed RoB from:   allocation sequence that was not truly random, *and*   selection of the reported result from among multiple measurements or analyses of a specified outcome | | 🗷 Yes   Partial Yes   No   Includes only NRSI | | |
|  | | **NRSI**  For Partial Yes, must have assessed RoB:   from confounding, *and*   from selection bias | | For Yes, must also have assessed RoB:   methods used to ascertain exposures and outcomes, *and*   selection of the reported result from among multiple measurements or analyses of a specified | | 🗷 Yes   Partial Yes   No  Includes only RCTs | | |
| Comments: Quality assessment is based on the Appendix Table 2. Therefore, we considered adequate the overall methodological appraisal in the review, even though is not the one primary focused in this tool. | | | | | | | | |

| **10. Did the review authors report outcomes on the sources of funding for the studies included in the review?** | | | | | | |
| --- | --- | --- | --- | --- | --- | --- |
| For Yes:   Must have reported on the sources of funding for individual studies included in the review.  Note: Reporting that the reviewers looked for this information but it was not reported by study authors also qualifies. | | | |  Yes  🗷 No |  |  |
| Comments: None | | | |  |  |  |
| **11. If meta-analysis was performed did the review authors use appropriate methods for statistical combination of results?** | | | | | | |
|  | **RCTs**  For Yes:   The authors justified combining the data in a meta-analysis   AND they used an appropriate weighted technique to combine study results and adjusted for heterogeneity if present.   AND investigated the causes of any heterogeneity | |  Yes   No  🗷 No meta-analysis conducted | | |  |
|  | **For NRSI**  For Yes:   The authors justified combining the data in a meta-analysis   AND they used an appropriate weighted technique to combine study results, adjusting for heterogeneity if present   AND they statistically combined effect estimates from NRSI that were adjusted for confounding, rather than combining raw data, or justified combining raw data when adjusted effect estimates were not available   AND they reported separate summary estimates for RCTs and NRSI separately when both were included in the review | |  Yes   No  🗷 No meta-analysis conducted | | |  |
|  | Comments: None | | | | |  |
| **12. If meta-analysis was performed, did the review authors assess the potential impact of RoB in individual studies on the results of the meta-analysis or other evidence synthesis?** | | | | | | |
|  | For Yes:   included only low risk of bias RCTs   OR, if the pooled estimate was based on RCTs and/or NRSI at variable RoB, the authors performed analyses to investigate possible impact of RoB on summary estimates of effect. | |  Yes   No  🗷 No meta-analysis conducted | | |  |
|  | Comments: None | | | | |  |
| **13. Did the review authors account for RoB in individual studies when interpreting/ discussing the results of the review?** | | | | | | |
|  | For Yes:   included only low risk of bias RCTs   OR, if RCTs with moderate or high RoB, or NRSI were included the review provided a discussion of the likely impact of RoB on the results | | 🗷 Yes   No | | |  |
|  | Comments: None. | | | | |  |
| **14. Did the review authors provide a satisfactory explanation for, and discussion of, any heterogeneity observed in the results of the review?** | | | | | | |
|  | For Yes:   There was no significant heterogeneity in the results   OR if heterogeneity was present the authors performed an investigation of sources of any heterogeneity in the results and discussed the impact of this on the results of the review | | 🗷 Yes   No | | |  |
|  | Comments: None | | | | |  |
| **15. If they performed quantitative synthesis did the review authors carry out an adequate investigation of publication bias (small study bias) and discuss its likely impact on the results of the review?** | | | | | | |
|  | For Yes:   performed graphical or statistical tests for publication bias and discussed the likelihood and magnitude of impact of publication bias | |  Yes   No  🗷 No meta-analysis conducted | | |  |
|  | Comments: None | | | | |  |
| **16. Did the review authors report any potential sources of conflict of interest, including any funding they received for conducting the review?** | | | | | | |
|  | For Yes:  🗷 The authors reported no competing interests OR   The authors described their funding sources and how they managed potential conflicts of interest | 🗷 Yes   No | | | |  |
|  | Comments: None | | | | |  |

# Study 9 – Glinkowski et al

| **1. Did the research questions and inclusion criteria for the review include the components of PICO?** | | | | | | | | |
| --- | --- | --- | --- | --- | --- | --- | --- | --- |
| For Yes:  🗷 Population  🗷 Intervention  n/a Comparator group  🗷 Outcome | | | Optional (recommended)   Timeframe for follow-up | | 🗷 | | Yes No |  |
| Comments: A comparator was not prioritized in this study. | | | | | | | |  |
| **2. Did the report of the review contain an explicit statement that the review methods were established prior to the conduct of the review and did the report justify any significant deviations from the protocol?** | | | | | | | | |
|  | For Partial Yes:  The authors state that they had a written protocol or guide that included ALL the following:   review question(s)   a search strategy   inclusion/exclusion criteria   a risk of bias assessment | | For Yes:  As for partial yes, plus the protocol should be registered and should also have specified:   a meta-analysis/synthesis plan, if appropriate, *and*   a plan for investigating causes of heterogeneity   justification for any deviations from the protocol | |   🗷 | | Yes  Partial Yes  No |  |
|  | Comments: There is not any reference to a published study protocol. | | | | | | |  |
| **3. Did the review authors explain their selection of the study designs for inclusion in the review?** | | | | | | | | |
|  | For Yes, the review should satisfy ONE of the following:   *Explanation for* including only RCTs   OR *Explanation for* including only NRSI  🗷 OR *Explanation for* including both RCTs and NRSI | | | | 🗷 | | Yes No |  |
|  | Comments: None | | | | | | |  |
| **4. Did the review authors use a comprehensive literature search strategy?** | | | | | | | | |
|  | For Partial Yes (all the following): | | For Yes, should also have (all the following):   searched the reference lists / bibliographies of included studies   searched trial/study registries   included/consulted content experts in the field  🗷 where relevant, searched for grey literature   conducted search within 24 months of completion of the review | |  | |  |  |
|  | 🗷 searched at least 2 databases (relevant to research question)  🗷 provided key word and/or search strategy  🗷 justified publication restrictions | |  |  |   🗷 | | Yes  Partial Yes No |  |
|  | (e.g. language) | |  |  |  | |  |  |
|  | Comments: None | | | | | | |  |
|  | **5. Did the review authors perform study selection in duplicate?** | | | |  | | |  |
|  | For Yes, either ONE of the following:  🗷 at least two reviewers independently agreed on selection of eligible studies and achieved consensus on which studies to include   OR two reviewers selected a sample of eligible studies and achieved good agreement (at least 80 percent), with the remainder selected by one reviewer. | | | | 🗷 | | Yes No |  |
|  | Comments: None | | | | | | |  |
| **6. Did the review authors perform data extraction in duplicate?** | | | | | | | | |
| For Yes, either ONE of the following:  🗷 at least two reviewers achieved consensus on which data to extract from included studies   OR two reviewers extracted data from a sample of eligible studies and achieved good agreement (at least 80 percent), with the remainder extracted by one reviewer. | | | | | | 🗷 Yes   No | | |
| Comments: None | | | | | | | | |
| **7. Did the review authors provide a list of excluded studies and justify the exclusions?** | | | | | | | | |
|  | | For Partial Yes:   provided a list of all potentially relevant studies that were read in full-text form but excluded from the review | | For Yes, must also have:   Justified the exclusion from the review of each potentially relevant study | |  Yes   Partial Yes  🗷 No | | |
|  | | Comments: There is only a description of the overall reasons for study exclusion in the PRISMA flowchart. | | | | | | |
| **8. Did the review authors describe the included studies in adequate detail?** | | | | | | | | |
|  | | For Partial Yes (ALL the following):   described populations   described interventions   described comparators   described outcomes   described research designs | | For Yes, should also have ALL the following:   described population in detail   described intervention in detail (including doses where relevant)   described comparator in detail (including doses where relevant)   described study’s setting   timeframe for follow-up | |  Yes   Partial Yes  🗷 No | | |
|  | | Comments: No description of included studies is given whatsoever. | | | | | | |
| **9. Did the review authors use a satisfactory technique for assessing the risk of bias (RoB) in individual studies that were included in the review?** | | | | | | | | |
|  | | **RCTs**  For Partial Yes, must have assessed RoB from   unconcealed allocation, *and*   lack of blinding of patients and assessors when assessing outcomes (unnecessary for objective outcomes such as all-cause mortality) | | For Yes, must also have assessed RoB from:   allocation sequence that was not truly random, *and*   selection of the reported result from among multiple measurements or analyses of a specified outcome | |  Yes   Partial Yes  🗷 No   Includes only NRSI | | |
|  | | **NRSI**  For Partial Yes, must have assessed RoB:   from confounding, *and*   from selection bias | | For Yes, must also have assessed RoB:   methods used to ascertain exposures and outcomes, *and*   selection of the reported result from among multiple measurements or analyses of a specified | |  Yes   Partial Yes  🗷 No  Includes only RCTs | | |
| Comments: None | | | | | | | | |

| **10. Did the review authors report outcomes on the sources of funding for the studies included in the review?** | | | | | | |
| --- | --- | --- | --- | --- | --- | --- |
| For Yes:   Must have reported on the sources of funding for individual studies included in the review.  Note: Reporting that the reviewers looked for this information but it was not reported by study authors also qualifies. | | | |  Yes  🗷 No |  |  |
| Comments: None | | | |  |  |  |
| **11. If meta-analysis was performed did the review authors use appropriate methods for statistical combination of results?** | | | | | | |
|  | **RCTs**  For Yes:   The authors justified combining the data in a meta-analysis   AND they used an appropriate weighted technique to combine study results and adjusted for heterogeneity if present.   AND investigated the causes of any heterogeneity | |  Yes   No  🗷 No meta-analysis conducted | | |  |
|  | **For NRSI**  For Yes:   The authors justified combining the data in a meta-analysis   AND they used an appropriate weighted technique to combine study results, adjusting for heterogeneity if present   AND they statistically combined effect estimates from NRSI that were adjusted for confounding, rather than combining raw data, or justified combining raw data when adjusted effect estimates were not available   AND they reported separate summary estimates for RCTs and NRSI separately when both were included in the review | |  Yes   No  🗷 No meta-analysis conducted | | |  |
|  | Comments: None | | | | |  |
| **12. If meta-analysis was performed, did the review authors assess the potential impact of RoB in individual studies on the results of the meta-analysis or other evidence synthesis?** | | | | | | |
|  | For Yes:   included only low risk of bias RCTs   OR, if the pooled estimate was based on RCTs and/or NRSI at variable RoB, the authors performed analyses to investigate possible impact of RoB on summary estimates of effect. | |  Yes   No  🗷 No meta-analysis conducted | | |  |
|  | Comments: None | | | | |  |
| **13. Did the review authors account for RoB in individual studies when interpreting/ discussing the results of the review?** | | | | | | |
|  | For Yes:   included only low risk of bias RCTs   OR, if RCTs with moderate or high RoB, or NRSI were included the review provided a discussion of the likely impact of RoB on the results | |  Yes  🗷 No | | |  |
|  | Comments: None | | | | |  |
| **14. Did the review authors provide a satisfactory explanation for, and discussion of, any heterogeneity observed in the results of the review?** | | | | | | |
|  | For Yes:   There was no significant heterogeneity in the results   OR if heterogeneity was present the authors performed an investigation of sources of any heterogeneity in the results and discussed the impact of this on the results of the review | |  Yes  🗷 No | | |  |
|  | Comments: None | | | | |  |
| **15. If they performed quantitative synthesis did the review authors carry out an adequate investigation of publication bias (small study bias) and discuss its likely impact on the results of the review?** | | | | | | |
|  | For Yes:   performed graphical or statistical tests for publication bias and discussed the likelihood and magnitude of impact of publication bias | |  Yes   No  🗷 No meta-analysis conducted | | |  |
|  | Comments: None | | | | |  |
| **16. Did the review authors report any potential sources of conflict of interest, including any funding they received for conducting the review?** | | | | | | |
|  | For Yes:  🗷 The authors reported no competing interests OR   The authors described their funding sources and how they managed potential conflicts of interest | 🗷 Yes   No | | | |  |
|  | Comments: None | | | | |  |

# Study 10 – Hallesleben et al

| **1. Did the research questions and inclusion criteria for the review include the components of PICO?** | | | | | | | | |
| --- | --- | --- | --- | --- | --- | --- | --- | --- |
| For Yes:  🗷 Population  🗷 Intervention  🗷 Comparator group  🗷 Outcome | | | Optional (recommended)   Timeframe for follow-up | | 🗷 | | Yes No |  |
| Comments: None | | | | | | | |  |
| **2. Did the report of the review contain an explicit statement that the review methods were established prior to the conduct of the review and did the report justify any significant deviations from the protocol?** | | | | | | | | |
|  | For Partial Yes:  The authors state that they had a written protocol or guide that included ALL the following:   review question(s)   a search strategy   inclusion/exclusion criteria   a risk of bias assessment | | For Yes:  As for partial yes, plus the protocol should be registered and should also have specified:   a meta-analysis/synthesis plan, if appropriate, *and*   a plan for investigating causes of heterogeneity   justification for any deviations from the protocol | |   🗷 | | Yes  Partial Yes  No |  |
|  | Comments: There is not any reference to a published study protocol. | | | | | | |  |
| **3. Did the review authors explain their selection of the study designs for inclusion in the review?** | | | | | | | | |
|  | For Yes, the review should satisfy ONE of the following:   *Explanation for* including only RCTs   OR *Explanation for* including only NRSI  🗷 OR *Explanation for* including both RCTs and NRSI | | | | 🗷 | | Yes No |  |
|  | Comments: None | | | | | | |  |
| **4. Did the review authors use a comprehensive literature search strategy?** | | | | | | | | |
|  | For Partial Yes (all the following): | | For Yes, should also have (all the following):   searched the reference lists / bibliographies of included studies   searched trial/study registries   included/consulted content experts in the field  🗷 where relevant, searched for grey literature   conducted search within 24 months of completion of the review | |  | |  |  |
|  | 🗷 searched at least 2 databases (relevant to research question)  🗷 provided key word and/or search strategy  🗷 justified publication restrictions | |  |  |   🗷 | | Yes  Partial Yes No |  |
|  | (e.g. language) | |  |  |  | |  |  |
|  | Comments: None | | | | | | |  |
|  | **5. Did the review authors perform study selection in duplicate?** | | | |  | | |  |
|  | For Yes, either ONE of the following:   at least two reviewers independently agreed on selection of eligible studies and achieved consensus on which studies to include   OR two reviewers selected a sample of eligible studies and achieved good agreement (at least 80 percent), with the remainder selected by one reviewer. | | | | 🗷 | | Yes No |  |
|  | Comments: Only one reviewer selected studies. | | | | | | |  |
| **6. Did the review authors perform data extraction in duplicate?** | | | | | | | | |
| For Yes, either ONE of the following:  🗷 at least two reviewers achieved consensus on which data to extract from included studies   OR two reviewers extracted data from a sample of eligible studies and achieved good agreement (at least 80 percent), with the remainder extracted by one reviewer. | | | | | | 🗷 Yes   No | | |
| Comments: None | | | | | | | | |
| **7. Did the review authors provide a list of excluded studies and justify the exclusions?** | | | | | | | | |
|  | | For Partial Yes:   provided a list of all potentially relevant studies that were read in full-text form but excluded from the review | | For Yes, must also have:   Justified the exclusion from the review of each potentially relevant study | |  Yes   Partial Yes  🗷 No | | |
|  | | Comments: Not even described/ mentioned the overall reasons for study exclusion in the PRISMA flowchart (which also is missing). | | | | | | |
| **8. Did the review authors describe the included studies in adequate detail?** | | | | | | | | |
|  | | For Partial Yes (ALL the following):   described populations   described interventions   described comparators   described outcomes   described research designs | | For Yes, should also have ALL the following:   described population in detail   described intervention in detail (including doses where relevant)   described comparator in detail (including doses where relevant)   described study’s setting   timeframe for follow-up | | 🗷 Yes   Partial Yes   No | | |
|  | | Comments: Proper and wide description of application identified in the electronic searches. | | | | | | |
| **9. Did the review authors use a satisfactory technique for assessing the risk of bias (RoB) in individual studies that were included in the review?** | | | | | | | | |
|  | | **RCTs**  For Partial Yes, must have assessed RoB from   unconcealed allocation, *and*   lack of blinding of patients and assessors when assessing outcomes (unnecessary for objective outcomes such as all-cause mortality) | | For Yes, must also have assessed RoB from:   allocation sequence that was not truly random, *and*   selection of the reported result from among multiple measurements or analyses of a specified outcome | |  Yes   Partial Yes  🗷 No   Includes only NRSI | | |
|  | | **NRSI**  For Partial Yes, must have assessed RoB:   from confounding, *and*   from selection bias | | For Yes, must also have assessed RoB:   methods used to ascertain exposures and outcomes, *and*   selection of the reported result from among multiple measurements or analyses of a specified | |  Yes   Partial Yes  🗷 No  Includes only RCTs | | |
| Comments: Risk of bias not cited or considered anywhere. | | | | | | | | |

| **10. Did the review authors report outcomes on the sources of funding for the studies included in the review?** | | | | | | |
| --- | --- | --- | --- | --- | --- | --- |
| For Yes:   Must have reported on the sources of funding for individual studies included in the review.  Note: Reporting that the reviewers looked for this information but it was not reported by study authors also qualifies. | | | |  Yes  🗷 No |  |  |
| Comments: None | | | |  |  |  |
| **11. If meta-analysis was performed did the review authors use appropriate methods for statistical combination of results?** | | | | | | |
|  | **RCTs**  For Yes:   The authors justified combining the data in a meta-analysis   AND they used an appropriate weighted technique to combine study results and adjusted for heterogeneity if present.   AND investigated the causes of any heterogeneity | |  Yes   No  🗷 No meta-analysis conducted | | |  |
|  | **For NRSI**  For Yes:   The authors justified combining the data in a meta-analysis   AND they used an appropriate weighted technique to combine study results, adjusting for heterogeneity if present   AND they statistically combined effect estimates from NRSI that were adjusted for confounding, rather than combining raw data, or justified combining raw data when adjusted effect estimates were not available   AND they reported separate summary estimates for RCTs and NRSI separately when both were included in the review | |  Yes   No  🗷 No meta-analysis conducted | | |  |
|  | Comments: None | | | | |  |
| **12. If meta-analysis was performed, did the review authors assess the potential impact of RoB in individual studies on the results of the meta-analysis or other evidence synthesis?** | | | | | | |
|  | For Yes:   included only low risk of bias RCTs   OR, if the pooled estimate was based on RCTs and/or NRSI at variable RoB, the authors performed analyses to investigate possible impact of RoB on summary estimates of effect. | |  Yes   No  🗷 No meta-analysis conducted | | |  |
|  | Comments: None | | | | |  |
| **13. Did the review authors account for RoB in individual studies when interpreting/ discussing the results of the review?** | | | | | | |
|  | For Yes:   included only low risk of bias RCTs   OR, if RCTs with moderate or high RoB, or NRSI were included the review provided a discussion of the likely impact of RoB on the results | |  Yes  🗷 No | | |  |
|  | Comments: None | | | | |  |
| **14. Did the review authors provide a satisfactory explanation for, and discussion of, any heterogeneity observed in the results of the review?** | | | | | | |
|  | For Yes:   There was no significant heterogeneity in the results   OR if heterogeneity was present the authors performed an investigation of sources of any heterogeneity in the results and discussed the impact of this on the results of the review | |  Yes  🗷 No | | |  |
|  | Comments: None | | | | |  |
| **15. If they performed quantitative synthesis did the review authors carry out an adequate investigation of publication bias (small study bias) and discuss its likely impact on the results of the review?** | | | | | | |
|  | For Yes:   performed graphical or statistical tests for publication bias and discussed the likelihood and magnitude of impact of publication bias | |  Yes   No  🗷 No meta-analysis conducted | | |  |
|  | Comments: None | | | | |  |
| **16. Did the review authors report any potential sources of conflict of interest, including any funding they received for conducting the review?** | | | | | | |
|  | For Yes:  🗷 The authors reported no competing interests OR   The authors described their funding sources and how they managed potential conflicts of interest | 🗷 Yes   No | | | |  |
|  | Comments: None | | | | |  |

# Study 11 – Hartasanchez et al

| **1. Did the research questions and inclusion criteria for the review include the components of PICO?** | | | | | | | | |
| --- | --- | --- | --- | --- | --- | --- | --- | --- |
| For Yes:  🗷 Population  🗷 Intervention  🗷 Comparator group  🗷 Outcome | | | Optional (recommended)   Timeframe for follow-up | | 🗷 | | Yes No |  |
| Comments: None | | | | | | | |  |
| **2. Did the report of the review contain an explicit statement that the review methods were established prior to the conduct of the review and did the report justify any significant deviations from the protocol?** | | | | | | | | |
|  | For Partial Yes:  The authors state that they had a written protocol or guide that included ALL the following:   review question(s)   a search strategy   inclusion/exclusion criteria   a risk of bias assessment | | For Yes:  As for partial yes, plus the protocol should be registered and should also have specified:   a meta-analysis/synthesis plan, if appropriate, *and*   a plan for investigating causes of heterogeneity   justification for any deviations from the protocol | |   🗷 | | Yes  Partial Yes  No |  |
|  | Comments: The authors stated “This protocol-guided systematic review” in the first sentence of the methodology section. However, even after a detailed analysis of supplementary material, we did not find any evidence of the existence of a protocol. | | | | | | |  |
| **3. Did the review authors explain their selection of the study designs for inclusion in the review?** | | | | | | | | |
|  | For Yes, the review should satisfy ONE of the following:   *Explanation for* including only RCTs   OR *Explanation for* including only NRSI  🗷 OR *Explanation for* including both RCTs and NRSI | | | | 🗷 | | Yes No |  |
|  | Comments: None | | | | | | |  |
| **4. Did the review authors use a comprehensive literature search strategy?** | | | | | | | | |
|  | For Partial Yes (all the following): | | For Yes, should also have (all the following):   searched the reference lists / bibliographies of included studies   searched trial/study registries   included/consulted content experts in the field   where relevant, searched for grey literature   conducted search within 24 months of completion of the review | |  | |  |  |
|  | 🗷 searched at least 2 databases (relevant to research question)  🗷 provided key word and/or search strategy  🗷 justified publication restrictions | |  |  |   🗷 | | Yes  Partial Yes No |  |
|  | (e.g. language) | |  |  |  | |  |  |
|  | Comments: All electronic search strategies are in the Supplementary material. | | | | | | |  |
|  | **5. Did the review authors perform study selection in duplicate?** | | | |  | | |  |
|  | For Yes, either ONE of the following:  🗷 at least two reviewers independently agreed on selection of eligible studies and achieved consensus on which studies to include   OR two reviewers selected a sample of eligible studies and achieved good agreement (at least 80 percent), with the remainder selected by one reviewer. | | | | 🗷 | | Yes No |  |
|  | Comments: None | | | | | | |  |
| **6. Did the review authors perform data extraction in duplicate?** | | | | | | | | |
| For Yes, either ONE of the following:  🗷 at least two reviewers achieved consensus on which data to extract from included studies   OR two reviewers extracted data from a sample of eligible studies and achieved good agreement (at least 80 percent), with the remainder extracted by one reviewer. | | | | | | 🗷 Yes   No | | |
| Comments: None | | | | | | | | |
| **7. Did the review authors provide a list of excluded studies and justify the exclusions?** | | | | | | | | |
|  | | For Partial Yes:   provided a list of all potentially relevant studies that were read in full-text form but excluded from the review | | For Yes, must also have:   Justified the exclusion from the review of each potentially relevant study | |  Yes   Partial Yes  🗷 No | | |
|  | | Comments: Only described/ mentioned the overall reasons for study exclusion in the PRISMA flowchart. | | | | | | |
| **8. Did the review authors describe the included studies in adequate detail?** | | | | | | | | |
|  | | For Partial Yes (ALL the following):  🗷 described populations  🗷 described interventions  🗷 described comparators  🗷 described outcomes  🗷 described research designs | | For Yes, should also have ALL the following:   described population in detail   described intervention in detail (including doses where relevant)   described comparator in detail (including doses where relevant)   described study’s setting   timeframe for follow-up | |  Yes  🗷 Partial Yes   No | | |
|  | | Comments: None | | | | | | |
| **9. Did the review authors use a satisfactory technique for assessing the risk of bias (RoB) in individual studies that were included in the review?** | | | | | | | | |
|  | | **RCTs**  For Partial Yes, must have assessed RoB from   unconcealed allocation, *and*   lack of blinding of patients and assessors when assessing outcomes (unnecessary for objective outcomes such as all-cause mortality) | | For Yes, must also have assessed RoB from:   allocation sequence that was not truly random, *and*   selection of the reported result from among multiple measurements or analyses of a specified outcome | |  Yes   Partial Yes  🗷 No   Includes only NRSI | | |
|  | | **NRSI**  For Partial Yes, must have assessed RoB:   from confounding, *and*   from selection bias | | For Yes, must also have assessed RoB:   methods used to ascertain exposures and outcomes, *and*   selection of the reported result from among multiple measurements or analyses of a specified | |  Yes   Partial Yes  🗷 No  Includes only RCTs | | |
| Comments: RoB not considered. | | | | | | | | |

| **10. Did the review authors report outcomes on the sources of funding for the studies included in the review?** | | | | | | |
| --- | --- | --- | --- | --- | --- | --- |
| For Yes:   Must have reported on the sources of funding for individual studies included in the review.  Note: Reporting that the reviewers looked for this information but it was not reported by study authors also qualifies. | | | |  Yes  🗷 No |  |  |
| Comments: None | | | |  |  |  |
| **11. If meta-analysis was performed did the review authors use appropriate methods for statistical combination of results?** | | | | | | |
|  | **RCTs**  For Yes:   The authors justified combining the data in a meta-analysis   AND they used an appropriate weighted technique to combine study results and adjusted for heterogeneity if present.   AND investigated the causes of any heterogeneity | |  Yes   No  🗷 No meta-analysis conducted | | |  |
|  | **For NRSI**  For Yes:   The authors justified combining the data in a meta-analysis   AND they used an appropriate weighted technique to combine study results, adjusting for heterogeneity if present   AND they statistically combined effect estimates from NRSI that were adjusted for confounding, rather than combining raw data, or justified combining raw data when adjusted effect estimates were not available   AND they reported separate summary estimates for RCTs and NRSI separately when both were included in the review | |  Yes   No  🗷 No meta-analysis conducted | | |  |
|  | Comments: None | | | | |  |
| **12. If meta-analysis was performed, did the review authors assess the potential impact of RoB in individual studies on the results of the meta-analysis or other evidence synthesis?** | | | | | | |
|  | For Yes:   included only low risk of bias RCTs   OR, if the pooled estimate was based on RCTs and/or NRSI at variable RoB, the authors performed analyses to investigate possible impact of RoB on summary estimates of effect. | |  Yes   No  🗷 No meta-analysis conducted | | |  |
|  | Comments: None | | | | |  |
| **13. Did the review authors account for RoB in individual studies when interpreting/ discussing the results of the review?** | | | | | | |
|  | For Yes:   included only low risk of bias RCTs   OR, if RCTs with moderate or high RoB, or NRSI were included the review provided a discussion of the likely impact of RoB on the results | |  Yes  🗷 No | | |  |
|  | Comments: None | | | | |  |
| **14. Did the review authors provide a satisfactory explanation for, and discussion of, any heterogeneity observed in the results of the review?** | | | | | | |
|  | For Yes:   There was no significant heterogeneity in the results   OR if heterogeneity was present the authors performed an investigation of sources of any heterogeneity in the results and discussed the impact of this on the results of the review | |  Yes  🗷 No | | |  |
|  | Comments: None | | | | |  |
| **15. If they performed quantitative synthesis did the review authors carry out an adequate investigation of publication bias (small study bias) and discuss its likely impact on the results of the review?** | | | | | | |
|  | For Yes:   performed graphical or statistical tests for publication bias and discussed the likelihood and magnitude of impact of publication bias | |  Yes   No  🗷 No meta-analysis conducted | | |  |
|  | Comments: None | | | | |  |
| **16. Did the review authors report any potential sources of conflict of interest, including any funding they received for conducting the review?** | | | | | | |
|  | For Yes:  🗷 The authors reported no competing interests OR   The authors described their funding sources and how they managed potential conflicts of interest | 🗷 Yes   No | | | |  |
|  | Comments: None | | | | |  |

# Study 12 – Hrynyschyn et al

| **1. Did the research questions and inclusion criteria for the review include the components of PICO?** | | | | | | | | |
| --- | --- | --- | --- | --- | --- | --- | --- | --- |
| For Yes:  🗷 Population  🗷 Intervention  🗷 Comparator group  🗷 Outcome | | | Optional (recommended)   Timeframe for follow-up | | 🗷 | | Yes No |  |
| Comments: None | | | | | | | |  |
| **2. Did the report of the review contain an explicit statement that the review methods were established prior to the conduct of the review and did the report justify any significant deviations from the protocol?** | | | | | | | | |
|  | For Partial Yes:  The authors state that they had a written protocol or guide that included ALL the following:   review question(s)   a search strategy   inclusion/exclusion criteria   a risk of bias assessment | | For Yes:  As for partial yes, plus the protocol should be registered and should also have specified:   a meta-analysis/synthesis plan, if appropriate, *and*   a plan for investigating causes of heterogeneity   justification for any deviations from the protocol | |   🗷 | | Yes  Partial Yes  No |  |
|  | Comments: There is not any reference to a published study protocol. | | | | | | |  |
| **3. Did the review authors explain their selection of the study designs for inclusion in the review?** | | | | | | | | |
|  | For Yes, the review should satisfy ONE of the following:   *Explanation for* including only RCTs   OR *Explanation for* including only NRSI  🗷 OR *Explanation for* including both RCTs and NRSI | | | | 🗷 | | Yes No |  |
|  | Comments: None | | | | | | |  |
| **4. Did the review authors use a comprehensive literature search strategy?** | | | | | | | | |
|  | For Partial Yes (all the following): | | For Yes, should also have (all the following):  🗷 searched the reference lists / bibliographies of included studies   searched trial/study registries   included/consulted content experts in the field   where relevant, searched for grey literature   conducted search within 24 months of completion of the review | |  | |  |  |
|  | 🗷 searched at least 2 databases (relevant to research question)  🗷 provided key word and/or search strategy  🗷 justified publication restrictions | |  |  |    | | Yes  Partial Yes No |  |
|  | (e.g. language) | |  |  |  | |  |  |
|  | Comments: Only studies in German and English | | | | | | |  |
|  | **5. Did the review authors perform study selection in duplicate?** | | | |  | | |  |
|  | For Yes, either ONE of the following:  🗷 at least two reviewers independently agreed on selection of eligible studies and achieved consensus on which studies to include   OR two reviewers selected a sample of eligible studies and achieved good agreement (at least 80 percent), with the remainder selected by one reviewer. | | | | 🗷 | | Yes No |  |
|  | Comments: None | | | | | | |  |
| **6. Did the review authors perform data extraction in duplicate?** | | | | | | | | |
| For Yes, either ONE of the following:  🗷 at least two reviewers achieved consensus on which data to extract from included studies   OR two reviewers extracted data from a sample of eligible studies and achieved good agreement (at least 80 percent), with the remainder extracted by one reviewer. | | | | | | 🗷 Yes   No | | |
| Comments: None | | | | | | | | |
| **7. Did the review authors provide a list of excluded studies and justify the exclusions?** | | | | | | | | |
|  | | For Partial Yes:   provided a list of all potentially relevant studies that were read in full-text form but excluded from the review | | For Yes, must also have:   Justified the exclusion from the review of each potentially relevant study | |  Yes   Partial Yes  🗷 No | | |
|  | | Comments: Only described/ mentioned the overall reasons for study exclusion in the PRISMA flowchart. | | | | | | |
| **8. Did the review authors describe the included studies in adequate detail?** | | | | | | | | |
|  | | For Partial Yes (ALL the following):  🗷 described populations  🗷 described interventions  🗷 described comparators  🗷 described outcomes  🗷 described research designs | | For Yes, should also have ALL the following:  🗷 described population in detail  🗷 described intervention in detail (including doses where relevant)  🗷 described comparator in detail (including doses where relevant)  🗷 described study’s setting  🗷 timeframe for follow-up | | 🗷 Yes   Partial Yes   No | | |
|  | | Comments: None | | | | | | |
| **9. Did the review authors use a satisfactory technique for assessing the risk of bias (RoB) in individual studies that were included in the review?** | | | | | | | | |
|  | | **RCTs**  For Partial Yes, must have assessed RoB from  🗷 unconcealed allocation, *and*  🗷 lack of blinding of patients and assessors when assessing outcomes (unnecessary for objective outcomes such as all-cause mortality) | | For Yes, must also have assessed RoB from:  🗷 allocation sequence that was not truly random, *and*  🗷 selection of the reported result from among multiple measurements or analyses of a specified outcome | | 🗷 Yes   Partial Yes   No   Includes only NRSI | | |
|  | | **NRSI**  For Partial Yes, must have assessed RoB:   from confounding, *and*   from selection bias | | For Yes, must also have assessed RoB:   methods used to ascertain exposures and outcomes, *and*   selection of the reported result from among multiple measurements or analyses of a specified | |  Yes   Partial Yes  🗷 No  Includes only RCTs | | |
| Comments: Quality assessment tool described in Table 1 (“Tab 1. Instrument zur Bewertung der Qualität der eingeschossenen Studien”). | | | | | | | | |

| **10. Did the review authors report outcomes on the sources of funding for the studies included in the review?** | | | | | | |
| --- | --- | --- | --- | --- | --- | --- |
| For Yes:   Must have reported on the sources of funding for individual studies included in the review.  Note: Reporting that the reviewers looked for this information but it was not reported by study authors also qualifies. | | | |  Yes  🗷 No |  |  |
| Comments: None | | | |  |  |  |
| **11. If meta-analysis was performed did the review authors use appropriate methods for statistical combination of results?** | | | | | | |
|  | **RCTs**  For Yes:   The authors justified combining the data in a meta-analysis   AND they used an appropriate weighted technique to combine study results and adjusted for heterogeneity if present.   AND investigated the causes of any heterogeneity | |  Yes   No  🗷 No meta-analysis conducted | | |  |
|  | **For NRSI**  For Yes:   The authors justified combining the data in a meta-analysis   AND they used an appropriate weighted technique to combine study results, adjusting for heterogeneity if present   AND they statistically combined effect estimates from NRSI that were adjusted for confounding, rather than combining raw data, or justified combining raw data when adjusted effect estimates were not available   AND they reported separate summary estimates for RCTs and NRSI separately when both were included in the review | |  Yes   No  🗷 No meta-analysis conducted | | |  |
|  | Comments: | | | | |  |
| **12. If meta-analysis was performed, did the review authors assess the potential impact of RoB in individual studies on the results of the meta-analysis or other evidence synthesis?** | | | | | | |
|  | For Yes:   included only low risk of bias RCTs   OR, if the pooled estimate was based on RCTs and/or NRSI at variable RoB, the authors performed analyses to investigate possible impact of RoB on summary estimates of effect. | |  Yes   No  🗷 No meta-analysis conducted | | |  |
|  | Comments: | | | | |  |
| **13. Did the review authors account for RoB in individual studies when interpreting/ discussing the results of the review?** | | | | | | |
|  | For Yes:   included only low risk of bias RCTs  🗷 OR, if RCTs with moderate or high RoB, or NRSI were included the review provided a discussion of the likely impact of RoB on the results | | 🗷 Yes   No | | |  |
|  | Comments: None | | | | |  |
| **14. Did the review authors provide a satisfactory explanation for, and discussion of, any heterogeneity observed in the results of the review?** | | | | | | |
|  | For Yes:   There was no significant heterogeneity in the results   OR if heterogeneity was present the authors performed an investigation of sources of any heterogeneity in the results and discussed the impact of this on the results of the review | |  Yes  🗷 No | | |  |
|  | Comments: Heterogeneity was vaguely mentioned in the text “with regards to chronic wounds examined”. | | | | |  |
| **15. If they performed quantitative synthesis did the review authors carry out an adequate investigation of publication bias (small study bias) and discuss its likely impact on the results of the review?** | | | | | | |
|  | For Yes:   performed graphical or statistical tests for publication bias and discussed the likelihood and magnitude of impact of publication bias | |  Yes   No  🗷 No meta-analysis conducted | | |  |
|  | Comments: None | | | | |  |
| **16. Did the review authors report any potential sources of conflict of interest, including any funding they received for conducting the review?** | | | | | | |
|  | For Yes:  🗷 The authors reported no competing interests OR   The authors described their funding sources and how they managed potential conflicts of interest | 🗷 Yes   No | | | |  |
|  | Comments: None | | | | |  |

# Study 13 – Karamanidou et al

| **1. Did the research questions and inclusion criteria for the review include the components of PICO?** | | | | | | | | |
| --- | --- | --- | --- | --- | --- | --- | --- | --- |
| For Yes:  x Population  x Intervention  n/a Comparator group  x Outcome | | | Optional (recommended)   Timeframe for follow-up | | x | | Y N |  |
| Comments: As this was a systematic review C-comparison not applicable. | | | | | | | |  |
| **2. Did the report of the review contain an explicit statement that the review methods were established prior to the conduct of the review and did the report justify any significant deviations from the protocol?** | | | | | | | | |
|  | For Partial Yes:  The authors state that they had a written protocol or guide that included ALL the following:  x review question(s)  x a search strategy  x inclusion/exclusion criteria  x a risk of bias assessment | | For Yes:  As for partial yes, plus the protocol should be registered and should also have specified:   a meta-analysis/synthesis plan, if appropriate, *and*   a plan for investigating causes of heterogeneity   justification for any deviations from the protocol | | x | | Yes  Partial Yes  No |  |
|  | Comments: This was a systematic review and mapping review, the protocol was not registered. | | | | | | |  |
| **3. Did the review authors explain their selection of the study designs for inclusion in the review?** | | | | | | | | |
|  | For Yes, the review should satisfy ONE of the following:   *Explanation for* including only RCTs   OR *Explanation for* including only NRSI   OR *Explanation for* including both RCTs and NRSI | | | | x | | Yes No |  |
|  | Comments: An explicit explanation is not provided, but the authors included all types of study designs. | | | | | | |  |
| **4. Did the review authors use a comprehensive literature search strategy?** | | | | | | | | |
|  | For Partial Yes (all the following): | | For Yes, should also have (all the following):   searched the reference lists / bibliographies of included studies   searched trial/study registries   included/consulted content experts in the field   where relevant, searched for grey literature   conducted search within 24 months of completion of the review | |  | |  |  |
|  | x searched at least 2 databases (relevant to research question)  x provided key word and/or search strategy  x justified publication restrictions | |  |  |   x  | | Yes  Partial Yes No |  |
|  | (e.g. language) | |  |  |  | |  |  |
|  | Comments: Provided justification for restricting to 2 databases. | | | | | | |  |
|  | **5. Did the review authors perform study selection in duplicate?** | | | |  | | |  |
|  | For Yes, either ONE of the following:  x at least two reviewers independently agreed on selection of eligible studies and achieved consensus on which studies to include   OR two reviewers selected a sample of eligible studies and achieved good agreement (at least 80 percent), with the remainder selected by one reviewer. | | | | x  | | Yes No |  |
|  | Comments: Study states *3 of the authors independently assessed the eligibility of each article (Inclusion and Exclusion Criteria) on the basis of the information contained in the article title and abstract, whenever possible.* | | | | | | |  |
| **6. Did the review authors perform data extraction in duplicate?** | | | | | | | | |
| For Yes, either ONE of the following:  x at least two reviewers achieved consensus on which data to extract from included studies   OR two reviewers extracted data from a sample of eligible studies and achieved good agreement (at least 80 percent), with the remainder extracted by one reviewer. | | | | | |  Yes   No | | |
| Comments: Comments: Thought to meet the criteria, but not completely clear. The text states: *The eligible articles were qualitatively and quantitatively mapped across a number of mapping criteria/axes. These were defined on the basis of the expertise of the authors in digital health systems and palliative care, as well as on the particular focus of the MyPal program; iterative fine-tuning of the mapping axes was performed as the outcomes from the articles were gradually obtained to ensure that the axes were orthogonal (ie, nonoverlapping) to the greatest extent possible. The final list of mapping axes that was used in the study is provided in Table 1.* | | | | | | | | |
| **7. Did the review authors provide a list of excluded studies and justify the exclusions?** | | | | | | | | |
|  | | For Partial Yes:   provided a list of all potentially relevant studies that were read in full-text form but excluded from the review | | For Yes, must also have:   Justified the exclusion from the review of each potentially relevant study | |  Yes   Partial Yes  x No | | |
|  | | Comments: Only the exclusion criteria listed in the PRISMA, no full list provided | | | | | | |
| **8. Did the review authors describe the included studies in adequate detail?** | | | | | | | | |
|  | | For Partial Yes (ALL the following):  x described populations  x described interventions  n/a described comparators  x described outcomes  x described research designs | | For Yes, should also have ALL the following:  x described population in detail  x described intervention in detail (including doses where relevant)  n/a described comparator in detail (including doses where relevant)  x described study’s setting  n/a timeframe for follow-up | |  Yes  s Partial Yes   No | | |
|  | | Comments: The Mapping Axes Specified by the Review Protocol includes items listed above however this was not included in the paper. In the publication the only data provided is author, year, journal, country, and organization type. | | | | | | |
| **9. Did the review authors use a satisfactory technique for assessing the risk of bias (RoB) in individual studies that were included in the review?** | | | | | | | | |
|  | | **RCTs**  For Partial Yes, must have assessed RoB from   unconcealed allocation, *and*   lack of blinding of patients and assessors when assessing outcomes (unnecessary for objective outcomes such as all-cause mortality) | | For Yes, must also have assessed RoB from:   allocation sequence that was not truly random, *and*   selection of the reported result from among multiple measurements or analyses of a specified outcome | |  Yes   Partial Yes  x No   Includes only NRSI | | |
|  | | **NRSI**  For Partial Yes, must have assessed RoB:   from confounding, *and*   from selection bias | | For Yes, must also have assessed RoB:   methods used to ascertain exposures and outcomes, *and*   selection of the reported result from among multiple measurements or analyses of a specified | |  Yes   Partial Yes  x No  Includes only RCTs | | |
| Comments: This is a systematic review and mapping review and therefore this is not applicable. | | | | | | | | |

| **10. Did the review authors report outcomes on the sources of funding for the studies included in the review?** | | | | | | |
| --- | --- | --- | --- | --- | --- | --- |
| For Yes:   Must have reported on the sources of funding for individual studies included in the review.  Note: Reporting that the reviewers looked for this information but it was not reported by study authors also qualifies. | | | | x Yes   No |  |  |
| Comments: Funding for the review is stated: *European Union’s Horizon 2020 Research and Innovation Programme Grant No. 825872. The funder played no role in the systematic review.* | | | |  |  |  |
| **11. If meta-analysis was performed did the review authors use appropriate methods for statistical combination of results?** | | | | | | |
|  | **RCTs**  For Yes:   The authors justified combining the data in a meta-analysis   AND they used an appropriate weighted technique to combine study results and adjusted for heterogeneity if present.   AND investigated the causes of any heterogeneity | |  Yes   No  x No meta-analysis conducted | | |  |
|  | **For NRSI**  For Yes:   The authors justified combining the data in a meta-analysis   AND they used an appropriate weighted technique to combine study results, adjusting for heterogeneity if present   AND they statistically combined effect estimates from NRSI that were adjusted for confounding, rather than combining raw data, or justified combining raw data when adjusted effect estimates were not available   AND they reported separate summary estimates for RCTs and NRSI separately when both were included in the review | |  Yes   No  x No meta-analysis conducted | | |  |
|  | Comments: Not applicable | | | | |  |
| **12. If meta-analysis was performed, did the review authors assess the potential impact of RoB in individual studies on the results of the meta-analysis or other evidence synthesis?** | | | | | | |
|  | For Yes:   included only low risk of bias RCTs   OR, if the pooled estimate was based on RCTs and/or NRSI at variable RoB, the authors performed analyses to investigate possible impact of RoB on summary estimates of effect. | |  Yes   No   x No meta-analysis conducted | | |  |
|  | Comments: Not applicable | | | | |  |
| **13. Did the review authors account for RoB in individual studies when interpreting/ discussing the results of the review?** | | | | | | |
|  | For Yes:   included only low risk of bias RCTs   OR, if RCTs with moderate or high RoB, or NRSI were included the review provided a discussion of the likely impact of RoB on the results | |  Yes  x No | | |  |
|  | Comments: This is a systematic review and mapping review. The paper reports that a ‘risk of bias and mitigation strategy’ was applied to the analysis of the research including strategies for mitigating bias of: reporting, competing interests, and evidence selection bias. Explanation for each provided. | | | | |  |
| **14. Did the review authors provide a satisfactory explanation for, and discussion of, any heterogeneity observed in the results of the review?** | | | | | | |
|  | For Yes:   There was no significant heterogeneity in the results   OR if heterogeneity was present the authors performed an investigation of sources of any heterogeneity in the results and discussed the impact of this on the results of the review | | x Yes   No | | |  |
|  | Comments: Heterogeneity is not explicitly discussed but the variety of interventions is discussed throughout | | | | |  |
| **15. If they performed quantitative synthesis did the review authors carry out an adequate investigation of publication bias (small study bias) and discuss its likely impact on the results of the review?** | | | | | | |
|  | For Yes:   performed graphical or statistical tests for publication bias and discussed the likelihood and magnitude of impact of publication bias | |  Yes   No  x No meta-analysis conducted | | |  |
|  | Comments: Not applicable | | | | |  |
| **16. Did the review authors report any potential sources of conflict of interest, including any funding they received for conducting the review?** | | | | | | |
|  | For Yes:  x The authors reported no competing interests OR   The authors described their funding sources and how they managed potential conflicts of interest | x Yes   No | | | |  |
|  | Comments: No potential conflicts of interest were reported. | | | | |  |

# Study 14 – Keirkegaard et al

| **1. Did the research questions and inclusion criteria for the review include the components of PICO?** | | | | | | | | |
| --- | --- | --- | --- | --- | --- | --- | --- | --- |
| For Yes:  x Population  x Intervention  n/a Comparator group  n/a Outcome | | | Optional (recommended)   Timeframe for follow-up | | x | | Yes No |  |
| Comments: This is a mapping study, comparator and outcome is not applicable. | | | | | | | |  |
| **2. Did the report of the review contain an explicit statement that the review methods were established prior to the conduct of the review and did the report justify any significant deviations from the protocol?** | | | | | | | | |
|  | For Partial Yes:  The authors state that they had a written protocol or guide that included ALL the following:  x review question(s)  x a search strategy  n/a inclusion/exclusion criteria  n/a a risk of bias assessment | | For Yes:  As for partial yes, plus the protocol should be registered and should also have specified:   a meta-analysis/synthesis plan, if appropriate, *and*   a plan for investigating causes of heterogeneity   justification for any deviations from the protocol | |   x | | Yes  Partial Yes  No |  |
|  | Comments: This is a mapping study, all the data included in the Telemedicinsk Landkort database was included | | | | | | |  |
| **3. Did the review authors explain their selection of the study designs for inclusion in the review?** | | | | | | | | |
|  | For Yes, the review should satisfy ONE of the following:   *Explanation for* including only RCTs   OR *Explanation for* including only NRSI   OR *Explanation for* including both RCTs and NRSI | | | | x  | | Yes No |  |
|  | Comments: All the current projects in the database were included as stated in the study: *350 telemedicine projects were listed in the database, of which 125 were categorized as established services and 225 were categorized as nonoperational projects (including projects in the planning phase, pilots, and completed trials). Nonoperational telemedicine projects were excluded from the analysis of this study because they were not considered established services in the daily workflow of healthcare professionals and did not involve regular with interaction* with larger patient groups. | | | | | | |  |
| **4. Did the review authors use a comprehensive literature search strategy?** | | | | | | | | |
|  | For Partial Yes (all the following): | | For Yes, should also have (all the following):   searched the reference lists / bibliographies of included studies   searched trial/study registries   included/consulted content experts in the field   where relevant, searched for grey literature   conducted search within 24 months of completion of the review | |  | |  |  |
|  |  searched at least 2 databases (relevant to research question)   provided key word and/or search strategy  x justified publication restrictions | |  |  |     x | | Yes  Partial Yes No |  |
|  | (e.g. language) | |  |  |  | |  |  |
|  | Comments: Not applicable since all the current projects in the Telemedicinsk Landkort database were included. | | | | | | |  |
|  | **5. Did the review authors perform study selection in duplicate?** | | | |  | | |  |
|  | For Yes, either ONE of the following:   at least two reviewers independently agreed on selection of eligible studies and achieved consensus on which studies to include   OR two reviewers selected a sample of eligible studies and achieved good agreement (at least 80 percent), with the remainder selected by one reviewer. | | | | x | | Yes No |  |
|  | Comments: Not applicable to this mapping study | | | | | | |  |
| **6. Did the review authors perform data extraction in duplicate?** | | | | | | | | |
| For Yes, either ONE of the following:   at least two reviewers achieved consensus on which data to extract from included studies   OR two reviewers extracted data from a sample of eligible studies and achieved good agreement (at least 80 percent), with the remainder extracted by one reviewer. | | | | | |  Yes  x No | | |
| Comments: Not applicable for this mapping study. | | | | | | | | |
| **7. Did the review authors provide a list of excluded studies and justify the exclusions?** | | | | | | | | |
|  | | For Partial Yes:   provided a list of all potentially relevant studies that were read in full-text form but excluded from the review | | For Yes, must also have:   Justified the exclusion from the review of each potentially relevant study | |  Yes   Partial Yes  x No | | |
|  | | Comments: Not applicable for this mapping study. Excluded project were those that were not current. | | | | | | |
| **8. Did the review authors describe the included studies in adequate detail?** | | | | | | | | |
|  | | For Partial Yes (ALL the following):   described populations   described interventions   described comparators   described outcomes   described research designs | | For Yes, should also have ALL the following:   described population in detail   described intervention in detail (including doses where relevant)   described comparator in detail (including doses where relevant)   described study’s setting   timeframe for follow-up | |  Yes   Partial Yes  x No | | |
|  | | Comments: Specific Projects are not provided. Frequency tables provided by telemedicine specialty, location, type of activity, user groups. | | | | | | |
| **9. Did the review authors use a satisfactory technique for assessing the risk of bias (RoB) in individual studies that were included in the review?** | | | | | | | | |
|  | | **RCTs**  For Partial Yes, must have assessed RoB from   unconcealed allocation, *and*   lack of blinding of patients and assessors when assessing outcomes (unnecessary for objective outcomes such as all-cause mortality) | | For Yes, must also have assessed RoB from:   allocation sequence that was not truly random, *and*   selection of the reported result from among multiple measurements or analyses of a specified outcome | |  Yes   Partial Yes  x No   Includes only NRSI | | |
|  | | **NRSI**  For Partial Yes, must have assessed RoB:   from confounding, *and*   from selection bias | | For Yes, must also have assessed RoB:   methods used to ascertain exposures and outcomes, *and*   selection of the reported result from among multiple measurements or analyses of a specified | |  Yes   Partial Yes  x No  Includes only RCTs | | |
| Comments: Not applicable for this mapping study. | | | | | | | | |

| **10. Did the review authors report outcomes on the sources of funding for the studies included in the review?** | | | | | | |
| --- | --- | --- | --- | --- | --- | --- |
| For Yes:   Must have reported on the sources of funding for individual studies included in the review.  Note: Reporting that the reviewers looked for this information but it was not reported by study authors also qualifies. | | | |  Yes  x No |  |  |
| Comments: funding information provided as a frequency summary table but not for individual projects | | | |  |  |  |
| **11. If meta-analysis was performed did the review authors use appropriate methods for statistical combination of results?** | | | | | | |
|  | **RCTs**  For Yes:   The authors justified combining the data in a meta-analysis   AND they used an appropriate weighted technique to combine study results and adjusted for heterogeneity if present.   AND investigated the causes of any heterogeneity | |  Yes   No  x No meta-analysis conducted | | |  |
|  | **For NRSI**  For Yes:   The authors justified combining the data in a meta-analysis   AND they used an appropriate weighted technique to combine study results, adjusting for heterogeneity if present   AND they statistically combined effect estimates from NRSI that were adjusted for confounding, rather than combining raw data, or justified combining raw data when adjusted effect estimates were not available   AND they reported separate summary estimates for RCTs and NRSI separately when both were included in the review | |  Yes   No  x No meta-analysis conducted | | |  |
|  | Comments: Not applicable | | | | |  |
| **12. If meta-analysis was performed, did the review authors assess the potential impact of RoB in individual studies on the results of the meta-analysis or other evidence synthesis?** | | | | | | |
|  | For Yes:   included only low risk of bias RCTs   OR, if the pooled estimate was based on RCTs and/or NRSI at variable RoB, the authors performed analyses to investigate possible impact of RoB on summary estimates of effect. | |  Yes   No   x No meta-analysis conducted | | |  |
|  | Comments: Not applicable | | | | |  |
| **13. Did the review authors account for RoB in individual studies when interpreting/ discussing the results of the review?** | | | | | | |
|  | For Yes:   included only low risk of bias RCTs   OR, if RCTs with moderate or high RoB, or NRSI were included the review provided a discussion of the likely impact of RoB on the results | |  Yes  x No | | |  |
|  | Comments: Not applicable for this mapping study. | | | | |  |
| **14. Did the review authors provide a satisfactory explanation for, and discussion of, any heterogeneity observed in the results of the review?** | | | | | | |
|  | For Yes:   There was no significant heterogeneity in the results   OR if heterogeneity was present the authors performed an investigation of sources of any heterogeneity in the results and discussed the impact of this on the results of the review | | x Yes   No | | |  |
|  | Comments: Heterogeneity is not explicitly discussed but the variety of interventions is discussed throughout | | | | |  |
| **15. If they performed quantitative synthesis did the review authors carry out an adequate investigation of publication bias (small study bias) and discuss its likely impact on the results of the review?** | | | | | | |
|  | For Yes:   performed graphical or statistical tests for publication bias and discussed the likelihood and magnitude of impact of publication bias | |  Yes   No  x No meta-analysis conducted | | |  |
|  | Comments: | | | | |  |
| **16. Did the review authors report any potential sources of conflict of interest, including any funding they received for conducting the review?** | | | | | | |
|  | For Yes:   The authors reported no competing interests OR  x The authors described their funding sources and how they managed potential conflicts of interest | x Yes   No | | | |  |
|  | Comments: Conflict of interest not explicitly discussed, but the paper states ‘No competing financial interest exist.’ | | | | |  |

# Study 15 – Kingsdorf et al

| **1. Did the research questions and inclusion criteria for the review include the components of PICO?** | | | | | | | | |
| --- | --- | --- | --- | --- | --- | --- | --- | --- |
| For Yes:  x Population  x Intervention  n/a Comparator group  x Outcome | | | Optional (recommended)   Timeframe for follow-up | | x | | Yes No |  |
| Comments: As this was a scoping review C-comparison not applicable. | | | | | | | |  |
| **2. Did the report of the review contain an explicit statement that the review methods were established prior to the conduct of the review and did the report justify any significant deviations from the protocol?** | | | | | | | | |
|  | For Partial Yes:  The authors state that they had a written protocol or guide that included ALL the following:  x review question(s)  x a search strategy  x inclusion/exclusion criteria  n/a a risk of bias assessment | | For Yes:  As for partial yes, plus the protocol should be registered and should also have specified:   a meta-analysis/synthesis plan, if appropriate, *and*   a plan for investigating causes of heterogeneity   justification for any deviations from the protocol | |   x | | Yes  Partial Yes  No |  |
|  | Comments: For a scoping study, risk of bias is not usually included. | | | | | | |  |
| **3. Did the review authors explain their selection of the study designs for inclusion in the review?** | | | | | | | | |
|  | For Yes, the review should satisfy ONE of the following:   *Explanation for* including only RCTs   OR *Explanation for* including only NRSI   OR *Explanation for* including both RCTs and NRSI | | | | x | | Yes No |  |
|  | Comments: An explicit explanation not provided, but the authors included all types of study designs. | | | | | | |  |
| **4. Did the review authors use a comprehensive literature search strategy?** | | | | | | | | |
|  | For Partial Yes (all the following): | | For Yes, should also have (all the following):   searched the reference lists / bibliographies of included studies   searched trial/study registries   included/consulted content experts in the field   where relevant, searched for grey literature   conducted search within 24 months of completion of the review | |  | |  |  |
|  | x searched at least 2 databases (relevant to research question)  x provided key word and/or search strategy  x justified publication restrictions | |  |  |   x  | | Yes  Partial Yes No |  |
|  | (e.g. language) | |  |  |  | |  |  |
|  | Comments: Recognition of language restrictions outlined in the limitations section (p11). | | | | | | |  |
|  | **5. Did the review authors perform study selection in duplicate?** | | | |  | | |  |
|  | For Yes, either ONE of the following:  x at least two reviewers independently agreed on selection of eligible studies and achieved consensus on which studies to include   OR two reviewers selected a sample of eligible studies and achieved good agreement (at least 80 percent), with the remainder selected by one reviewer. | | | | x | | Yes No |  |
|  | Comments: Permanent products of all stages of the selection were developed by the first researcher and these were all reviewed by the second researcher. | | | | | | |  |
| **6. Did the review authors perform data extraction in duplicate?** | | | | | | | | |
| For Yes, either ONE of the following:   at least two reviewers achieved consensus on which data to extract from included studies  x OR two reviewers extracted data from a sample of eligible studies and achieved good agreement (at least 80 percent), with the remainder extracted by one reviewer. | | | | | | x Yes   No | | |
| Comments: 33% of extraction was reviewed by the 2^nd^ researcher which received 100% agreement | | | | | | | | |
| **7. Did the review authors provide a list of excluded studies and justify the exclusions?** | | | | | | | | |
|  | | For Partial Yes:   provided a list of all potentially relevant studies that were read in full-text form but excluded from the review | | For Yes, must also have:   Justified the exclusion from the review of each potentially relevant study | |  Yes   Partial Yes  x No | | |
|  | | Comments: A summary of explanations provided for exclusion but not specified by full text reviewed study | | | | | | |
| **8. Did the review authors describe the included studies in adequate detail?** | | | | | | | | |
|  | | For Partial Yes (ALL the following):  x described populations  x described interventions  n/a described comparators  x described outcomes  x described research designs | | For Yes, should also have ALL the following:  x described population in detail   described intervention in detail (including doses where relevant)   described comparator in detail (including doses where relevant)   described study’s setting   timeframe for follow-up | |  Yes  x Partial Yes   No | | |
|  | | Comments: The review provides detailed information of the 6 studies included. | | | | | | |
| **9. Did the review authors use a satisfactory technique for assessing the risk of bias (RoB) in individual studies that were included in the review?** | | | | | | | | |
|  | | **RCTs**  For Partial Yes, must have assessed RoB from   unconcealed allocation, *and*   lack of blinding of patients and assessors when assessing outcomes (unnecessary for objective outcomes such as all-cause mortality) | | For Yes, must also have assessed RoB from:   allocation sequence that was not truly random, *and*   selection of the reported result from among multiple measurements or analyses of a specified outcome | |  Yes   Partial Yes   No   Includes only NRSI | | |
|  | | **NRSI**  For Partial Yes, must have assessed RoB:   from confounding, *and*   from selection bias | | For Yes, must also have assessed RoB:   methods used to ascertain exposures and outcomes, *and*   selection of the reported result from among multiple measurements or analyses of a specified | |  Yes   Partial Yes  x No  Includes only RCTs | | |
| Comments: This is a scoping study, and in those authors do not typically assess the quality of included studies | | | | | | | | |

| **10. Did the review authors report outcomes on the sources of funding for the studies included in the review?** | | | | | | |
| --- | --- | --- | --- | --- | --- | --- |
| For Yes:   Must have reported on the sources of funding for individual studies included in the review.  Note: Reporting that the reviewers looked for this information but it was not reported by study authors also qualifies. | | | |  Yes  x No |  |  |
| Comments: None | | | |  |  |  |
| **11. If meta-analysis was performed did the review authors use appropriate methods for statistical combination of results?** | | | | | | |
|  | **RCTs**  For Yes:   The authors justified combining the data in a meta-analysis   AND they used an appropriate weighted technique to combine study results and adjusted for heterogeneity if present.   AND investigated the causes of any heterogeneity | |  Yes   No  x No meta-analysis conducted | | |  |
|  | **For NRSI**  For Yes:   The authors justified combining the data in a meta-analysis   AND they used an appropriate weighted technique to combine study results, adjusting for heterogeneity if present   AND they statistically combined effect estimates from NRSI that were adjusted for confounding, rather than combining raw data, or justified combining raw data when adjusted effect estimates were not available   AND they reported separate summary estimates for RCTs and NRSI separately when both were included in the review | |  Yes   No  x No meta-analysis conducted | | |  |
|  | Comments: None | | | | |  |
| **12. If meta-analysis was performed, did the review authors assess the potential impact of RoB in individual studies on the results of the meta-analysis or other evidence synthesis?** | | | | | | |
|  | For Yes:   included only low risk of bias RCTs   OR, if the pooled estimate was based on RCTs and/or NRSI at variable RoB, the authors performed analyses to investigate possible impact of RoB on summary estimates of effect. | |  Yes   No   x No meta-analysis conducted | | |  |
|  | Comments: None | | | | |  |
| **13. Did the review authors account for RoB in individual studies when interpreting/ discussing the results of the review?** | | | | | | |
|  | For Yes:   included only low risk of bias RCTs   OR, if RCTs with moderate or high RoB, or NRSI were included the review provided a discussion of the likely impact of RoB on the results | |  Yes  x No | | |  |
|  | Comments: This was a scoping study, and in those authors do not typically assess the quality of included studies. | | | | |  |
| **14. Did the review authors provide a satisfactory explanation for, and discussion of, any heterogeneity observed in the results of the review?** | | | | | | |
|  | For Yes:   There was no significant heterogeneity in the results   OR if heterogeneity was present the authors performed an investigation of sources of any heterogeneity in the results and discussed the impact of this on the results of the review | | x Yes   No | | |  |
|  | Comments: Heterogeneity is not discussed explicitly, but the variety of types of studies is discussed throughout. | | | | |  |
| **15. If they performed quantitative synthesis did the review authors carry out an adequate investigation of publication bias (small study bias) and discuss its likely impact on the results of the review?** | | | | | | |
|  | For Yes:   performed graphical or statistical tests for publication bias and discussed the likelihood and magnitude of impact of publication bias | |  Yes   No   xNo meta-analysis conducted | | |  |
|  | Comments: None | | | | |  |
| **16. Did the review authors report any potential sources of conflict of interest, including any funding they received for conducting the review?** | | | | | | |
|  | For Yes:  x The authors reported no competing interests OR   The authors described their funding sources and how they managed potential conflicts of interest | x Yes   No | | | |  |
|  | Comments: The authors state no conflict of interest or disclosures to declare. | | | | |  |

# Study 16 - Labiris et al

| **1. Did the research questions and inclusion criteria for the review include the components of PICO?** | | | | | | | | |
| --- | --- | --- | --- | --- | --- | --- | --- | --- |
| For Yes:  x Population  x Intervention  x Comparator group  x Outcome | | | Optional (recommended)  x Timeframe for follow-up | | x | | Yes No |  |
| Comments: None. | | | | | | | |  |
| **2. Did the report of the review contain an explicit statement that the review methods were established prior to the conduct of the review and did the report justify any significant deviations from the protocol?** | | | | | | | | |
|  | For Partial Yes:  The authors state that they had a written protocol or guide that included ALL the following:  x review question(s)  x a search strategy  x inclusion/exclusion criteria   a risk of bias assessment | | For Yes:  As for partial yes, plus the protocol should be registered and should also have specified:   a meta-analysis/synthesis plan, if appropriate, *and*   a plan for investigating causes of heterogeneity   justification for any deviations from the protocol | |   x | | Yes  Partial Yes  No |  |
|  | Comments: Authors did not perform RoB. | | | | | | |  |
| **3. Did the review authors explain their selection of the study designs for inclusion in the review?** | | | | | | | | |
|  | For Yes, the review should satisfy ONE of the following:   *Explanation for* including only RCTs   OR *Explanation for* including only NRSI  x OR *Explanation for* including both RCTs and NRSI | | | | x | | Yes No |  |
|  | Comments: None. | | | | | | |  |
| **4. Did the review authors use a comprehensive literature search strategy?** | | | | | | | | |
|  | For Partial Yes (all the following): | | For Yes, should also have (all the following):   searched the reference lists / bibliographies of included studies   searched trial/study registries   included/consulted content experts in the field   where relevant, searched for grey literature   conducted search within 24 months of completion of the review | |  | |  |  |
|  | x searched at least 2 databases (relevant to research question)  x provided key word and/or search strategy  x justified publication restrictions | |  |  |   x  | | Yes  Partial Yes No |  |
|  | (e.g. language) | |  |  |  | |  |  |
|  | Comments: Explain the reason for restrictions. | | | | | | |  |
|  | **5. Did the review authors perform study selection in duplicate?** | | | |  | | |  |
|  | For Yes, either ONE of the following:   at least two reviewers independently agreed on selection of eligible studies and achieved consensus on which studies to include   OR two reviewers selected a sample of eligible studies and achieved good agreement (at least 80 percent), with the remainder selected by one reviewer. | | | | x | | Yes NoN/A |  |
|  | Comments: None. | | | | | | |  |
| **6. Did the review authors perform data extraction in duplicate?** | | | | | | | | |
| For Yes, either ONE of the following:   at least two reviewers achieved consensus on which data to extract from included studies   OR two reviewers extracted data from a sample of eligible studies and achieved good agreement (at least 80 percent), with the remainder extracted by one reviewer. | | | | | |  Yes  x No N/A | | |
| Comments: Not available. | | | | | | | | |
| **7. Did the review authors provide a list of excluded studies and justify the exclusions?** | | | | | | | | |
|  | | For Partial Yes:   provided a list of all potentially relevant studies that were read in full-text form but excluded from the review | | For Yes, must also have:   Justified the exclusion from the review of each potentially relevant study | |  Yes   Partial Yes  x No | | |
|  | | Comments: Not available. | | | | | | |
| **8. Did the review authors describe the included studies in adequate detail?** | | | | | | | | |
|  | | For Partial Yes (ALL the following):  x described populations  x described interventions  x described comparators  x described outcomes  x described research designs | | For Yes, should also have ALL the following:   described population in detail   described intervention in detail (including doses where relevant)   described comparator in detail (including doses where relevant)   described study’s setting   timeframe for follow-up | |  Yes  x Partial Yes   No | | |
|  | | Comments: None. | | | | | | |
| **9. Did the review authors use a satisfactory technique for assessing the risk of bias (RoB) in individual studies that were included in the review?** | | | | | | | | |
|  | | **RCTs**  For Partial Yes, must have assessed RoB from   unconcealed allocation, *and*   lack of blinding of patients and assessors when assessing outcomes (unnecessary for objective outcomes such as all-cause mortality) | | For Yes, must also have assessed RoB from:   allocation sequence that was not truly random, *and*   selection of the reported result from among multiple measurements or analyses of a specified outcome | |  Yes   Partial Yes  x No   Includes only NRSI | | |
|  | | **NRSI**  For Partial Yes, must have assessed RoB:   from confounding, *and*   from selection bias | | For Yes, must also have assessed RoB:   methods used to ascertain exposures and outcomes, *and*   selection of the reported result from among multiple measurements or analyses of a specified | |  Yes   Partial Yes  x No  Includes only RCTs | | |
| Comments: None. | | | | | | | | |

| **10. Did the review authors report outcomes on the sources of funding for the studies included in the review?** | | | | | | |
| --- | --- | --- | --- | --- | --- | --- |
| For Yes:   Must have reported on the sources of funding for individual studies included in the review.  Note: Reporting that the reviewers looked for this information but it was not reported by study authors also qualifies. | | | |  Yes  x No |  |  |
| Comments: None. | | | |  |  |  |
| **11. If meta-analysis was performed did the review authors use appropriate methods for statistical combination of results?** | | | | | | |
|  | **RCTs**  For Yes:   The authors justified combining the data in a meta-analysis   AND they used an appropriate weighted technique to combine study results and adjusted for heterogeneity if present.   AND investigated the causes of any heterogeneity | |  Yes   No  x No meta-analysis conducted | | |  |
|  | **For NRSI**  For Yes:   The authors justified combining the data in a meta-analysis   AND they used an appropriate weighted technique to combine study results, adjusting for heterogeneity if present   AND they statistically combined effect estimates from NRSI that were adjusted for confounding, rather than combining raw data, or justified combining raw data when adjusted effect estimates were not available   AND they reported separate summary estimates for RCTs and NRSI separately when both were included in the review | |  Yes   No  x No meta-analysis conducted | | |  |
|  | Comments: None. | | | | |  |
| **12. If meta-analysis was performed, did the review authors assess the potential impact of RoB in individual studies on the results of the meta-analysis or other evidence synthesis?** | | | | | | |
|  | For Yes:   included only low risk of bias RCTs   OR, if the pooled estimate was based on RCTs and/or NRSI at variable RoB, the authors performed analyses to investigate possible impact of RoB on summary estimates of effect. | |  Yes   No  x No meta-analysis conducted | | |  |
|  | Comments: None. | | | | |  |
| **13. Did the review authors account for RoB in individual studies when interpreting/ discussing the results of the review?** | | | | | | |
|  | For Yes:   included only low risk of bias RCTs   OR, if RCTs with moderate or high RoB, or NRSI were included the review provided a discussion of the likely impact of RoB on the results | |  Yes  x No | | |  |
|  | Comments: None | | | | |  |
| **14. Did the review authors provide a satisfactory explanation for, and discussion of, any heterogeneity observed in the results of the review?** | | | | | | |
|  | For Yes:   There was no significant heterogeneity in the results   OR if heterogeneity was present the authors performed an investigation of sources of any heterogeneity in the results and discussed the impact of this on the results of the review | |  Yes  x No | | |  |
|  | Comments: None | | | | |  |
| **15. If they performed quantitative synthesis did the review authors carry out an adequate investigation of publication bias (small study bias) and discuss its likely impact on the results of the review?** | | | | | | |
|  | For Yes:   performed graphical or statistical tests for publication bias and discussed the likelihood and magnitude of impact of publication bias | |  Yes   No  x No meta-analysis conducted | | |  |
|  | Comments: None | | | | |  |
| **16. Did the review authors report any potential sources of conflict of interest, including any funding they received for conducting the review?** | | | | | | |
|  | For Yes:  x The authors reported no competing interests OR   The authors described their funding sources and how they managed potential conflicts of interest | x Yes   No | | | |  |
|  | Comments: None. | | | | |  |

# Study 17 - Maresca et al

| **1. Did the research questions and inclusion criteria for the review include the components of PICO?** | | | | | | | | |
| --- | --- | --- | --- | --- | --- | --- | --- | --- |
| For Yes:  x Population  x Intervention  x Comparator group  x Outcome | | | Optional (recommended)   Timeframe for follow-up | | x | | Yes No |  |
| Comments: | | | | | | | |  |
| **2. Did the report of the review contain an explicit statement that the review methods were established prior to the conduct of the review and did the report justify any significant deviations from the protocol?** | | | | | | | | |
|  | For Partial Yes:  The authors state that they had a written protocol or guide that included ALL the following:  x review question(s)  x a search strategy  x inclusion/exclusion criteria   a risk of bias assessment | | For Yes:  As for partial yes, plus the protocol should be registered and should also have specified:   a meta-analysis/synthesis plan, if appropriate, *and*   a plan for investigating causes of heterogeneity   justification for any deviations from the protocol | |   x | | Yes  Partial Yes  No |  |
|  | Comments: | | | | | | |  |
| **3. Did the review authors explain their selection of the study designs for inclusion in the review?** | | | | | | | | |
|  | For Yes, the review should satisfy ONE of the following:   *Explanation for* including only RCTs   OR *Explanation for* including only NRSI  x OR *Explanation for* including both RCTs and NRSI | | | | x | | Yes No |  |
|  | Comments: | | | | | | |  |
| **4. Did the review authors use a comprehensive literature search strategy?** | | | | | | | | |
|  | For Partial Yes (all the following): | | For Yes, should also have (all the following):   searched the reference lists / bibliographies of included studies   searched trial/study registries   included/consulted content experts in the field   where relevant, searched for grey literature   conducted search within 24 months of completion of the review | |  | |  |  |
|  | x searched at least 2 databases (relevant to research question)  x provided key word and/or search strategy   justified publication restrictions | |  |  |     x | | Yes  Partial Yes No |  |
|  | (e.g. language) | |  |  |  | |  |  |
|  | Comments: | | | | | | |  |
|  | **5. Did the review authors perform study selection in duplicate?** | | | |  | | |  |
|  | For Yes, either ONE of the following:   at least two reviewers independently agreed on selection of eligible studies and achieved consensus on which studies to include   OR two reviewers selected a sample of eligible studies and achieved good agreement (at least 80 percent), with the remainder selected by one reviewer. | | | | x | | Yes No N/A |  |
|  | Comments: | | | | | | |  |
| **6. Did the review authors perform data extraction in duplicate?** | | | | | | | | |
| For Yes, either ONE of the following:   at least two reviewers achieved consensus on which data to extract from included studies   OR two reviewers extracted data from a sample of eligible studies and achieved good agreement (at least 80 percent), with the remainder extracted by one reviewer. | | | | | |  Yes  x No N/A | | |
| Comments: | | | | | | | | |
| **7. Did the review authors provide a list of excluded studies and justify the exclusions?** | | | | | | | | |
|  | | For Partial Yes:   provided a list of all potentially relevant studies that were read in full-text form but excluded from the review | | For Yes, must also have:   Justified the exclusion from the review of each potentially relevant study | |  Yes   Partial Yes  x No | | |
|  | | Comments: | | | | | | |
| **8. Did the review authors describe the included studies in adequate detail?** | | | | | | | | |
|  | | For Partial Yes (ALL the following):  x described populations  x described interventions  x described comparators  x described outcomes  x described research designs | | For Yes, should also have ALL the following:  x described population in detail   described intervention in detail (including doses where relevant)   described comparator in detail (including doses where relevant)  x described study’s setting   timeframe for follow-up | |  Yes  x Partial Yes   No | | |
|  | | Comments: | | | | | | |
| **9. Did the review authors use a satisfactory technique for assessing the risk of bias (RoB) in individual studies that were included in the review?** | | | | | | | | |
|  | | **RCTs**  For Partial Yes, must have assessed RoB from   unconcealed allocation, *and*   lack of blinding of patients and assessors when assessing outcomes (unnecessary for objective outcomes such as all-cause mortality) | | For Yes, must also have assessed RoB from:   allocation sequence that was not truly random, *and*   selection of the reported result from among multiple measurements or analyses of a specified outcome | |  Yes   Partial Yes  x No   Includes only NRSI | | |
|  | | **NRSI**  For Partial Yes, must have assessed RoB:   from confounding, *and*   from selection bias | | For Yes, must also have assessed RoB:   methods used to ascertain exposures and outcomes, *and*   selection of the reported result from among multiple measurements or analyses of a specified | |  Yes   Partial Yes  x No  Includes only RCTs | | |
| Comments: | | | | | | | | |

| **10. Did the review authors report outcomes on the sources of funding for the studies included in the review?** | | | | | | |
| --- | --- | --- | --- | --- | --- | --- |
| For Yes:   Must have reported on the sources of funding for individual studies included in the review.  Note: Reporting that the reviewers looked for this information but it was not reported by study authors also qualifies. | | | |  Yes  x No |  |  |
| Comments: | | | |  |  |  |
| **11. If meta-analysis was performed did the review authors use appropriate methods for statistical combination of results?** | | | | | | |
|  | **RCTs**  For Yes:   The authors justified combining the data in a meta-analysis   AND they used an appropriate weighted technique to combine study results and adjusted for heterogeneity if present.   AND investigated the causes of any heterogeneity | |  Yes   No  x No meta-analysis conducted | | |  |
|  | **For NRSI**  For Yes:   The authors justified combining the data in a meta-analysis   AND they used an appropriate weighted technique to combine study results, adjusting for heterogeneity if present   AND they statistically combined effect estimates from NRSI that were adjusted for confounding, rather than combining raw data, or justified combining raw data when adjusted effect estimates were not available   AND they reported separate summary estimates for RCTs and NRSI separately when both were included in the review | |  Yes   No  x No meta-analysis conducted | | |  |
|  | Comments: | | | | |  |
| **12. If meta-analysis was performed, did the review authors assess the potential impact of RoB in individual studies on the results of the meta-analysis or other evidence synthesis?** | | | | | | |
|  | For Yes:   included only low risk of bias RCTs   OR, if the pooled estimate was based on RCTs and/or NRSI at variable RoB, the authors performed analyses to investigate possible impact of RoB on summary estimates of effect. | |  Yes   No  x No meta-analysis conducted | | |  |
|  | Comments: | | | | |  |
| **13. Did the review authors account for RoB in individual studies when interpreting/ discussing the results of the review?** | | | | | | |
|  | For Yes:   included only low risk of bias RCTs   OR, if RCTs with moderate or high RoB, or NRSI were included the review provided a discussion of the likely impact of RoB on the results | |  Yes  x No | | |  |
|  | Comments: | | | | |  |
| **14. Did the review authors provide a satisfactory explanation for, and discussion of, any heterogeneity observed in the results of the review?** | | | | | | |
|  | For Yes:   There was no significant heterogeneity in the results   OR if heterogeneity was present the authors performed an investigation of sources of any heterogeneity in the results and discussed the impact of this on the results of the review | |  Yes  x No | | |  |
|  | Comments: | | | | |  |
| **15. If they performed quantitative synthesis did the review authors carry out an adequate investigation of publication bias (small study bias) and discuss its likely impact on the results of the review?** | | | | | | |
|  | For Yes:   performed graphical or statistical tests for publication bias and discussed the likelihood and magnitude of impact of publication bias | |  Yes   No  x No meta-analysis conducted | | |  |
|  | Comments: | | | | |  |
| **16. Did the review authors report any potential sources of conflict of interest, including any funding they received for conducting the review?** | | | | | | |
|  | For Yes:   The authors reported no competing interests OR   The authors described their funding sources and how they managed potential conflicts of interest |  Yes  x No | | | |  |
|  | Comments: | | | | |  |

# Study 18 – Martin et al

| **1. Did the research questions and inclusion criteria for the review include the components of PICO?** | | | | | | | | |
| --- | --- | --- | --- | --- | --- | --- | --- | --- |
| For Yes:  x Population  x Intervention  n/a Comparator group  x  Outcome | | | Optional (recommended)   Timeframe for follow-up | | x | | Yes No |  |
| Comments: As this was a systematic review C-comparison not applicable | | | | | | | |  |
| **2. Did the report of the review contain an explicit statement that the review methods were established prior to the conduct of the review and did the report justify any significant deviations from the protocol?** | | | | | | | | |
|  | For Partial Yes:  The authors state that they had a written protocol or guide that included ALL the following:  x review question(s)  x a search strategy  x inclusion/exclusion criteria  n/a a risk of bias assessment | | For Yes:  As for partial yes, plus the protocol should be registered and should also have specified:   a meta-analysis/synthesis plan, if appropriate, *and*   a plan for investigating causes of heterogeneity   justification for any deviations from the protocol | |   x  | | Yes  Partial Yes  No |  |
|  | Comments: Considerable detail provided of the research protocol. | | | | | | |  |
| **3. Did the review authors explain their selection of the study designs for inclusion in the review?** | | | | | | | | |
|  | For Yes, the review should satisfy ONE of the following:   *Explanation for* including only RCTs   OR *Explanation for* including only NRSI   OR *Explanation for* including both RCTs and NRSI | | | | x  | | Yes No |  |
|  | Comments: An explicit explanation is not provided, but the authors included all types of study designs. | | | | | | |  |
| **4. Did the review authors use a comprehensive literature search strategy?** | | | | | | | | |
|  | For Partial Yes (all the following): | | For Yes, should also have (all the following):   searched the reference lists / bibliographies of included studies   searched trial/study registries   included/consulted content experts in the field   where relevant, searched for grey literature   conducted search within 24 months of completion of the review | |  | |  |  |
|  | x searched at least 2 databases (relevant to research question)  x provided key word and/or search strategy   justified publication restrictions | |  |  |    | | Yes  Partial Yes No |  |
|  | (e.g. language) | |  |  |  | |  |  |
|  | Comments: Recognition of language restrictions outlined in the limitations section (p109). | | | | | | |  |
|  | **5. Did the review authors perform study selection in duplicate?** | | | |  | | |  |
|  | For Yes, either ONE of the following:   at least two reviewers independently agreed on selection of eligible studies and achieved consensus on which studies to include   OR two reviewers selected a sample of eligible studies and achieved good agreement (at least 80 percent), with the remainder selected by one reviewer. | | | | x  | | Yes No |  |
|  | Comments: Not explicitly stated, but implied by their stated following of the general principles recommended in PRISMA guidance | | | | | | |  |
| **6. Did the review authors perform data extraction in duplicate?** | | | | | | | | |
| For Yes, either ONE of the following:   at least two reviewers achieved consensus on which data to extract from included studies  x OR two reviewers extracted data from a sample of eligible studies and achieved good agreement (at least 80 percent), with the remainder extracted by one reviewer. | | | | | | x Yes   No | | |
| Comments: None | | | | | | | | |
| **7. Did the review authors provide a list of excluded studies and justify the exclusions?** | | | | | | | | |
|  | | For Partial Yes:   provided a list of all potentially relevant studies that were read in full-text form but excluded from the review | | For Yes, must also have:   Justified the exclusion from the review of each potentially relevant study | |  Yes   Partial Yes  x No | | |
|  | | Comments: A summary of justification for exclusion is provided | | | | | | |
| **8. Did the review authors describe the included studies in adequate detail?** | | | | | | | | |
|  | | For Partial Yes (ALL the following):  x described populations  x described interventions  n/a described comparators  x described outcomes  x described research designs | | For Yes, should also have ALL the following:  x described population in detail  x described intervention in detail (including doses where relevant)  n/a described comparator in detail (including doses where relevant)  x described study’s setting  x timeframe for follow-up | | x Yes   Partial Yes   No | | |
|  | | Comments: Extensive information provided in the extraction table for each study. | | | | | | |
| **9. Did the review authors use a satisfactory technique for assessing the risk of bias (RoB) in individual studies that were included in the review?** | | | | | | | | |
|  | | **RCTs**  For Partial Yes, must have assessed RoB from   unconcealed allocation, *and*   lack of blinding of patients and assessors when assessing outcomes (unnecessary for objective outcomes such as all-cause mortality) | | For Yes, must also have assessed RoB from:   allocation sequence that was not truly random, *and*   selection of the reported result from among multiple measurements or analyses of a specified outcome | |  Yes   Partial Yes  x No   Includes only NRSI | | |
|  | | **NRSI**  For Partial Yes, must have assessed RoB:   from confounding, *and*   from selection bias | | For Yes, must also have assessed RoB:   methods used to ascertain exposures and outcomes, *and*   selection of the reported result from among multiple measurements or analyses of a specified | |  Yes   Partial Yes  x No  Includes only RCTs | | |
| Comments: This is a systematic review, and in these authors do not typically assess the quality of included studies. | | | | | | | | |

| **10. Did the review authors report outcomes on the sources of funding for the studies included in the review?** | | | | | | |
| --- | --- | --- | --- | --- | --- | --- |
| For Yes:   Must have reported on the sources of funding for individual studies included in the review.  Note: Reporting that the reviewers looked for this information but it was not reported by study authors also qualifies. | | | |  Yes  x No |  |  |
| Comments: Some cost-effectiveness analysis included but funding information was not extracted. | | | |  |  |  |
| **11. If meta-analysis was performed did the review authors use appropriate methods for statistical combination of results?** | | | | | | |
|  | **RCTs**  For Yes:   The authors justified combining the data in a meta-analysis   AND they used an appropriate weighted technique to combine study results and adjusted for heterogeneity if present.   AND investigated the causes of any heterogeneity | |  Yes   No  x No meta-analysis conducted | | |  |
|  | **For NRSI**  For Yes:   The authors justified combining the data in a meta-analysis   AND they used an appropriate weighted technique to combine study results, adjusting for heterogeneity if present   AND they statistically combined effect estimates from NRSI that were adjusted for confounding, rather than combining raw data, or justified combining raw data when adjusted effect estimates were not available   AND they reported separate summary estimates for RCTs and NRSI separately when both were included in the review | |  Yes   No  x No meta-analysis conducted | | |  |
|  | Comments: Not applicable | | | | |  |
| **12. If meta-analysis was performed, did the review authors assess the potential impact of RoB in individual studies on the results of the meta-analysis or other evidence synthesis?** | | | | | | |
|  | For Yes:   included only low risk of bias RCTs   OR, if the pooled estimate was based on RCTs and/or NRSI at variable RoB, the authors performed analyses to investigate possible impact of RoB on summary estimates of effect. | |  Yes   No   x No meta-analysis conducted | | |  |
|  | Comments: Not applicable | | | | |  |
| **13. Did the review authors account for RoB in individual studies when interpreting/ discussing the results of the review?** | | | | | | |
|  | For Yes:   included only low risk of bias RCTs   OR, if RCTs with moderate or high RoB, or NRSI were included the review provided a discussion of the likely impact of RoB on the results | |  Yes   xNo | | |  |
|  | Comments: This is a systematic review and risk of bias is not usually assessed. | | | | |  |
| **14. Did the review authors provide a satisfactory explanation for, and discussion of, any heterogeneity observed in the results of the review?** | | | | | | |
|  | For Yes:   There was no significant heterogeneity in the results   OR if heterogeneity was present the authors performed an investigation of sources of any heterogeneity in the results and discussed the impact of this on the results of the review | | x Yes   No | | |  |
|  | Comments: The observed heterogeneity of interventions and patient groups is identified as a limitation in drawing robust conclusions. The variety of types of studies is discussed throughout. | | | | |  |
| **15. If they performed quantitative synthesis did the review authors carry out an adequate investigation of publication bias (small study bias) and discuss its likely impact on the results of the review?** | | | | | | |
|  | For Yes:   performed graphical or statistical tests for publication bias and discussed the likelihood and magnitude of impact of publication bias | |  Yes   No   xNo meta-analysis conducted | | |  |
|  | Comments: Not applicable | | | | |  |
| **16. Did the review authors report any potential sources of conflict of interest, including any funding they received for conducting the review?** | | | | | | |
|  | For Yes:  x The authors reported no competing interests OR  x The authors described their funding sources and how they managed potential conflicts of interest | x Yes   No | | | |  |
|  | Comments: The paper states *The authors declare that there is no actual or potential conflict of interest including any financial, personal or other relationships with other people or organizations within three years of beginning the submitted work that could inappropriately influence, or be perceived to influence, their work.* | | | | |  |

# Study 19 - McFarland et al

| **1. Did the research questions and inclusion criteria for the review include the components of PICO?** | | | | | | | | |
| --- | --- | --- | --- | --- | --- | --- | --- | --- |
| For Yes:  x  Population  x Intervention  x Comparator group  x Outcome | | | Optional (recommended)   Timeframe for follow-up | | x | | Yes No |  |
| Comments: Meta-analysis undertaken to determine the cost effectiveness of telehealth interventions in the community compared to usual care and its impact on quality of life. | | | | | | | |  |
| **2. Did the report of the review contain an explicit statement that the review methods were established prior to the conduct of the review and did the report justify any significant deviations from the protocol?** | | | | | | | | |
|  | For Partial Yes:  The authors state that they had a written protocol or guide that included ALL the following:  x review question(s)  x a search strategy  x inclusion/exclusion criteria  x a risk of bias assessment | | For Yes:  As for partial yes, plus the protocol should be registered and should also have specified:  x a meta-analysis/synthesis plan, if appropriate, *and*  x a plan for investigating causes of heterogeneity   justification for any deviations from the protocol | | x   | | Yes  Partial Yes  No |  |
|  | Comments: Extensive explanation of review methodology provided including meta-analysis | | | | | | |  |
| **3. Did the review authors explain their selection of the study designs for inclusion in the review?** | | | | | | | | |
|  | For Yes, the review should satisfy ONE of the following:   *Explanation for* including only RCTs   OR *Explanation for* including only NRSI   OR *Explanation for* including both RCTs and NRSI | | | | x  | | Yes No |  |
|  | Comments: Detailed explanation provided. | | | | | | |  |
| **4. Did the review authors use a comprehensive literature search strategy?** | | | | | | | | |
|  | For Partial Yes (all the following): | | For Yes, should also have (all the following):   searched the reference lists / bibliographies of included studies   searched trial/study registries   included/consulted content experts in the field   where relevant, searched for grey literature   conducted search within 24 months of completion of the review | |  | |  |  |
|  | x searched at least 2 databases (relevant to research question)  x provided key word and/or search strategy  x justified publication restrictions | |  |  |   x  | | Yes  Partial Yes No |  |
|  | (e.g. language) | |  |  |  | |  |  |
|  | Comments: No language restrictions in search strategy, nevertheless all papers selected were in English, this is acknowledged as a potential bias of the findings (p16) | | | | | | |  |
|  | **5. Did the review authors perform study selection in duplicate?** | | | |  | | |  |
|  | For Yes, either ONE of the following:   at least two reviewers independently agreed on selection of eligible studies and achieved consensus on which studies to include  x OR two reviewers selected a sample of eligible studies and achieved good agreement (at least 80 percent), with the remainder selected by one reviewer. | | | | x  | | Yes No |  |
|  | Comments: A review process is indicated with at least 2 researchers but details are not specified. | | | | | | |  |
| **6. Did the review authors perform data extraction in duplicate?** | | | | | | | | |
| For Yes, either ONE of the following:   at least two reviewers achieved consensus on which data to extract from included studies  x OR two reviewers extracted data from a sample of eligible studies and achieved good agreement (at least 80 percent), with the remainder extracted by one reviewer. | | | | | | x Yes   No | | |
| Comments: A review process is indicated with at least 2 researchers but details are not specified. | | | | | | | | |
| **7. Did the review authors provide a list of excluded studies and justify the exclusions?** | | | | | | | | |
|  | | For Partial Yes:   provided a list of all potentially relevant studies that were read in full-text form but excluded from the review | | For Yes, must also have:   Justified the exclusion from the review of each potentially relevant study | |  Yes   Partial Yes  x No | | |
|  | | Comments: A summary of justification for exclusion is provided | | | | | | |
| **8. Did the review authors describe the included studies in adequate detail?** | | | | | | | | |
|  | | For Partial Yes (ALL the following):  x described populations  x described interventions  x described comparators  x described outcomes  x described research designs | | For Yes, should also have ALL the following:  x described population in detail  x described intervention in detail (including doses where relevant)   described comparator in detail (including doses where relevant)  x described study’s setting  x timeframe for follow-up | | x Yes   Partial Yes   No | | |
|  | | Comments: Extensive information provided relevant to the research question for each study. | | | | | | |
| **9. Did the review authors use a satisfactory technique for assessing the risk of bias (RoB) in individual studies that were included in the review?** | | | | | | | | |
|  | | **RCTs**  For Partial Yes, must have assessed RoB from  x unconcealed allocation, *and*  x lack of blinding of patients and assessors when assessing outcomes (unnecessary for objective outcomes such as all-cause mortality) | | For Yes, must also have assessed RoB from:   allocation sequence that was not truly random, *and*   selection of the reported result from among multiple measurements or analyses of a specified outcome | |  Yes  x Partial Yes   No   Includes only NRSI | | |
|  | | **NRSI**  For Partial Yes, must have assessed RoB:  x from confounding, *and*  x from selection bias | | For Yes, must also have assessed RoB:   methods used to ascertain exposures and outcomes, *and*   selection of the reported result from among multiple measurements or analyses of a specified | |  Yes  x Partial Yes   No  Includes only RCTs | | |
| Comments: The paper states *The Cochrane tool for assessing the risk of bias was used to assess the bias of random sequence generation, allocation concealment, blinding of participants and personnel, blinding of outcome assessment, incomplete data outcome and selective reporting of quantitative studies. A Critical Appraisal Skills Programme Checklist was used to assess qualitative research.* | | | | | | | | |

| **10. Did the review authors report outcomes on the sources of funding for the studies included in the review?** | | | | | | |
| --- | --- | --- | --- | --- | --- | --- |
| For Yes:   Must have reported on the sources of funding for individual studies included in the review.  Note: Reporting that the reviewers looked for this information but it was not reported by study authors also qualifies. | | | |  Yes  x No |  |  |
| Comments: None | | | |  |  |  |
| **11. If meta-analysis was performed did the review authors use appropriate methods for statistical combination of results?** | | | | | | |
|  | **RCTs**  For Yes:  x The authors justified combining the data in a meta-analysis  x AND they used an appropriate weighted technique to combine study results and adjusted for heterogeneity if present.  x AND investigated the causes of any heterogeneity | | x Yes   No   No meta-analysis conducted | | |  |
|  | **For NRSI**  For Yes:  x The authors justified combining the data in a meta-analysis  x AND they used an appropriate weighted technique to combine study results, adjusting for heterogeneity if present   AND they statistically combined effect estimates from NRSI that were adjusted for confounding, rather than combining raw data, or justified combining raw data when adjusted effect estimates were not available  x AND they reported separate summary estimates for RCTs and NRSI separately when both were included in the review | | x Yes   No   No meta-analysis conducted | | |  |
|  | Comments: Considerable information provided on the methodology for meta-analysis comprising 7 RCTs. 2 qualitative studies were analyzed in narrative. | | | | |  |
| **12. If meta-analysis was performed, did the review authors assess the potential impact of RoB in individual studies on the results of the meta-analysis or other evidence synthesis?** | | | | | | |
|  | For Yes:  x included only low risk of bias RCTs   OR, if the pooled estimate was based on RCTs and/or NRSI at variable RoB, the authors performed analyses to investigate possible impact of RoB on summary estimates of effect. | | x Yes   No   No meta-analysis conducted | | |  |
|  | Comments: The paper states *The Cochrane tool for assessing the risk of bias was used to assess the bias of random sequence generation, allocation concealment, blinding of participants and personnel, blinding of outcome assessment, incomplete data outcome and selective reporting of quantitative studies. A Critical Appraisal Skills Programme Checklist was used to assess qualitative research.* | | | | |  |
| **13. Did the review authors account for RoB in individual studies when interpreting/ discussing the results of the review?** | | | | | | |
|  | For Yes:  x included only low risk of bias RCTs  x OR, if RCTs with moderate or high RoB, or NRSI were included the review provided a discussion of the likely impact of RoB on the results | | x Yes   No | | |  |
|  | Comments: The study states:  With respect to QoL - *Sensitivity analysis removing studies at high risk of bias was conducted on results from 6 months and 12 months but this had a negligible effect on the results*.  With respect to Anxiety - *These meta-analyses combine low-level bias studies only, yet are still statistically*  *not significant in terms of a difference in change in anxiety scores between telehealth and standard home care.*  With respect to depression - *Sensitivity analysis was conducted on data at 6 months and 12 months to combine low-level bias studies only to assess whether this influenced the results. Results from both sensitivity analyses were not significantly different to primary findings.* | | | | |  |
| **14. Did the review authors provide a satisfactory explanation for, and discussion of, any heterogeneity observed in the results of the review?** | | | | | | |
|  | For Yes:   There was no significant heterogeneity in the results  x OR if heterogeneity was present the authors performed an investigation of sources of any heterogeneity in the results and discussed the impact of this on the results of the review | | x Yes   No | | |  |
|  | Comments: Heterogeneity was assessed using the I2 statistic. | | | | |  |
| **15. If they performed quantitative synthesis did the review authors carry out an adequate investigation of publication bias (small study bias) and discuss its likely impact on the results of the review?** | | | | | | |
|  | For Yes:  x performed graphical or statistical tests for publication bias and discussed the likelihood and magnitude of impact of publication bias | |  Yes   No   No meta-analysis conducted | | |  |
|  | Comments: A Critical Appraisal Skills Programme Checklist was used to assess qualitative research bias. | | | | |  |
| **16. Did the review authors report any potential sources of conflict of interest, including any funding they received for conducting the review?** | | | | | | |
|  | For Yes:  x The authors reported no competing interests OR  x The authors described their funding sources and how they managed potential conflicts of interest | x Yes   No | | | |  |
|  | Comments: The authors state no potential conflicts of interest with respect to the research, authorship and/or publication of this article. Authors also state no financial support for the research,authorship and/or publication of this article. | | | | |  |

# Study 20 – Mold et al

| **1. Did the research questions and inclusion criteria for the review include the components of PICO?** | | | | | | | | |
| --- | --- | --- | --- | --- | --- | --- | --- | --- |
| For Yes:  x  Population  x Intervention  x Comparator group  x Outcome | | | Optional (recommended)   Timeframe for follow-up | |  | | Yes No |  |
| Comments: The study reports to follow Preferred Reporting Items for Systematic Reviews and Meta-Analyses guidelines. | | | | | | | |  |
| **2. Did the report of the review contain an explicit statement that the review methods were established prior to the conduct of the review and did the report justify any significant deviations from the protocol?** | | | | | | | | |
|  | For Partial Yes:  The authors state that they had a written protocol or guide that included ALL the following:  x review question(s)  x a search strategy  x inclusion/exclusion criteria  n/a a risk of bias assessment | | For Yes:  As for partial yes, plus the protocol should be registered and should also have specified:   a meta-analysis/synthesis plan, if appropriate, *and*  x a plan for investigating causes of heterogeneity   justification for any deviations from the protocol | |   x | | Yes  Partial Yes  No |  |
|  | Comments: This is a systematic review, and in those authors do not typically assess the qualify of included studies, though bias was considered in the selection process. | | | | | | |  |
| **3. Did the review authors explain their selection of the study designs for inclusion in the review?** | | | | | | | | |
|  | For Yes, the review should satisfy ONE of the following:   *Explanation for* including only RCTs   OR *Explanation for* including only NRSI   OR *Explanation for* including both RCTs and NRSI | | | | x | | Yes No |  |
|  | Comments: An explicit explanation is not provided, but the authors included all types of studies. | | | | | | |  |
| **4. Did the review authors use a comprehensive literature search strategy?** | | | | | | | | |
|  | For Partial Yes (all the following): | | For Yes, should also have (all the following):   searched the reference lists / bibliographies of included studies   searched trial/study registries  x included/consulted content experts in the field  x where relevant, searched for grey literature   conducted search within 24 months of completion of the review | |  | |  |  |
|  | x searched at least 2 databases (relevant to research question)  x provided key word and/or search strategy  x justified publication restrictions | |  |  |   x  | | Yes  Partial Yes No |  |
|  | (e.g. language) | |  |  |  | |  |  |
|  | Comments: Did not include studies that could not be translated. | | | | | | |  |
|  | **5. Did the review authors perform study selection in duplicate?** | | | |  | | |  |
|  | For Yes, either ONE of the following:  x at least two reviewers independently agreed on selection of eligible studies and achieved consensus on which studies to include   OR two reviewers selected a sample of eligible studies and achieved good agreement (at least 80 percent), with the remainder selected by one reviewer. | | | | x | | Yes No |  |
|  | Comments: None | | | | | | |  |
| **6. Did the review authors perform data extraction in duplicate?** | | | | | | | | |
| For Yes, either ONE of the following:  x at least two reviewers achieved consensus on which data to extract from included studies   OR two reviewers extracted data from a sample of eligible studies and achieved good agreement (at least 80 percent), with the remainder extracted by one reviewer. | | | | | | x Yes   No | | |
| Comments: None | | | | | | | | |
| **7. Did the review authors provide a list of excluded studies and justify the exclusions?** | | | | | | | | |
|  | | For Partial Yes:  x provided a list of all potentially relevant studies that were read in full-text form but excluded from the review | | For Yes, must also have:  x Justified the exclusion from the review of each potentially relevant study | | x Yes   Partial Yes   No | | |
|  | | Comments: A full list provided in Appendix 2 | | | | | | |
| **8. Did the review authors describe the included studies in adequate detail?** | | | | | | | | |
|  | | For Partial Yes (ALL the following):  x described populations  x described interventions  x described comparators  x described outcomes  x described research designs | | For Yes, should also have ALL the following:  x described population in detail  x described intervention in detail (including doses where relevant)  x described comparator in detail (including doses where relevant)  x described study’s setting   timeframe for follow-up | | x Yes   Partial Yes   No | | |
|  | | Comments: Comprehensive information provided on each study in Appendix 4 | | | | | | |
| **9. Did the review authors use a satisfactory technique for assessing the risk of bias (RoB) in individual studies that were included in the review?** | | | | | | | | |
|  | | **RCTs**  For Partial Yes, must have assessed RoB from   unconcealed allocation, *and*   lack of blinding of patients and assessors when assessing outcomes (unnecessary for objective outcomes such as all-cause mortality) | | For Yes, must also have assessed RoB from:   allocation sequence that was not truly random, *and*   selection of the reported result from among multiple measurements or analyses of a specified outcome | |  Yes   Partial Yes  x No   Includes only NRSI | | |
|  | | **NRSI**  For Partial Yes, must have assessed RoB:   from confounding, *and*   from selection bias | | For Yes, must also have assessed RoB:   methods used to ascertain exposures and outcomes, *and*   selection of the reported result from among multiple measurements or analyses of a specified | |  Yes   Partial Yes  x No  Includes only RCTs | | |
| Comments: Not explicitly stated, but an MMAT quality appraisal was undertaken for all studies included | | | | | | | | |

| **10. Did the review authors report outcomes on the sources of funding for the studies included in the review?** | | | | | | |
| --- | --- | --- | --- | --- | --- | --- |
| For Yes:   Must have reported on the sources of funding for individual studies included in the review.  Note: Reporting that the reviewers looked for this information but it was not reported by study authors also qualifies. | | | |  Yes  x No |  |  |
| Comments: None | | | |  |  |  |
| **11. If meta-analysis was performed did the review authors use appropriate methods for statistical combination of results?** | | | | | | |
|  | **RCTs**  For Yes:   The authors justified combining the data in a meta-analysis   AND they used an appropriate weighted technique to combine study results and adjusted for heterogeneity if present.   AND investigated the causes of any heterogeneity | |  Yes   No  x No meta-analysis conducted | | |  |
|  | **For NRSI**  For Yes:   The authors justified combining the data in a meta-analysis   AND they used an appropriate weighted technique to combine study results, adjusting for heterogeneity if present   AND they statistically combined effect estimates from NRSI that were adjusted for confounding, rather than combining raw data, or justified combining raw data when adjusted effect estimates were not available   AND they reported separate summary estimates for RCTs and NRSI separately when both were included in the review | |  Yes   No  x No meta-analysis conducted | | |  |
|  | Comments: | | | | |  |
| **12. If meta-analysis was performed, did the review authors assess the potential impact of RoB in individual studies on the results of the meta-analysis or other evidence synthesis?** | | | | | | |
|  | For Yes:   included only low risk of bias RCTs   OR, if the pooled estimate was based on RCTs and/or NRSI at variable RoB, the authors performed analyses to investigate possible impact of RoB on summary estimates of effect. | |  Yes   No  x No meta-analysis conducted | | |  |
|  | Comments: | | | | |  |
| **13. Did the review authors account for RoB in individual studies when interpreting/ discussing the results of the review?** | | | | | | |
|  | For Yes:   included only low risk of bias RCTs   OR, if RCTs with moderate or high RoB, or NRSI were included the review provided a discussion of the likely impact of RoB on the results | |  Yes  x No | | |  |
|  | Comments: Not explicitly stated, but an MMAT quality appraisal was undertaken for all studies included | | | | |  |
| **14. Did the review authors provide a satisfactory explanation for, and discussion of, any heterogeneity observed in the results of the review?** | | | | | | |
|  | For Yes:   There was no significant heterogeneity in the results  x OR if heterogeneity was present the authors performed an investigation of sources of any heterogeneity in the results and discussed the impact of this on the results of the review | | x Yes   No | | |  |
|  | Comments: Heterogeneity is discussed in relation to outcomes and the paper states due the heterogeneity the study team decided not to conduct a meta-analysis, as this may have resulted in a misrepresentation of the data. | | | | |  |
| **15. If they performed quantitative synthesis did the review authors carry out an adequate investigation of publication bias (small study bias) and discuss its likely impact on the results of the review?** | | | | | | |
|  | For Yes:   performed graphical or statistical tests for publication bias and discussed the likelihood and magnitude of impact of publication bias | |  Yes   No   x No meta-analysis conducted | | |  |
|  | Comments: None | | | | |  |
| **16. Did the review authors report any potential sources of conflict of interest, including any funding they received for conducting the review?** | | | | | | |
|  | For Yes:  x The authors reported no competing interests OR   The authors described their funding sources and how they managed potential conflicts of interest | x Yes   No | | | |  |
|  | Comments: No conflict of interest stated, funding sources not reported on. | | | | |  |

# Study 21 – Nielsen et al

| **1. Did the research questions and inclusion criteria for the review include the components of PICO?** | | | | | | | | |
| --- | --- | --- | --- | --- | --- | --- | --- | --- |
| For Yes:  x Population  x Intervention  x Comparator group  x Outcome | | | Optional (recommended)   Timeframe for follow-up | | x | | Yes No |  |
| Comments: C = Users of patient reported outcomes compared to non-users | | | | | | | |  |
| **2. Did the report of the review contain an explicit statement that the review methods were established prior to the conduct of the review and did the report justify any significant deviations from the protocol?** | | | | | | | | |
|  | For Partial Yes:  The authors state that they had a written protocol or guide that included ALL the following:  x review question(s)  x a search strategy  x inclusion/exclusion criteria  x a risk of bias assessment | | For Yes:  As for partial yes, plus the protocol should be registered and should also have specified:   a meta-analysis/synthesis plan, if appropriate, *and*   a plan for investigating causes of heterogeneity   justification for any deviations from the protocol | |   x  | | Yes  Partial Yes  No |  |
|  | Comments: The scoping review specifies that it used the the framework of Arksey and O’Malley and PRISMAScR  Reporting. | | | | | | |  |
| **3. Did the review authors explain their selection of the study designs for inclusion in the review?** | | | | | | | | |
|  | For Yes, the review should satisfy ONE of the following:   *Explanation for* including only RCTs   OR *Explanation for* including only NRSI   OR *Explanation for* including both RCTs and NRSI | | | | x  | | Yes No |  |
|  | Comments: An explicit explanation is not provided, but the authors included all types of study design | | | | | | |  |
| **4. Did the review authors use a comprehensive literature search strategy?** | | | | | | | | |
|  | For Partial Yes (all the following): | | For Yes, should also have (all the following):   searched the reference lists / bibliographies of included studies   searched trial/study registries   included/consulted content experts in the field   where relevant, searched for grey literature   conducted search within 24 months of completion of the review | |  | |  |  |
|  | x searched at least 2 databases (relevant to research question)  x provided key word and/or search strategy  x justified publication restrictions | |  |  |   x  | | Yes  Partial Yes No |  |
|  | (e.g. language) | |  |  |  | |  |  |
|  | Comments: Language restrictions not indicated. | | | | | | |  |
|  | **5. Did the review authors perform study selection in duplicate?** | | | |  | | |  |
|  | For Yes, either ONE of the following:   at least two reviewers independently agreed on selection of eligible studies and achieved consensus on which studies to include   OR two reviewers selected a sample of eligible studies and achieved good agreement (at least 80 percent), with the remainder selected by one reviewer. | | | | x | | Yes No |  |
|  | Comments: Does not explicitly state protocol for reviewing however does state that the scoping review used the the framework of Arksey and O’Malley and PRISMAScR Reporting. | | | | | | |  |
| **6. Did the review authors perform data extraction in duplicate?** | | | | | | | | |
| For Yes, either ONE of the following:   at least two reviewers achieved consensus on which data to extract from included studies   OR two reviewers extracted data from a sample of eligible studies and achieved good agreement (at least 80 percent), with the remainder extracted by one reviewer. | | | | | |  Yes  x No | | |
| Comments: Does not explicitly state protocol for reviewing however does state that the scoping review used the the framework of Arksey and O’Malley and PRISMAScR Reporting. | | | | | | | | |
| **7. Did the review authors provide a list of excluded studies and justify the exclusions?** | | | | | | | | |
|  | | For Partial Yes:   provided a list of all potentially relevant studies that were read in full-text form but excluded from the review | | For Yes, must also have:   Justified the exclusion from the review of each potentially relevant study | |  Yes   Partial Yes  x No | | |
|  | | Comments: They gave a summary of the reasons for exclusion. | | | | | | |
| **8. Did the review authors describe the included studies in adequate detail?** | | | | | | | | |
|  | | For Partial Yes (ALL the following):  x described populations  x described interventions  x described comparators  x described outcomes  x described research designs | | For Yes, should also have ALL the following:  x described population in detail  x described intervention in detail (including doses where relevant)   described comparator in detail (including doses where relevant)   described study’s setting  x timeframe for follow-up | |  Yes  x Partial Yes   No | | |
|  | | Comments: Considerable information provided for each study. | | | | | | |
| **9. Did the review authors use a satisfactory technique for assessing the risk of bias (RoB) in individual studies that were included in the review?** | | | | | | | | |
|  | | **RCTs**  For Partial Yes, must have assessed RoB from   unconcealed allocation, *and*   lack of blinding of patients and assessors when assessing outcomes (unnecessary for objective outcomes such as all-cause mortality) | | For Yes, must also have assessed RoB from:   allocation sequence that was not truly random, *and*   selection of the reported result from among multiple measurements or analyses of a specified outcome | |  Yes   Partial Yes  x No   Includes only NRSI | | |
|  | | **NRSI**  For Partial Yes, must have assessed RoB:   from confounding, *and*   from selection bias | | For Yes, must also have assessed RoB:   methods used to ascertain exposures and outcomes, *and*   selection of the reported result from among multiple measurements or analyses of a specified | |  Yes   Partial Yes  x No  Includes only RCTs | | |
| Comments: This was a scoping review, and in those authors do not typically assess the quality of included studies. | | | | | | | | |

| **10. Did the review authors report outcomes on the sources of funding for the studies included in the review?** | | | | | | |
| --- | --- | --- | --- | --- | --- | --- |
| For Yes:   Must have reported on the sources of funding for individual studies included in the review.  Note: Reporting that the reviewers looked for this information but it was not reported by study authors also qualifies. | | | |  Yes  x No |  |  |
| Comments: None | | | |  |  |  |
| **11. If meta-analysis was performed did the review authors use appropriate methods for statistical combination of results?** | | | | | | |
|  | **RCTs**  For Yes:   The authors justified combining the data in a meta-analysis   AND they used an appropriate weighted technique to combine study results and adjusted for heterogeneity if present.   AND investigated the causes of any heterogeneity | |  Yes   No  x No meta-analysis conducted | | |  |
|  | **For NRSI**  For Yes:   The authors justified combining the data in a meta-analysis   AND they used an appropriate weighted technique to combine study results, adjusting for heterogeneity if present   AND they statistically combined effect estimates from NRSI that were adjusted for confounding, rather than combining raw data, or justified combining raw data when adjusted effect estimates were not available   AND they reported separate summary estimates for RCTs and NRSI separately when both were included in the review | |  Yes   No  x No meta-analysis conducted | | |  |
|  | Comments: None | | | | |  |
| **12. If meta-analysis was performed, did the review authors assess the potential impact of RoB in individual studies on the results of the meta-analysis or other evidence synthesis?** | | | | | | |
|  | For Yes:   included only low risk of bias RCTs   OR, if the pooled estimate was based on RCTs and/or NRSI at variable RoB, the authors performed analyses to investigate possible impact of RoB on summary estimates of effect. | |  Yes   No   x No meta-analysis conducted | | |  |
|  | Comments: None | | | | |  |
| **13. Did the review authors account for RoB in individual studies when interpreting/ discussing the results of the review?** | | | | | | |
|  | For Yes:   included only low risk of bias RCTs   OR, if RCTs with moderate or high RoB, or NRSI were included the review provided a discussion of the likely impact of RoB on the results | |  Yes  x No | | |  |
|  | Comments: This is a scoping review, and in those the authors do not typically assess the quality of included studies. | | | | |  |
| **14. Did the review authors provide a satisfactory explanation for, and discussion of, any heterogeneity observed in the results of the review?** | | | | | | |
|  | For Yes:   There was no significant heterogeneity in the results   OR if heterogeneity was present the authors performed an investigation of sources of any heterogeneity in the results and discussed the impact of this on the results of the review | | x Yes   No | | |  |
|  | Comments: Heterogeneity not discussed explicitly, but the variety of types of studies is discussed throughout. | | | | |  |
| **15. If they performed quantitative synthesis did the review authors carry out an adequate investigation of publication bias (small study bias) and discuss its likely impact on the results of the review?** | | | | | | |
|  | For Yes:   performed graphical or statistical tests for publication bias and discussed the likelihood and magnitude of impact of publication bias | |  Yes   No   cNo meta-analysis conducted | | |  |
|  | Comments: None | | | | |  |
| **16. Did the review authors report any potential sources of conflict of interest, including any funding they received for conducting the review?** | | | | | | |
|  | For Yes:  x The authors reported no competing interests OR  x The authors described their funding sources and how they managed potential conflicts of interest |  Yes   No | | | |  |
|  | Comments: No conflict of interest declared and no funding received by the authors for the research. | | | | |  |

# Study 22 – OCathail et al

| **1. Did the research questions and inclusion criteria for the review include the components of PICO?** | | | | | | | | |
| --- | --- | --- | --- | --- | --- | --- | --- | --- |
| For Yes:  x Population  x Intervention  n/a Comparator group  x Outcome | | | Optional (recommended)   Timeframe for follow-up | | x | | Yes No |  |
| Comments: The scoping review guided by the methodological framework devised by Arksey and O’Malley, and further amendments that were contributed by Levac et al and the Joanna Briggs Institute on conducting systematic scoping reviews | | | | | | | |  |
| **2. Did the report of the review contain an explicit statement that the review methods were established prior to the conduct of the review and did the report justify any significant deviations from the protocol?** | | | | | | | | |
|  | For Partial Yes:  The authors state that they had a written protocol or guide that included ALL the following:  x review question(s)  x a search strategy  x inclusion/exclusion criteria  n/a a risk of bias assessment | | For Yes:  As for partial yes, plus the protocol should be registered and should also have specified:   a meta-analysis/synthesis plan, if appropriate, *and*   a plan for investigating causes of heterogeneity   justification for any deviations from the protocol | |   x  | | Yes  Partial Yes  No |  |
|  | Comments: A scoping review, and in those authors do not typically asses the quality of included studies. | | | | | | |  |
| **3. Did the review authors explain their selection of the study designs for inclusion in the review?** | | | | | | | | |
|  | For Yes, the review should satisfy ONE of the following:   *Explanation for* including only RCTs   OR *Explanation for* including only NRSI   OR *Explanation for* including both RCTs and NRSI | | | | x  | | Yes No |  |
|  | Comments: An explicit explanation not provided, but the authors included all types of study designs. | | | | | | |  |
| **4. Did the review authors use a comprehensive literature search strategy?** | | | | | | | | |
|  | For Partial Yes (all the following): | | For Yes, should also have (all the following):   searched the reference lists / bibliographies of included studies   searched trial/study registries   included/consulted content experts in the field   where relevant, searched for grey literature   conducted search within 24 months of completion of the review | |  | |  |  |
|  | x searched at least 2 databases (relevant to research question)  x provided key word and/or search strategy   justified publication restrictions | |  |  |   x  | | Yes  Partial Yes No |  |
|  | (e.g. language) | |  |  |  | |  |  |
|  | Comments: Since the inclusion criteria specified UK based studies, a justification for language restrictions is not considered relevant. | | | | | | |  |
|  | **5. Did the review authors perform study selection in duplicate?** | | | |  | | |  |
|  | For Yes, either ONE of the following:  x at least two reviewers independently agreed on selection of eligible studies and achieved consensus on which studies to include   OR two reviewers selected a sample of eligible studies and achieved good agreement (at least 80 percent), with the remainder selected by one reviewer. | | | | x  | | Yes No |  |
|  | Comments: None | | | | | | |  |
| **6. Did the review authors perform data extraction in duplicate?** | | | | | | | | |
| For Yes, either ONE of the following:  x at least two reviewers achieved consensus on which data to extract from included studies   OR two reviewers extracted data from a sample of eligible studies and achieved good agreement (at least 80 percent), with the remainder extracted by one reviewer. | | | | | | x Yes   No | | |
| Comments: None | | | | | | | | |
| **7. Did the review authors provide a list of excluded studies and justify the exclusions?** | | | | | | | | |
|  | | For Partial Yes:   provided a list of all potentially relevant studies that were read in full-text form but excluded from the review | | For Yes, must also have:   Justified the exclusion from the review of each potentially relevant study | |  Yes   Partial Yes  x No | | |
|  | | Comments: Full explanation by study not provided, but a summary of justifications included. | | | | | | |
| **8. Did the review authors describe the included studies in adequate detail?** | | | | | | | | |
|  | | For Partial Yes (ALL the following):  x described populations  x described interventions   described comparators  x described outcomes  x described research designs | | For Yes, should also have ALL the following:   described population in detail   described intervention in detail (including doses where relevant)   described comparator in detail (including doses where relevant)   described study’s setting   timeframe for follow-up | |  Yes  x Partial Yes   No | | |
|  | | Comments: A detailed summary of the studies included. | | | | | | |
| **9. Did the review authors use a satisfactory technique for assessing the risk of bias (RoB) in individual studies that were included in the review?** | | | | | | | | |
|  | | **RCTs**  For Partial Yes, must have assessed RoB from   unconcealed allocation, *and*   lack of blinding of patients and assessors when assessing outcomes (unnecessary for objective outcomes such as all-cause mortality) | | For Yes, must also have assessed RoB from:   allocation sequence that was not truly random, *and*   selection of the reported result from among multiple measurements or analyses of a specified outcome | |  Yes   Partial Yes  x No   Includes only NRSI | | |
|  | | **NRSI**  For Partial Yes, must have assessed RoB:   from confounding, *and*   from selection bias | | For Yes, must also have assessed RoB:   methods used to ascertain exposures and outcomes, *and*   selection of the reported result from among multiple measurements or analyses of a specified | |  Yes   Partial Yes  x No  Includes only RCTs | | |
| Comments: Bias not discussed in the study | | | | | | | | |

| **10. Did the review authors report outcomes on the sources of funding for the studies included in the review?** | | | | | | |
| --- | --- | --- | --- | --- | --- | --- |
| For Yes:   Must have reported on the sources of funding for individual studies included in the review.  Note: Reporting that the reviewers looked for this information but it was not reported by study authors also qualifies. | | | |  Yes  x No |  |  |
| Comments: None | | | |  |  |  |
| **11. If meta-analysis was performed did the review authors use appropriate methods for statistical combination of results?** | | | | | | |
|  | **RCTs**  For Yes:   The authors justified combining the data in a meta-analysis   AND they used an appropriate weighted technique to combine study results and adjusted for heterogeneity if present.   AND investigated the causes of any heterogeneity | |  Yes   No  x No meta-analysis conducted | | |  |
|  | **For NRSI**  For Yes:   The authors justified combining the data in a meta-analysis   AND they used an appropriate weighted technique to combine study results, adjusting for heterogeneity if present   AND they statistically combined effect estimates from NRSI that were adjusted for confounding, rather than combining raw data, or justified combining raw data when adjusted effect estimates were not available   AND they reported separate summary estimates for RCTs and NRSI separately when both were included in the review | |  Yes   No  x No meta-analysis conducted | | |  |
|  | Comments: None | | | | |  |
| **12. If meta-analysis was performed, did the review authors assess the potential impact of RoB in individual studies on the results of the meta-analysis or other evidence synthesis?** | | | | | | |
|  | For Yes:   included only low risk of bias RCTs   OR, if the pooled estimate was based on RCTs and/or NRSI at variable RoB, the authors performed analyses to investigate possible impact of RoB on summary estimates of effect. | |  Yes   No   x No meta-analysis conducted | | |  |
|  | Comments: None | | | | |  |
| **13. Did the review authors account for RoB in individual studies when interpreting/ discussing the results of the review?** | | | | | | |
|  | For Yes:   included only low risk of bias RCTs   OR, if RCTs with moderate or high RoB, or NRSI were included the review provided a discussion of the likely impact of RoB on the results | |  Yes  x No | | |  |
|  | Comments: No mention of how bias was addressed in the study. | | | | |  |
| **14. Did the review authors provide a satisfactory explanation for, and discussion of, any heterogeneity observed in the results of the review?** | | | | | | |
|  | For Yes:   There was no significant heterogeneity in the results  x OR if heterogeneity was present the authors performed an investigation of sources of any heterogeneity in the results and discussed the impact of this on the results of the review | | x Yes   No | | |  |
|  | Comments: Heterogeneity of the study and its implications are discussed. | | | | |  |
| **15. If they performed quantitative synthesis did the review authors carry out an adequate investigation of publication bias (small study bias) and discuss its likely impact on the results of the review?** | | | | | | |
|  | For Yes:   performed graphical or statistical tests for publication bias and discussed the likelihood and magnitude of impact of publication bias | |  Yes   No   x No meta-analysis conducted | | |  |
|  | Comments: | | | | |  |
| **16. Did the review authors report any potential sources of conflict of interest, including any funding they received for conducting the review?** | | | | | | |
|  | For Yes:  x The authors reported no competing interests OR   The authors described their funding sources and how they managed potential conflicts of interest |  Yes   No | | | |  |
|  | Comments: No conflict of interest declared. | | | | |  |

# Study 23 - Ohannessiam et al

| **1. Did the research questions and inclusion criteria for the review include the components of PICO?** | | | | | | | | |
| --- | --- | --- | --- | --- | --- | --- | --- | --- |
| For Yes:  x Population  x Intervention  x Comparator group  x Outcome | | | Optional (recommended)   Timeframe for follow-up | | x | | Yes No |  |
| Comments: | | | | | | | |  |
| **2. Did the report of the review contain an explicit statement that the review methods were established prior to the conduct of the review and did the report justify any significant deviations from the protocol?** | | | | | | | | |
|  | For Partial Yes:  The authors state that they had a written protocol or guide that included ALL the following:  x review question(s)  x a search strategy  x inclusion/exclusion criteria   a risk of bias assessment | | For Yes:  As for partial yes, plus the protocol should be registered and should also have specified:   a meta-analysis/synthesis plan, if appropriate, *and*   a plan for investigating causes of heterogeneity   justification for any deviations from the protocol | |     x | | Yes  Partial Yes  No |  |
|  | Comments: | | | | | | |  |
| **3. Did the review authors explain their selection of the study designs for inclusion in the review?** | | | | | | | | |
|  | For Yes, the review should satisfy ONE of the following:   *Explanation for* including only RCTs   OR *Explanation for* including only NRSI  x OR *Explanation for* including both RCTs and NRSI | | | | x | | Yes No |  |
|  | Comments: | | | | | | |  |
| **4. Did the review authors use a comprehensive literature search strategy?** | | | | | | | | |
|  | For Partial Yes (all the following): | | For Yes, should also have (all the following):   searched the reference lists / bibliographies of included studies   searched trial/study registries   included/consulted content experts in the field   where relevant, searched for grey literature   conducted search within 24 months of completion of the review | |  | |  |  |
|  | x searched at least 2 databases (relevant to research question)  x provided key word and/or search strategy  x justified publication restrictions | |  |  |   x  | | Yes  Partial Yes No |  |
|  | (e.g. language) | |  |  |  | |  |  |
|  | Comments: | | | | | | |  |
|  | **5. Did the review authors perform study selection in duplicate?** | | | |  | | |  |
|  | For Yes, either ONE of the following:  x at least two reviewers independently agreed on selection of eligible studies and achieved consensus on which studies to include   OR two reviewers selected a sample of eligible studies and achieved good agreement (at least 80 percent), with the remainder selected by one reviewer. | | | | x | | Yes No |  |
|  | Comments: | | | | | | |  |
| **6. Did the review authors perform data extraction in duplicate?** | | | | | | | | |
| For Yes, either ONE of the following:  x at least two reviewers achieved consensus on which data to extract from included studies   OR two reviewers extracted data from a sample of eligible studies and achieved good agreement (at least 80 percent), with the remainder extracted by one reviewer. | | | | | | x Yes   No | | |
| Comments: | | | | | | | | |
| **7. Did the review authors provide a list of excluded studies and justify the exclusions?** | | | | | | | | |
|  | | For Partial Yes:   provided a list of all potentially relevant studies that were read in full-text form but excluded from the review | | For Yes, must also have:   Justified the exclusion from the review of each potentially relevant study | |  Yes   Partial Yes  x No | | |
|  | | Comments: | | | | | | |
| **8. Did the review authors describe the included studies in adequate detail?** | | | | | | | | |
|  | | For Partial Yes (ALL the following):  x described populations  x described interventions  x described comparators  x described outcomes  x described research designs | | For Yes, should also have ALL the following:   described population in detail   described intervention in detail (including doses where relevant)   described comparator in detail (including doses where relevant)   described study’s setting   timeframe for follow-up | |  Yes  x Partial Yes   No | | |
|  | | Comments: | | | | | | |
| **9. Did the review authors use a satisfactory technique for assessing the risk of bias (RoB) in individual studies that were included in the review?** | | | | | | | | |
|  | | **RCTs**  For Partial Yes, must have assessed RoB from   unconcealed allocation, *and*   lack of blinding of patients and assessors when assessing outcomes (unnecessary for objective outcomes such as all-cause mortality) | | For Yes, must also have assessed RoB from:   allocation sequence that was not truly random, *and*   selection of the reported result from among multiple measurements or analyses of a specified outcome | |  Yes   Partial Yes  x No   Includes only NRSI | | |
|  | | **NRSI**  For Partial Yes, must have assessed RoB:   from confounding, *and*   from selection bias | | For Yes, must also have assessed RoB:   methods used to ascertain exposures and outcomes, *and*   selection of the reported result from among multiple measurements or analyses of a specified | |  Yes   Partial Yes  x No  Includes only RCTs | | |
| Comments: | | | | | | | | |

| **10. Did the review authors report outcomes on the sources of funding for the studies included in the review?** | | | | | | |
| --- | --- | --- | --- | --- | --- | --- |
| For Yes:   Must have reported on the sources of funding for individual studies included in the review.  Note: Reporting that the reviewers looked for this information but it was not reported by study authors also qualifies. | | | |  Yes  x No |  |  |
| Comments: | | | |  |  |  |
| **11. If meta-analysis was performed did the review authors use appropriate methods for statistical combination of results?** | | | | | | |
|  | **RCTs**  For Yes:   The authors justified combining the data in a meta-analysis   AND they used an appropriate weighted technique to combine study results and adjusted for heterogeneity if present.   AND investigated the causes of any heterogeneity | |  Yes   No  x No meta-analysis conducted | | |  |
|  | **For NRSI**  For Yes:   The authors justified combining the data in a meta-analysis   AND they used an appropriate weighted technique to combine study results, adjusting for heterogeneity if present   AND they statistically combined effect estimates from NRSI that were adjusted for confounding, rather than combining raw data, or justified combining raw data when adjusted effect estimates were not available   AND they reported separate summary estimates for RCTs and NRSI separately when both were included in the review | |  Yes   No  x No meta-analysis conducted | | |  |
|  | Comments: | | | | |  |
| **12. If meta-analysis was performed, did the review authors assess the potential impact of RoB in individual studies on the results of the meta-analysis or other evidence synthesis?** | | | | | | |
|  | For Yes:   included only low risk of bias RCTs   OR, if the pooled estimate was based on RCTs and/or NRSI at variable RoB, the authors performed analyses to investigate possible impact of RoB on summary estimates of effect. | |  Yes   No  x No meta-analysis conducted | | |  |
|  | Comments: | | | | |  |
| **13. Did the review authors account for RoB in individual studies when interpreting/ discussing the results of the review?** | | | | | | |
|  | For Yes:   included only low risk of bias RCTs   OR, if RCTs with moderate or high RoB, or NRSI were included the review provided a discussion of the likely impact of RoB on the results | |  Yes  x No | | |  |
|  | Comments: | | | | |  |
| **14. Did the review authors provide a satisfactory explanation for, and discussion of, any heterogeneity observed in the results of the review?** | | | | | | |
|  | For Yes:   There was no significant heterogeneity in the results   OR if heterogeneity was present the authors performed an investigation of sources of any heterogeneity in the results and discussed the impact of this on the results of the review | |  Yes  x No | | |  |
|  | Comments: | | | | |  |
| **15. If they performed quantitative synthesis did the review authors carry out an adequate investigation of publication bias (small study bias) and discuss its likely impact on the results of the review?** | | | | | | |
|  | For Yes:   performed graphical or statistical tests for publication bias and discussed the likelihood and magnitude of impact of publication bias | |  Yes   No  x No meta-analysis conducted | | |  |
|  | Comments: | | | | |  |
| **16. Did the review authors report any potential sources of conflict of interest, including any funding they received for conducting the review?** | | | | | | |
|  | For Yes:  x The authors reported no competing interests OR   The authors described their funding sources and how they managed potential conflicts of interest | x Yes   No | | | |  |
|  | Comments: | | | | |  |

# Study 24 – Pron et al

| **1. Did the research questions and inclusion criteria for the review include the components of PICO?** | | | | | | | | |
| --- | --- | --- | --- | --- | --- | --- | --- | --- |
| For Yes:  x Population  x Intervention  x Comparator group  x  Outcome | | | Optional (recommended)   Timeframe for follow-up | | x | | Yes No |  |
| Comments: The comparator stated as -To evaluate the safety, effectiveness, and cost-effectiveness of Internet-based device-assisted RMSs for CIEDs compared to usual outpatient in-office monitoring strategies. | | | | | | | |  |
| **2. Did the report of the review contain an explicit statement that the review methods were established prior to the conduct of the review and did the report justify any significant deviations from the protocol?** | | | | | | | | |
|  | For Partial Yes:  The authors state that they had a written protocol or guide that included ALL the following:  x review question(s)  x a search strategy  x inclusion/exclusion criteria  n/a a risk of bias assessment | | For Yes:  As for partial yes, plus the protocol should be registered and should also have specified:   a meta-analysis/synthesis plan, if appropriate, *and*   a plan for investigating causes of heterogeneity   justification for any deviations from the protocol | |   x | | Yes  Partial Yes  No |  |
|  | Comments: Explanation of research protocol provided. This is a systematic review, and in those authors do not typically assess the quality of included studies, however a modified CONSORT Statement Checklist was used to assess Randomized Controlled Trials. | | | | | | |  |
| **3. Did the review authors explain their selection of the study designs for inclusion in the review?** | | | | | | | | |
|  | For Yes, the review should satisfy ONE of the following:   *Explanation for* including only RCTs   OR *Explanation for* including only NRSI   OR *Explanation for* including both RCTs and NRSI | | | | x  | | Yes No |  |
|  | Comments: An explicit explanation is not provided, however selected designs of randomized controlled trials (RCTs), systematic reviews and meta-analyses, cohort and controlled clinical studies; is stated in the inclusion criteria and is considered relevant to the objectives of the SR . | | | | | | |  |
| **4. Did the review authors use a comprehensive literature search strategy?** | | | | | | | | |
|  | For Partial Yes (all the following): | | For Yes, should also have (all the following):   searched the reference lists / bibliographies of included studies   searched trial/study registries   included/consulted content experts in the field   where relevant, searched for grey literature   conducted search within 24 months of completion of the review | |  | |  |  |
|  | x searched at least 2 databases (relevant to research question)  x provided key word and/or search strategy  x justified publication restrictions | |  |  |   x  | | Yes  Partial Yes No |  |
|  | (e.g. language) | |  |  |  | |  |  |
|  | Comments: Justification for language restrictions implied in the inclusion/exclusion criteria | | | | | | |  |
|  | **5. Did the review authors perform study selection in duplicate?** | | | |  | | |  |
|  | For Yes, either ONE of the following:   at least two reviewers independently agreed on selection of eligible studies and achieved consensus on which studies to include   OR two reviewers selected a sample of eligible studies and achieved good agreement (at least 80 percent), with the remainder selected by one reviewer. | | | | x | | Yes No |  |
|  | Comments: Review process states that *Abstracts were reviewed by a single reviewer and, for those studies meeting the eligibility criteria, full-text articles were obtained.* | | | | | | |  |
| **6. Did the review authors perform data extraction in duplicate?** | | | | | | | | |
| For Yes, either ONE of the following:   at least two reviewers achieved consensus on which data to extract from included studies   OR two reviewers extracted data from a sample of eligible studies and achieved good agreement (at least 80 percent), with the remainder extracted by one reviewer. | | | | | |  Yes  x No | | |
| Comments: Review process not elaborated on in the paper. | | | | | | | | |
| **7. Did the review authors provide a list of excluded studies and justify the exclusions?** | | | | | | | | |
|  | | For Partial Yes:   provided a list of all potentially relevant studies that were read in full-text form but excluded from the review | | For Yes, must also have:   Justified the exclusion from the review of each potentially relevant study | |  Yes   Partial Yes  x No | | |
|  | | Comments: None | | | | | | |
| **8. Did the review authors describe the included studies in adequate detail?** | | | | | | | | |
|  | | For Partial Yes (ALL the following):  x described populations  x described interventions  x described comparators  x described outcomes  x described research designs | | For Yes, should also have ALL the following:   described population in detail  x described intervention in detail (including doses where relevant)   described comparator in detail (including doses where relevant)   described study’s setting  x timeframe for follow-up | |  Yes  x Partial Yes   No | | |
|  | | Comments: Considerable information extracted into the summary tables. | | | | | | |
| **9. Did the review authors use a satisfactory technique for assessing the risk of bias (RoB) in individual studies that were included in the review?** | | | | | | | | |
|  | | **RCTs**  For Partial Yes, must have assessed RoB from  x unconcealed allocation, *and*  x lack of blinding of patients and assessors when assessing outcomes (unnecessary for objective outcomes such as all-cause mortality) | | For Yes, must also have assessed RoB from:  x allocation sequence that was not truly random, *and*   selection of the reported result from among multiple measurements or analyses of a specified outcome | |  Yes  x Partial Yes   No   Includes only NRSI | | |
|  | | **NRSI**  For Partial Yes, must have assessed RoB:   from confounding, *and*   from selection bias | | For Yes, must also have assessed RoB:   methods used to ascertain exposures and outcomes, *and*   selection of the reported result from among multiple measurements or analyses of a specified | |  Yes  x Partial Yes   No  Includes only RCTs | | |
| Comments: Used the CONSORT Statement Checklist for Randomized Controlled Trials. Other studies not assessed for quality. | | | | | | | | |

| **10. Did the review authors report outcomes on the sources of funding for the studies included in the review?** | | | | | | |
| --- | --- | --- | --- | --- | --- | --- |
| For Yes:   Must have reported on the sources of funding for individual studies included in the review.  Note: Reporting that the reviewers looked for this information but it was not reported by study authors also qualifies. | | | |  Yes  x No |  |  |
| Comments: Funding information for one study provided, but this is not provided for the rest. | | | |  |  |  |
| **11. If meta-analysis was performed did the review authors use appropriate methods for statistical combination of results?** | | | | | | |
|  | **RCTs**  For Yes:   The authors justified combining the data in a meta-analysis   AND they used an appropriate weighted technique to combine study results and adjusted for heterogeneity if present.   AND investigated the causes of any heterogeneity | |  Yes   No  x No meta-analysis conducted | | |  |
|  | **For NRSI**  For Yes:   The authors justified combining the data in a meta-analysis   AND they used an appropriate weighted technique to combine study results, adjusting for heterogeneity if present   AND they statistically combined effect estimates from NRSI that were adjusted for confounding, rather than combining raw data, or justified combining raw data when adjusted effect estimates were not available   AND they reported separate summary estimates for RCTs and NRSI separately when both were included in the review | |  Yes   No  x No meta-analysis conducted | | |  |
|  | Comments: None | | | | |  |
| **12. If meta-analysis was performed, did the review authors assess the potential impact of RoB in individual studies on the results of the meta-analysis or other evidence synthesis?** | | | | | | |
|  | For Yes:   included only low risk of bias RCTs   OR, if the pooled estimate was based on RCTs and/or NRSI at variable RoB, the authors performed analyses to investigate possible impact of RoB on summary estimates of effect. | |  Yes   No   x No meta-analysis conducted | | |  |
|  | Comments: None | | | | |  |
| **13. Did the review authors account for RoB in individual studies when interpreting/ discussing the results of the review?** | | | | | | |
|  | For Yes:   included only low risk of bias RCTs   OR, if RCTs with moderate or high RoB, or NRSI were included the review provided a discussion of the likely impact of RoB on the results | |  Yes  x No | | |  |
|  | Comments: All RCT’s were assessed for quality, but this was not considered in the results. | | | | |  |
| **14. Did the review authors provide a satisfactory explanation for, and discussion of, any heterogeneity observed in the results of the review?** | | | | | | |
|  | For Yes:   There was no significant heterogeneity in the results   OR if heterogeneity was present the authors performed an investigation of sources of any heterogeneity in the results and discussed the impact of this on the results of the review | | x Yes   No | | |  |
|  | Comments: Heterogeneity is not discussed explicitly in the results, but variability of studies discussed in the text. | | | | |  |
| **15. If they performed quantitative synthesis did the review authors carry out an adequate investigation of publication bias (small study bias) and discuss its likely impact on the results of the review?** | | | | | | |
|  | For Yes:   performed graphical or statistical tests for publication bias and discussed the likelihood and magnitude of impact of publication bias | |  Yes   No   x No meta-analysis conducted | | |  |
|  | Comments: None | | | | |  |
| **16. Did the review authors report any potential sources of conflict of interest, including any funding they received for conducting the review?** | | | | | | |
|  | For Yes:  x The authors reported no competing interests OR  x The authors described their funding sources and how they managed potential conflicts of interest | x Yes   No | | | |  |
|  | Comments: None | | | | |  |

# Study 25 - Raja et al

| **1. Did the research questions and inclusion criteria for the review include the components of PICO?** | | | | | | | | |
| --- | --- | --- | --- | --- | --- | --- | --- | --- |
| For Yes:  x Population  x Intervention   Comparator group  x Outcome | | | Optional (recommended)   Timeframe for follow-up | | x | | Yes No |  |
| Comments: | | | | | | | |  |
| **2. Did the report of the review contain an explicit statement that the review methods were established prior to the conduct of the review and did the report justify any significant deviations from the protocol?** | | | | | | | | |
|  | For Partial Yes:  The authors state that they had a written protocol or guide that included ALL the following:  x review question(s)  x a search strategy  x inclusion/exclusion criteria   a risk of bias assessment | | For Yes:  As for partial yes, plus the protocol should be registered and should also have specified:   a meta-analysis/synthesis plan, if appropriate, *and*   a plan for investigating causes of heterogeneity   justification for any deviations from the protocol | |     x | | Yes  Partial Yes  No |  |
|  | Comments: | | | | | | |  |
| **3. Did the review authors explain their selection of the study designs for inclusion in the review?** | | | | | | | | |
|  | For Yes, the review should satisfy ONE of the following:   *Explanation for* including only RCTs   OR *Explanation for* including only NRSI  x OR *Explanation for* including both RCTs and NRSI | | | | x | | Yes No |  |
|  | Comments: | | | | | | |  |
| **4. Did the review authors use a comprehensive literature search strategy?** | | | | | | | | |
|  | For Partial Yes (all the following): | | For Yes, should also have (all the following):  x searched the reference lists / bibliographies of included studies   searched trial/study registries   included/consulted content experts in the field  x where relevant, searched for grey literature  x conducted search within 24 months of completion of the review | |  | |  |  |
|  | x searched at least 2 databases (relevant to research question)  x provided key word and/or search strategy  x justified publication restrictions | |  |  |   x  | | Yes  Partial Yes No |  |
|  | (e.g. language) | |  |  |  | |  |  |
|  | Comments: | | | | | | |  |
|  | **5. Did the review authors perform study selection in duplicate?** | | | |  | | |  |
|  | For Yes, either ONE of the following:  x at least two reviewers independently agreed on selection of eligible studies and achieved consensus on which studies to include   OR two reviewers selected a sample of eligible studies and achieved good agreement (at least 80 percent), with the remainder selected by one reviewer. | | | | x | | Yes No |  |
|  | Comments: | | | | | | |  |
| **6. Did the review authors perform data extraction in duplicate?** | | | | | | | | |
| For Yes, either ONE of the following:   at least two reviewers achieved consensus on which data to extract from included studies  x OR two reviewers extracted data from a sample of eligible studies and achieved good agreement (at least 80 percent), with the remainder extracted by one reviewer. | | | | | | x Yes   No | | |
| Comments: | | | | | | | | |
| **7. Did the review authors provide a list of excluded studies and justify the exclusions?** | | | | | | | | |
|  | | For Partial Yes:   provided a list of all potentially relevant studies that were read in full-text form but excluded from the review | | For Yes, must also have:   Justified the exclusion from the review of each potentially relevant study | |  Yes   Partial Yes  x No | | |
|  | | Comments: | | | | | | |
| **8. Did the review authors describe the included studies in adequate detail?** | | | | | | | | |
|  | | For Partial Yes (ALL the following):  x described populations  x described interventions   described comparators  x described outcomes  x described research designs | | For Yes, should also have ALL the following:   described population in detail   described intervention in detail (including doses where relevant)   described comparator in detail (including doses where relevant)   described study’s setting   timeframe for follow-up | |  Yes  x Partial Yes   No | | |
|  | | Comments: | | | | | | |
| **9. Did the review authors use a satisfactory technique for assessing the risk of bias (RoB) in individual studies that were included in the review?** | | | | | | | | |
|  | | **RCTs**  For Partial Yes, must have assessed RoB from   unconcealed allocation, *and*   lack of blinding of patients and assessors when assessing outcomes (unnecessary for objective outcomes such as all-cause mortality) | | For Yes, must also have assessed RoB from:   allocation sequence that was not truly random, *and*   selection of the reported result from among multiple measurements or analyses of a specified outcome | |  Yes   Partial Yes  x No   Includes only NRSI | | |
|  | | **NRSI**  For Partial Yes, must have assessed RoB:   from confounding, *and*   from selection bias | | For Yes, must also have assessed RoB:   methods used to ascertain exposures and outcomes, *and*   selection of the reported result from among multiple measurements or analyses of a specified | |  Yes   Partial Yes  x No  Includes only RCTs | | |
| Comments: | | | | | | | | |

| **10. Did the review authors report outcomes on the sources of funding for the studies included in the review?** | | | | | | |
| --- | --- | --- | --- | --- | --- | --- |
| For Yes:   Must have reported on the sources of funding for individual studies included in the review.  Note: Reporting that the reviewers looked for this information but it was not reported by study authors also qualifies. | | | |  Yes  x No |  |  |
| Comments: | | | |  |  |  |
| **11. If meta-analysis was performed did the review authors use appropriate methods for statistical combination of results?** | | | | | | |
|  | **RCTs**  For Yes:   The authors justified combining the data in a meta-analysis   AND they used an appropriate weighted technique to combine study results and adjusted for heterogeneity if present.   AND investigated the causes of any heterogeneity | |  Yes   No  x No meta-analysis conducted | | |  |
|  | **For NRSI**  For Yes:   The authors justified combining the data in a meta-analysis   AND they used an appropriate weighted technique to combine study results, adjusting for heterogeneity if present   AND they statistically combined effect estimates from NRSI that were adjusted for confounding, rather than combining raw data, or justified combining raw data when adjusted effect estimates were not available   AND they reported separate summary estimates for RCTs and NRSI separately when both were included in the review | |  Yes   No  x No meta-analysis conducted | | |  |
|  | Comments: | | | | |  |
| **12. If meta-analysis was performed, did the review authors assess the potential impact of RoB in individual studies on the results of the meta-analysis or other evidence synthesis?** | | | | | | |
|  | For Yes:   included only low risk of bias RCTs   OR, if the pooled estimate was based on RCTs and/or NRSI at variable RoB, the authors performed analyses to investigate possible impact of RoB on summary estimates of effect. | |  Yes   No  x No meta-analysis conducted | | |  |
|  | Comments: | | | | |  |
| **13. Did the review authors account for RoB in individual studies when interpreting/ discussing the results of the review?** | | | | | | |
|  | For Yes:   included only low risk of bias RCTs   OR, if RCTs with moderate or high RoB, or NRSI were included the review provided a discussion of the likely impact of RoB on the results | |  Yes  x No | | |  |
|  | Comments: | | | | |  |
| **14. Did the review authors provide a satisfactory explanation for, and discussion of, any heterogeneity observed in the results of the review?** | | | | | | |
|  | For Yes:   There was no significant heterogeneity in the results   OR if heterogeneity was present the authors performed an investigation of sources of any heterogeneity in the results and discussed the impact of this on the results of the review | |  Yes  x No | | |  |
|  | Comments: | | | | |  |
| **15. If they performed quantitative synthesis did the review authors carry out an adequate investigation of publication bias (small study bias) and discuss its likely impact on the results of the review?** | | | | | | |
|  | For Yes:   performed graphical or statistical tests for publication bias and discussed the likelihood and magnitude of impact of publication bias | |  Yes   No  x No meta-analysis conducted | | |  |
|  | Comments: | | | | |  |
| **16. Did the review authors report any potential sources of conflict of interest, including any funding they received for conducting the review?** | | | | | | |
|  | For Yes:   The authors reported no competing interests OR  x The authors described their funding sources and how they managed potential conflicts of interest | x Yes   No | | | |  |
|  | Comments: | | | | |  |

# Study 26 – Simmonds-Buckley 2020

| **1. Did the research questions and inclusion criteria for the review include the components of PICO?** | | | | | | | | |
| --- | --- | --- | --- | --- | --- | --- | --- | --- |
| For Yes:  x Population  x Intervention  x Comparator group  x Outcome | | | Optional (recommended)  x Timeframe for follow-up | | x | | Yes No |  |
| Comments: | | | | | | | |  |
| **2. Did the report of the review contain an explicit statement that the review methods were established prior to the conduct of the review and did the report justify any significant deviations from the protocol?** | | | | | | | | |
|  | For Partial Yes:  The authors state that they had a written protocol or guide that included ALL the following:  x review question(s)  x a search strategy  x inclusion/exclusion criteria  x a risk of bias assessment | | For Yes:  As for partial yes, plus the protocol should be registered and should also have specified:  x a meta-analysis/synthesis plan, if appropriate, *and*  x a plan for investigating causes of heterogeneity   justification for any deviations from the protocol | |   X   | | Yes  Partial Yes  No |  |
|  | Comments: | | | | | | |  |
| **3. Did the review authors explain their selection of the study designs for inclusion in the review?** | | | | | | | | |
|  | For Yes, the review should satisfy ONE of the following:  x *Explanation for* including only RCTs   OR *Explanation for* including only NRSI   OR *Explanation for* including both RCTs and NRSI | | | | x | | Yes No |  |
|  | Comments: | | | | | | |  |
| **4. Did the review authors use a comprehensive literature search strategy?** | | | | | | | | |
|  | For Partial Yes (all the following): | | For Yes, should also have (all the following):  x searched the reference lists / bibliographies of included studies  x searched trial/study registries  x included/consulted content experts in the field  x where relevant, searched for grey literature  x conducted search within 24 months of completion of the review | |  | |  |  |
|  | x searched at least 2 databases (relevant to research question)  x provided key word and/or search strategy  x justified publication restrictions | |  |  | x   | | Yes  Partial Yes No |  |
|  | (e.g. language) | |  |  |  | |  |  |
|  | Comments: | | | | | | |  |
|  | **5. Did the review authors perform study selection in duplicate?** | | | |  | | |  |
|  | For Yes, either ONE of the following:  x at least two reviewers independently agreed on selection of eligible studies and achieved consensus on which studies to include   OR two reviewers selected a sample of eligible studies and achieved good agreement (at least 80 percent), with the remainder selected by one reviewer. | | | | x | | Yes No |  |
|  | Comments: | | | | | | |  |
| **6. Did the review authors perform data extraction in duplicate?** | | | | | | | | |
| For Yes, either ONE of the following:  x at least two reviewers achieved consensus on which data to extract from included studies   OR two reviewers extracted data from a sample of eligible studies and achieved good agreement (at least 80 percent), with the remainder extracted by one reviewer. | | | | | | x Yes   No | | |
| Comments: | | | | | | | | |
| **7. Did the review authors provide a list of excluded studies and justify the exclusions?** | | | | | | | | |
|  | | For Partial Yes:   provided a list of all potentially relevant studies that were read in full-text form but excluded from the review | | For Yes, must also have:   Justified the exclusion from the review of each potentially relevant study | |  Yes   Partial Yes  x No | | |
|  | | Comments: | | | | | | |
| **8. Did the review authors describe the included studies in adequate detail?** | | | | | | | | |
|  | | For Partial Yes (ALL the following):  x described populations  x described interventions  x described comparators  x described outcomes  x described research designs | | For Yes, should also have ALL the following:  x described population in detail  x described intervention in detail (including doses where relevant)  x described comparator in detail (including doses where relevant)  x described study’s setting  x timeframe for follow-up | | x Yes   Partial Yes   No | | |
|  | | Comments: | | | | | | |
| **9. Did the review authors use a satisfactory technique for assessing the risk of bias (RoB) in individual studies that were included in the review?** | | | | | | | | |
|  | | **RCTs**  For Partial Yes, must have assessed RoB from  x unconcealed allocation, *and*  x lack of blinding of patients and assessors when assessing outcomes (unnecessary for objective outcomes such as all-cause mortality) | | For Yes, must also have assessed RoB from:   allocation sequence that was not truly random, *and*  x selection of the reported result from among multiple measurements or analyses of a specified outcome | |  Yes  x Partial Yes   No   Includes only NRSI | | |
|  | | **NRSI**  For Partial Yes, must have assessed RoB:   from confounding, *and*   from selection bias | | For Yes, must also have assessed RoB:   methods used to ascertain exposures and outcomes, *and*   selection of the reported result from among multiple measurements or analyses of a specified | |  Yes   Partial Yes   No x Includes only RCTs | | |
| Comments: | | | | | | | | |

| **10. Did the review authors report outcomes on the sources of funding for the studies included in the review?** | | | | | | |
| --- | --- | --- | --- | --- | --- | --- |
| For Yes:   Must have reported on the sources of funding for individual studies included in the review.  Note: Reporting that the reviewers looked for this information but it was not reported by study authors also qualifies. | | | |  Yes   No |  |  |
| Comments: | | | |  |  |  |
| **11. If meta-analysis was performed did the review authors use appropriate methods for statistical combination of results?** | | | | | | |
|  | **RCTs**  For Yes:  x The authors justified combining the data in a meta-analysis  x AND they used an appropriate weighted technique to combine study results and adjusted for heterogeneity if present.  x AND investigated the causes of any heterogeneity | | x Yes   No   No meta-analysis conducted | | |  |
|  | **For NRSI**  For Yes:   The authors justified combining the data in a meta-analysis   AND they used an appropriate weighted technique to combine study results, adjusting for heterogeneity if present   AND they statistically combined effect estimates from NRSI that were adjusted for confounding, rather than combining raw data, or justified combining raw data when adjusted effect estimates were not available   AND they reported separate summary estimates for RCTs and NRSI separately when both were included in the review | |  Yes   No   No meta-analysis conducted | | |  |
|  | Comments: | | | | |  |
| **12. If meta-analysis was performed, did the review authors assess the potential impact of RoB in individual studies on the results of the meta-analysis or other evidence synthesis?** | | | | | | |
|  | For Yes:   included only low risk of bias RCTs  x OR, if the pooled estimate was based on RCTs and/or NRSI at variable RoB, the authors performed analyses to investigate possible impact of RoB on summary estimates of effect. | | x Yes   No   No meta-analysis conducted | | |  |
|  | Comments: | | | | |  |
| **13. Did the review authors account for RoB in individual studies when interpreting/ discussing the results of the review?** | | | | | | |
|  | For Yes:   included only low risk of bias RCTs  x OR, if RCTs with moderate or high RoB, or NRSI were included the review provided a discussion of the likely impact of RoB on the results | | x Yes   No | | |  |
|  | Comments: | | | | |  |
| **14. Did the review authors provide a satisfactory explanation for, and discussion of, any heterogeneity observed in the results of the review?** | | | | | | |
|  | For Yes:   There was no significant heterogeneity in the results  x OR if heterogeneity was present the authors performed an investigation of sources of any heterogeneity in the results and discussed the impact of this on the results of the review | | x Yes   No | | |  |
|  | Comments: | | | | |  |
| **15. If they performed quantitative synthesis did the review authors carry out an adequate investigation of publication bias (small study bias) and discuss its likely impact on the results of the review?** | | | | | | |
|  | For Yes:  x performed graphical or statistical tests for publication bias and discussed the likelihood and magnitude of impact of publication bias | | x Yes   No   No meta-analysis conducted | | |  |
|  | Comments: | | | | |  |
| **16. Did the review authors report any potential sources of conflict of interest, including any funding they received for conducting the review?** | | | | | | |
|  | For Yes:   The authors reported no competing interests OR  x The authors described their funding sources and how they managed potential conflicts of interest | x Yes   No | | | |  |
|  | Comments: | | | | |  |

# Study 27 – Singh 2016

| **1. Did the research questions and inclusion criteria for the review include the components of PICO?** | | | | | | | | |
| --- | --- | --- | --- | --- | --- | --- | --- | --- |
| For Yes:  x Population  x Intervention  x Comparator group  x Outcome | | | Optional (recommended)   Timeframe for follow-up | | x | | Yes No |  |
| Comments: | | | | | | | |  |
| **2. Did the report of the review contain an explicit statement that the review methods were established prior to the conduct of the review and did the report justify any significant deviations from the protocol?** | | | | | | | | |
|  | For Partial Yes:  The authors state that they had a written protocol or guide that included ALL the following:  x review question(s)  x a search strategy  x inclusion/exclusion criteria   a risk of bias assessment | | For Yes:  As for partial yes, plus the protocol should be registered and should also have specified:   a meta-analysis/synthesis plan, if appropriate, *and*   a plan for investigating causes of heterogeneity   justification for any deviations from the protocol | |     x | | Yes  Partial Yes  No |  |
|  | Comments: | | | | | | |  |
| **3. Did the review authors explain their selection of the study designs for inclusion in the review?** | | | | | | | | |
|  | For Yes, the review should satisfy ONE of the following:   *Explanation for* including only RCTs   OR *Explanation for* including only NRSI  x OR *Explanation for* including both RCTs and NRSI | | | | x | | Yes No |  |
|  | Comments: | | | | | | |  |
| **4. Did the review authors use a comprehensive literature search strategy?** | | | | | | | | |
|  | For Partial Yes (all the following): | | For Yes, should also have (all the following):   searched the reference lists / bibliographies of included studies   searched trial/study registries   included/consulted content experts in the field   where relevant, searched for grey literature   conducted search within 24 months of completion of the review | |  | |  |  |
|  | x searched at least 2 databases (relevant to research question)  x provided key word and/or search strategy  x justified publication restrictions | |  |  |   x  | | Yes  Partial Yes No |  |
|  | (e.g. language) | |  |  |  | |  |  |
|  | Comments: | | | | | | |  |
|  | **5. Did the review authors perform study selection in duplicate?** | | | |  | | |  |
|  | For Yes, either ONE of the following:   at least two reviewers independently agreed on selection of eligible studies and achieved consensus on which studies to include   OR two reviewers selected a sample of eligible studies and achieved good agreement (at least 80 percent), with the remainder selected by one reviewer. | | | |   x | | Yes No  N/A |  |
|  | Comments: | | | | | | |  |
| **6. Did the review authors perform data extraction in duplicate?** | | | | | | | | |
| For Yes, either ONE of the following:   at least two reviewers achieved consensus on which data to extract from included studies   OR two reviewers extracted data from a sample of eligible studies and achieved good agreement (at least 80 percent), with the remainder extracted by one reviewer. | | | | | |  Yes   No  x N/A | | |
| Comments: | | | | | | | | |
| **7. Did the review authors provide a list of excluded studies and justify the exclusions?** | | | | | | | | |
|  | | For Partial Yes:   provided a list of all potentially relevant studies that were read in full-text form but excluded from the review | | For Yes, must also have:   Justified the exclusion from the review of each potentially relevant study | |  Yes   Partial Yes  x No | | |
|  | | Comments: | | | | | | |
| **8. Did the review authors describe the included studies in adequate detail?** | | | | | | | | |
|  | | For Partial Yes (ALL the following):  x described populations  x described interventions  x described comparators  x described outcomes  x described research designs | | For Yes, should also have ALL the following:  x described population in detail  x described intervention in detail (including doses where relevant)  x described comparator in detail (including doses where relevant)  x described study’s setting  x timeframe for follow-up | | x Yes   Partial Yes   No | | |
|  | | Comments: | | | | | | |
| **9. Did the review authors use a satisfactory technique for assessing the risk of bias (RoB) in individual studies that were included in the review?** | | | | | | | | |
|  | | **RCTs**  For Partial Yes, must have assessed RoB from   unconcealed allocation, *and*   lack of blinding of patients and assessors when assessing outcomes (unnecessary for objective outcomes such as all-cause mortality) | | For Yes, must also have assessed RoB from:   allocation sequence that was not truly random, *and*   selection of the reported result from among multiple measurements or analyses of a specified outcome | |  Yes   Partial Yes  x No   Includes only NRSI | | |
|  | | **NRSI**  For Partial Yes, must have assessed RoB:   from confounding, *and*   from selection bias | | For Yes, must also have assessed RoB:   methods used to ascertain exposures and outcomes, *and*   selection of the reported result from among multiple measurements or analyses of a specified | |  Yes   Partial Yes  x No  Includes only RCTs | | |
| Comments: | | | | | | | | |

| **10. Did the review authors report outcomes on the sources of funding for the studies included in the review?** | | | | | | |
| --- | --- | --- | --- | --- | --- | --- |
| For Yes:   Must have reported on the sources of funding for individual studies included in the review.  Note: Reporting that the reviewers looked for this information but it was not reported by study authors also qualifies. | | | |  Yes  x No |  |  |
| Comments: | | | |  |  |  |
| **11. If meta-analysis was performed did the review authors use appropriate methods for statistical combination of results?** | | | | | | |
|  | **RCTs**  For Yes:   The authors justified combining the data in a meta-analysis   AND they used an appropriate weighted technique to combine study results and adjusted for heterogeneity if present.   AND investigated the causes of any heterogeneity | |  Yes   No  x No meta-analysis conducted | | |  |
|  | **For NRSI**  For Yes:   The authors justified combining the data in a meta-analysis   AND they used an appropriate weighted technique to combine study results, adjusting for heterogeneity if present   AND they statistically combined effect estimates from NRSI that were adjusted for confounding, rather than combining raw data, or justified combining raw data when adjusted effect estimates were not available   AND they reported separate summary estimates for RCTs and NRSI separately when both were included in the review | |  Yes   No  x No meta-analysis conducted | | |  |
|  | Comments: | | | | |  |
| **12. If meta-analysis was performed, did the review authors assess the potential impact of RoB in individual studies on the results of the meta-analysis or other evidence synthesis?** | | | | | | |
|  | For Yes:   included only low risk of bias RCTs   OR, if the pooled estimate was based on RCTs and/or NRSI at variable RoB, the authors performed analyses to investigate possible impact of RoB on summary estimates of effect. | |  Yes   No  x No meta-analysis conducted | | |  |
|  | Comments: | | | | |  |
| **13. Did the review authors account for RoB in individual studies when interpreting/ discussing the results of the review?** | | | | | | |
|  | For Yes:   included only low risk of bias RCTs   OR, if RCTs with moderate or high RoB, or NRSI were included the review provided a discussion of the likely impact of RoB on the results | |  Yes  x No | | |  |
|  | Comments: | | | | |  |
| **14. Did the review authors provide a satisfactory explanation for, and discussion of, any heterogeneity observed in the results of the review?** | | | | | | |
|  | For Yes:   There was no significant heterogeneity in the results   OR if heterogeneity was present the authors performed an investigation of sources of any heterogeneity in the results and discussed the impact of this on the results of the review | |  Yes  x No | | |  |
|  | Comments: | | | | |  |
| **15. If they performed quantitative synthesis did the review authors carry out an adequate investigation of publication bias (small study bias) and discuss its likely impact on the results of the review?** | | | | | | |
|  | For Yes:   performed graphical or statistical tests for publication bias and discussed the likelihood and magnitude of impact of publication bias | |  Yes   No  x No meta-analysis conducted | | |  |
|  | Comments: | | | | |  |
| **16. Did the review authors report any potential sources of conflict of interest, including any funding they received for conducting the review?** | | | | | | |
|  | For Yes:   The authors reported no competing interests OR  x The authors described their funding sources and how they managed potential conflicts of interest | x Yes   No | | | |  |
|  | Comments: | | | | |  |

# Study 28 - Tokgoz 2022

| **1. Did the research questions and inclusion criteria for the review include the components of PICO?** | | | | | | | | |
| --- | --- | --- | --- | --- | --- | --- | --- | --- |
| For Yes:  x Population  x Intervention  x  Comparator group  x  Outcome | | | Optional (recommended)   Timeframe for follow-up | | x | | YesNo |  |
| Comments: | | | | | | | |  |
| **2. Did the report of the review contain an explicit statement that the review methods were established prior to the conduct of the review and did the report justify any significant deviations from the protocol?** | | | | | | | | |
|  | For Partial Yes:  The authors state that they had a written protocol or guide that included ALL the following:  x review question(s)  x a search strategy  x inclusion/exclusion criteria  x a risk of bias assessment | | For Yes:  As for partial yes, plus the protocol should be registered and should also have specified:  x a meta-analysis/synthesis plan, if appropriate, *and*  x a plan for investigating causes of heterogeneity  x justification for any deviations from the protocol | | x   | | Yes  Partial Yes  No |  |
|  | Comments: | | | | | | |  |
| **3. Did the review authors explain their selection of the study designs for inclusion in the review?** | | | | | | | | |
|  | For Yes, the review should satisfy ONE of the following:  x *Explanation for* including only RCTs   OR *Explanation for* including only NRSI   OR *Explanation for* including both RCTs and NRSI | | | | x | | Yes No |  |
|  | Comments: | | | | | | |  |
| **4. Did the review authors use a comprehensive literature search strategy?** | | | | | | | | |
|  | For Partial Yes (all the following): | | For Yes, should also have (all the following):   searched the reference lists / bibliographies of included studies   searched trial/study registries   included/consulted content experts in the field   where relevant, searched for grey literature   conducted search within 24 months of completion of the review | |  | |  |  |
|  | x searched at least 2 databases (relevant to research question)  x provided key word and/or search strategy  x justified publication restrictions | |  |  |   x  | | Yes  Partial Yes No |  |
|  | (e.g. language) | |  |  |  | |  |  |
|  | Comments: | | | | | | |  |
|  | **5. Did the review authors perform study selection in duplicate?** | | | |  | | |  |
|  | For Yes, either ONE of the following:  x at least two reviewers independently agreed on selection of eligible studies and achieved consensus on which studies to include   OR two reviewers selected a sample of eligible studies and achieved good agreement (at least 80 percent), with the remainder selected by one reviewer. | | | | x | | Yes No |  |
|  | Comments: | | | | | | |  |
| **6. Did the review authors perform data extraction in duplicate?** | | | | | | | | |
| For Yes, either ONE of the following:  x at least two reviewers achieved consensus on which data to extract from included studies   OR two reviewers extracted data from a sample of eligible studies and achieved good agreement (at least 80 percent), with the remainder extracted by one reviewer. | | | | | | x Yes   No | | |
| Comments: | | | | | | | | |
| **7. Did the review authors provide a list of excluded studies and justify the exclusions?** | | | | | | | | |
|  | | For Partial Yes:   provided a list of all potentially relevant studies that were read in full-text form but excluded from the review | | For Yes, must also have:   Justified the exclusion from the review of each potentially relevant study | |  Yes   Partial Yes  x No | | |
|  | | Comments: | | | | | | |
| **8. Did the review authors describe the included studies in adequate detail?** | | | | | | | | |
|  | | For Partial Yes (ALL the following):  x described populations  x described interventions  xdescribed comparators  x described outcomes  x described research designs | | For Yes, should also have ALL the following:   described population in detail   described intervention in detail (including doses where relevant)   described comparator in detail (including doses where relevant)   described study’s setting   timeframe for follow-up | |  Yes  x Partial Yes   No | | |
|  | | Comments: | | | | | | |
| **9. Did the review authors use a satisfactory technique for assessing the risk of bias (RoB) in individual studies that were included in the review?** | | | | | | | | |
|  | | **RCTs**  For Partial Yes, must have assessed RoB from  x unconcealed allocation, *and*  x lack of blinding of patients and assessors when assessing outcomes (unnecessary for objective outcomes such as all-cause mortality) | | For Yes, must also have assessed RoB from:  x allocation sequence that was not truly random, *and*  x selection of the reported result from among multiple measurements or analyses of a specified outcome | | x Yes   Partial Yes   No   Includes only NRSI | | |
|  | | **NRSI**  For Partial Yes, must have assessed RoB:   from confounding, *and*   from selection bias | | For Yes, must also have assessed RoB:   methods used to ascertain exposures and outcomes, *and*   selection of the reported result from among multiple measurements or analyses of a specified | |  Yes   Partial Yes   No x Includes only RCTs | | |
| Comments: | | | | | | | | |

| **10. Did the review authors report outcomes on the sources of funding for the studies included in the review?** | | | | | | |
| --- | --- | --- | --- | --- | --- | --- |
| For Yes:   Must have reported on the sources of funding for individual studies included in the review.  Note: Reporting that the reviewers looked for this information but it was not reported by study authors also qualifies. | | | | x Yes   No |  |  |
| Comments: | | | |  |  |  |
| **11. If meta-analysis was performed did the review authors use appropriate methods for statistical combination of results?** | | | | | | |
|  | **RCTs**  For Yes:   The authors justified combining the data in a meta-analysis   AND they used an appropriate weighted technique to combine study results and adjusted for heterogeneity if present.   AND investigated the causes of any heterogeneity | |  Yes   No  x No meta-analysis conducted | | |  |
|  | **For NRSI**  For Yes:   The authors justified combining the data in a meta-analysis   AND they used an appropriate weighted technique to combine study results, adjusting for heterogeneity if present   AND they statistically combined effect estimates from NRSI that were adjusted for confounding, rather than combining raw data, or justified combining raw data when adjusted effect estimates were not available   AND they reported separate summary estimates for RCTs and NRSI separately when both were included in the review | |  Yes   No   No meta-analysis conducted | | |  |
|  | Comments: | | | | |  |
| **12. If meta-analysis was performed, did the review authors assess the potential impact of RoB in individual studies on the results of the meta-analysis or other evidence synthesis?** | | | | | | |
|  | For Yes:   included only low risk of bias RCTs   OR, if the pooled estimate was based on RCTs and/or NRSI at variable RoB, the authors performed analyses to investigate possible impact of RoB on summary estimates of effect. | |  Yes   No  x No meta-analysis conducted | | |  |
|  | Comments: | | | | |  |
| **13. Did the review authors account for RoB in individual studies when interpreting/ discussing the results of the review?** | | | | | | |
|  | For Yes:   included only low risk of bias RCTs  x OR, if RCTs with moderate or high RoB, or NRSI were included the review provided a discussion of the likely impact of RoB on the results | | x Yes   No | | |  |
|  | Comments: | | | | |  |
| **14. Did the review authors provide a satisfactory explanation for, and discussion of, any heterogeneity observed in the results of the review?** | | | | | | |
|  | For Yes:   There was no significant heterogeneity in the results  x OR if heterogeneity was present the authors performed an investigation of sources of any heterogeneity in the results and discussed the impact of this on the results of the review | | x Yes   No | | |  |
|  | Comments: | | | | |  |
| **15. If they performed quantitative synthesis did the review authors carry out an adequate investigation of publication bias (small study bias) and discuss its likely impact on the results of the review?** | | | | | | |
|  | For Yes:   performed graphical or statistical tests for publication bias and discussed the likelihood and magnitude of impact of publication bias | |  Yes   No  x No meta-analysis conducted | | |  |
|  | Comments: | | | | |  |
| **16. Did the review authors report any potential sources of conflict of interest, including any funding they received for conducting the review?** | | | | | | |
|  | For Yes:  x The authors reported no competing interests OR   The authors described their funding sources and how they managed potential conflicts of interest | x Yes   No | | | |  |
|  | Comments: | | | | |  |

# Study 29 – Trettel 2017

| **1. Did the research questions and inclusion criteria for the review include the components of PICO?** | | | | | | | | |
| --- | --- | --- | --- | --- | --- | --- | --- | --- |
| For Yes:  x Population  x Intervention  x Comparator group  x Outcome | | | Optional (recommended)   Timeframe for follow-up | | x | | Yes No |  |
| Comments: | | | | | | | |  |
| **2. Did the report of the review contain an explicit statement that the review methods were established prior to the conduct of the review and did the report justify any significant deviations from the protocol?** | | | | | | | | |
|  | For Partial Yes:  The authors state that they had a written protocol or guide that included ALL the following:  x review question(s)  x a search strategy  x inclusion/exclusion criteria   a risk of bias assessment | | For Yes:  As for partial yes, plus the protocol should be registered and should also have specified:   a meta-analysis/synthesis plan, if appropriate, *and*   a plan for investigating causes of heterogeneity   justification for any deviations from the protocol | |     x | | Yes  Partial Yes  No |  |
|  | Comments: | | | | | | |  |
| **3. Did the review authors explain their selection of the study designs for inclusion in the review?** | | | | | | | | |
|  | For Yes, the review should satisfy ONE of the following:   *Explanation for* including only RCTs   OR *Explanation for* including only NRSI   OR *Explanation for* including both RCTs and NRSI | | | | x | | Yes No |  |
|  | Comments: | | | | | | |  |
| **4. Did the review authors use a comprehensive literature search strategy?** | | | | | | | | |
|  | For Partial Yes (all the following): | | For Yes, should also have (all the following):  x searched the reference lists / bibliographies of included studies   searched trial/study registries   included/consulted content experts in the field  x where relevant, searched for grey literature  x conducted search within 24 months of completion of the review | |  | |  |  |
|  | x searched at least 2 databases (relevant to research question)  x provided key word and/or search strategy  x justified publication restrictions | |  |  |   x  | | Yes  Partial Yes No |  |
|  | (e.g. language) | |  |  |  | |  |  |
|  | Comments: | | | | | | |  |
|  | **5. Did the review authors perform study selection in duplicate?** | | | |  | | |  |
|  | For Yes, either ONE of the following:   at least two reviewers independently agreed on selection of eligible studies and achieved consensus on which studies to include   OR two reviewers selected a sample of eligible studies and achieved good agreement (at least 80 percent), with the remainder selected by one reviewer. | | | | x | | Yes NoN/A |  |
|  | Comments: | | | | | | |  |
| **6. Did the review authors perform data extraction in duplicate?** | | | | | | | | |
| For Yes, either ONE of the following:   at least two reviewers achieved consensus on which data to extract from included studies   OR two reviewers extracted data from a sample of eligible studies and achieved good agreement (at least 80 percent), with the remainder extracted by one reviewer. | | | | | |  Yes  x No N/A | | |
| Comments: | | | | | | | | |
| **7. Did the review authors provide a list of excluded studies and justify the exclusions?** | | | | | | | | |
|  | | For Partial Yes:   provided a list of all potentially relevant studies that were read in full-text form but excluded from the review | | For Yes, must also have:   Justified the exclusion from the review of each potentially relevant study | |  Yes   Partial Yes  x No | | |
|  | | Comments: | | | | | | |
| **8. Did the review authors describe the included studies in adequate detail?** | | | | | | | | |
|  | | For Partial Yes (ALL the following):  x described populations  x described interventions  x described comparators  x described outcomes   described research designs | | For Yes, should also have ALL the following:   described population in detail   described intervention in detail (including doses where relevant)   described comparator in detail (including doses where relevant)   described study’s setting   timeframe for follow-up | |  Yes   Partial Yes  x No | | |
|  | | Comments: | | | | | | |
| **9. Did the review authors use a satisfactory technique for assessing the risk of bias (RoB) in individual studies that were included in the review?** | | | | | | | | |
|  | | **RCTs**  For Partial Yes, must have assessed RoB from   unconcealed allocation, *and*   lack of blinding of patients and assessors when assessing outcomes (unnecessary for objective outcomes such as all-cause mortality) | | For Yes, must also have assessed RoB from:   allocation sequence that was not truly random, *and*   selection of the reported result from among multiple measurements or analyses of a specified outcome | |  Yes   Partial Yes  x No   Includes only NRSI | | |
|  | | **NRSI**  For Partial Yes, must have assessed RoB:   from confounding, *and*   from selection bias | | For Yes, must also have assessed RoB:   methods used to ascertain exposures and outcomes, *and*   selection of the reported result from among multiple measurements or analyses of a specified | |  Yes   Partial Yes  x No  Includes only RCTs | | |
| Comments: | | | | | | | | |

| **10. Did the review authors report outcomes on the sources of funding for the studies included in the review?** | | | | | | |
| --- | --- | --- | --- | --- | --- | --- |
| For Yes:   Must have reported on the sources of funding for individual studies included in the review.  Note: Reporting that the reviewers looked for this information but it was not reported by study authors also qualifies. | | | |  Yes  x No |  |  |
| Comments: | | | |  |  |  |
| **11. If meta-analysis was performed did the review authors use appropriate methods for statistical combination of results?** | | | | | | |
|  | **RCTs**  For Yes:   The authors justified combining the data in a meta-analysis   AND they used an appropriate weighted technique to combine study results and adjusted for heterogeneity if present.   AND investigated the causes of any heterogeneity | |  Yes   No  x No meta-analysis conducted | | |  |
|  | **For NRSI**  For Yes:   The authors justified combining the data in a meta-analysis   AND they used an appropriate weighted technique to combine study results, adjusting for heterogeneity if present   AND they statistically combined effect estimates from NRSI that were adjusted for confounding, rather than combining raw data, or justified combining raw data when adjusted effect estimates were not available   AND they reported separate summary estimates for RCTs and NRSI separately when both were included in the review | |  Yes   No  x No meta-analysis conducted | | |  |
|  | Comments: | | | | |  |
| **12. If meta-analysis was performed, did the review authors assess the potential impact of RoB in individual studies on the results of the meta-analysis or other evidence synthesis?** | | | | | | |
|  | For Yes:   included only low risk of bias RCTs   OR, if the pooled estimate was based on RCTs and/or NRSI at variable RoB, the authors performed analyses to investigate possible impact of RoB on summary estimates of effect. | |  Yes   No  x No meta-analysis conducted | | |  |
|  | Comments: | | | | |  |
| **13. Did the review authors account for RoB in individual studies when interpreting/ discussing the results of the review?** | | | | | | |
|  | For Yes:   included only low risk of bias RCTs   OR, if RCTs with moderate or high RoB, or NRSI were included the review provided a discussion of the likely impact of RoB on the results | |  Yes  x No | | |  |
|  | Comments: | | | | |  |
| **14. Did the review authors provide a satisfactory explanation for, and discussion of, any heterogeneity observed in the results of the review?** | | | | | | |
|  | For Yes:   There was no significant heterogeneity in the results   OR if heterogeneity was present the authors performed an investigation of sources of any heterogeneity in the results and discussed the impact of this on the results of the review | |  Yes  x No | | |  |
|  | Comments: | | | | |  |
| **15. If they performed quantitative synthesis did the review authors carry out an adequate investigation of publication bias (small study bias) and discuss its likely impact on the results of the review?** | | | | | | |
|  | For Yes:   performed graphical or statistical tests for publication bias and discussed the likelihood and magnitude of impact of publication bias | |  Yes   No  x No meta-analysis conducted | | |  |
|  | Comments: | | | | |  |
| **16. Did the review authors report any potential sources of conflict of interest, including any funding they received for conducting the review?** | | | | | | |
|  | For Yes:  x The authors reported no competing interests OR   The authors described their funding sources and how they managed potential conflicts of interest | x Yes   No | | | |  |
|  | Comments: | | | | |  |

# Study 30 – Udsen et al

| **1. Did the research questions and inclusion criteria for the review include the components of PICO?** | | | | | | | | |
| --- | --- | --- | --- | --- | --- | --- | --- | --- |
| For Yes:  x Population  x Intervention  x Comparator group  x Outcome | | | Optional (recommended)  x Timeframe for follow-up | | x | | Yes No |  |
| Comments: | | | | | | | |  |
| **2. Did the report of the review contain an explicit statement that the review methods were established prior to the conduct of the review and did the report justify any significant deviations from the protocol?** | | | | | | | | |
|  | For Partial Yes:  The authors state that they had a written protocol or guide that included ALL the following:  x review question(s)  x a search strategy  x inclusion/exclusion criteria  x a risk of bias assessment | | For Yes:  As for partial yes, plus the protocol should be registered and should also have specified:   a meta-analysis/synthesis plan, if appropriate, *and*   a plan for investigating causes of heterogeneity   justification for any deviations from the protocol | |   x | | Yes  Partial Yes  No |  |
|  | Comments: | | | | | | |  |
| **3. Did the review authors explain their selection of the study designs for inclusion in the review?** | | | | | | | | |
|  | For Yes, the review should satisfy ONE of the following:   *Explanation for* including only RCTs   OR *Explanation for* including only NRSI  x OR *Explanation for* including both RCTs and NRSI | | | | x | | Yes No |  |
|  | Comments: | | | | | | |  |
| **4. Did the review authors use a comprehensive literature search strategy?** | | | | | | | | |
|  | For Partial Yes (all the following): | | For Yes, should also have (all the following):  x searched the reference lists / bibliographies of included studies   searched trial/study registries   included/consulted content experts in the field  x where relevant, searched for grey literature   conducted search within 24 months of completion of the review | |  | |  |  |
|  | x searched at least 2 databases (relevant to research question)  x provided key word and/or search strategy  x justified publication restrictions | |  |  |   x  | | Yes  Partial Yes No |  |
|  | (e.g. language) | |  |  |  | |  |  |
|  | Comments: | | | | | | |  |
|  | **5. Did the review authors perform study selection in duplicate?** | | | |  | | |  |
|  | For Yes, either ONE of the following:  x at least two reviewers independently agreed on selection of eligible studies and achieved consensus on which studies to include   OR two reviewers selected a sample of eligible studies and achieved good agreement (at least 80 percent), with the remainder selected by one reviewer. | | | | x  | | Yes No |  |
|  | Comments: | | | | | | |  |
| **6. Did the review authors perform data extraction in duplicate?** | | | | | | | | |
| For Yes, either ONE of the following:  x at least two reviewers achieved consensus on which data to extract from included studies   OR two reviewers extracted data from a sample of eligible studies and achieved good agreement (at least 80 percent), with the remainder extracted by one reviewer. | | | | | | x Yes   No | | |
| Comments: | | | | | | | | |
| **7. Did the review authors provide a list of excluded studies and justify the exclusions?** | | | | | | | | |
|  | | For Partial Yes:   provided a list of all potentially relevant studies that were read in full-text form but excluded from the review | | For Yes, must also have:   Justified the exclusion from the review of each potentially relevant study | |  Yes   Partial Yes  x No | | |
|  | | Comments: | | | | | | |
| **8. Did the review authors describe the included studies in adequate detail?** | | | | | | | | |
|  | | For Partial Yes (ALL the following):  x described populations  x described interventions  x described comparators  x described outcomes  x described research designs | | For Yes, should also have ALL the following:  x described population in detail  x described intervention in detail (including doses where relevant)  x described comparator in detail (including doses where relevant)  x described study’s setting  x timeframe for follow-up | | x Yes   Partial Yes   No | | |
|  | | Comments: | | | | | | |
| **9. Did the review authors use a satisfactory technique for assessing the risk of bias (RoB) in individual studies that were included in the review?** | | | | | | | | |
|  | | **RCTs**  For Partial Yes, must have assessed RoB from   unconcealed allocation, *and*   lack of blinding of patients and assessors when assessing outcomes (unnecessary for objective outcomes such as all-cause mortality) | | For Yes, must also have assessed RoB from:   allocation sequence that was not truly random, *and*   selection of the reported result from among multiple measurements or analyses of a specified outcome | |  Yes   Partial Yes  x No N/A   Includes only NRSI | | |
|  | | **NRSI**  For Partial Yes, must have assessed RoB:   from confounding, *and*   from selection bias | | For Yes, must also have assessed RoB:   methods used to ascertain exposures and outcomes, *and*   selection of the reported result from among multiple measurements or analyses of a specified | |  Yes   Partial Yes  x No N/A   Includes only RCTs | | |
| Comments: | | | | | | | | |

| **10. Did the review authors report outcomes on the sources of funding for the studies included in the review?** | | | | | | |
| --- | --- | --- | --- | --- | --- | --- |
| For Yes:   Must have reported on the sources of funding for individual studies included in the review.  Note: Reporting that the reviewers looked for this information but it was not reported by study authors also qualifies. | | | |  Yes  x No |  |  |
| Comments: | | | |  |  |  |
| **11. If meta-analysis was performed did the review authors use appropriate methods for statistical combination of results?** | | | | | | |
|  | **RCTs**  For Yes:   The authors justified combining the data in a meta-analysis   AND they used an appropriate weighted technique to combine study results and adjusted for heterogeneity if present.   AND investigated the causes of any heterogeneity | |  Yes   No  x No meta-analysis conducted | | |  |
|  | **For NRSI**  For Yes:   The authors justified combining the data in a meta-analysis   AND they used an appropriate weighted technique to combine study results, adjusting for heterogeneity if present   AND they statistically combined effect estimates from NRSI that were adjusted for confounding, rather than combining raw data, or justified combining raw data when adjusted effect estimates were not available   AND they reported separate summary estimates for RCTs and NRSI separately when both were included in the review | |  Yes   No  x No meta-analysis conducted | | |  |
|  | Comments: | | | | |  |
| **12. If meta-analysis was performed, did the review authors assess the potential impact of RoB in individual studies on the results of the meta-analysis or other evidence synthesis?** | | | | | | |
|  | For Yes:   included only low risk of bias RCTs   OR, if the pooled estimate was based on RCTs and/or NRSI at variable RoB, the authors performed analyses to investigate possible impact of RoB on summary estimates of effect. | |  Yes   No  x No meta-analysis conducted | | |  |
|  | Comments: | | | | |  |
| **13. Did the review authors account for RoB in individual studies when interpreting/ discussing the results of the review?** | | | | | | |
|  | For Yes:   included only low risk of bias RCTs   OR, if RCTs with moderate or high RoB, or NRSI were included the review provided a discussion of the likely impact of RoB on the results | |  Yes  x No N/A | | |  |
|  | Comments: | | | | |  |
| **14. Did the review authors provide a satisfactory explanation for, and discussion of, any heterogeneity observed in the results of the review?** | | | | | | |
|  | For Yes:  x There was no significant heterogeneity in the results   OR if heterogeneity was present the authors performed an investigation of sources of any heterogeneity in the results and discussed the impact of this on the results of the review | | x Yes   No | | |  |
|  | Comments: | | | | |  |
| **15. If they performed quantitative synthesis did the review authors carry out an adequate investigation of publication bias (small study bias) and discuss its likely impact on the results of the review?** | | | | | | |
|  | For Yes:   performed graphical or statistical tests for publication bias and discussed the likelihood and magnitude of impact of publication bias | |  Yes   No  x No meta-analysis conducted | | |  |
|  | Comments: | | | | |  |
| **16. Did the review authors report any potential sources of conflict of interest, including any funding they received for conducting the review?** | | | | | | |
|  | For Yes:   The authors reported no competing interests OR   The authors described their funding sources and how they managed potential conflicts of interest |  Yes  x No | | | |  |
|  | Comments: | | | | |  |

# Study 31 – Verma et al

| **1. Did the research questions and inclusion criteria for the review include the components of PICO?** | | | | | | | | |
| --- | --- | --- | --- | --- | --- | --- | --- | --- |
| For Yes:  x Population  x Intervention   Comparator group  x Outcome | | | Optional (recommended)   Timeframe for follow-up | | x | | Yes No |  |
| Comments: | | | | | | | |  |
| **2. Did the report of the review contain an explicit statement that the review methods were established prior to the conduct of the review and did the report justify any significant deviations from the protocol?** | | | | | | | | |
|  | For Partial Yes:  The authors state that they had a written protocol or guide that included ALL the following:  x review question(s)  x a search strategy  x inclusion/exclusion criteria   a risk of bias assessment | | For Yes:  As for partial yes, plus the protocol should be registered and should also have specified:   a meta-analysis/synthesis plan, if appropriate, *and*   a plan for investigating causes of heterogeneity   justification for any deviations from the protocol | |     x | | Yes  Partial Yes  No |  |
|  | Comments: | | | | | | |  |
| **3. Did the review authors explain their selection of the study designs for inclusion in the review?** | | | | | | | | |
|  | For Yes, the review should satisfy ONE of the following:   *Explanation for* including only RCTs   OR *Explanation for* including only NRSI  x OR *Explanation for* including both RCTs and NRSI | | | | x | | Yes No |  |
|  | Comments: | | | | | | |  |
| **4. Did the review authors use a comprehensive literature search strategy?** | | | | | | | | |
|  | For Partial Yes (all the following): | | For Yes, should also have (all the following):  x searched the reference lists / bibliographies of included studies   searched trial/study registries   included/consulted content experts in the field   where relevant, searched for grey literature   conducted search within 24 months of completion of the review | |  | |  |  |
|  | x searched at least 2 databases (relevant to research question)  x provided key word and/or search strategy  x justified publication restrictions | |  |  |   x  | | Yes  Partial Yes No |  |
|  | (e.g. language) | |  |  |  | |  |  |
|  | Comments: | | | | | | |  |
|  | **5. Did the review authors perform study selection in duplicate?** | | | |  | | |  |
|  | For Yes, either ONE of the following:  x at least two reviewers independently agreed on selection of eligible studies and achieved consensus on which studies to include   OR two reviewers selected a sample of eligible studies and achieved good agreement (at least 80 percent), with the remainder selected by one reviewer. | | | | x | | Yes No |  |
|  | Comments: | | | | | | |  |
| **6. Did the review authors perform data extraction in duplicate?** | | | | | | | | |
| For Yes, either ONE of the following:  x at least two reviewers achieved consensus on which data to extract from included studies   OR two reviewers extracted data from a sample of eligible studies and achieved good agreement (at least 80 percent), with the remainder extracted by one reviewer. | | | | | | x Yes   No | | |
| Comments: | | | | | | | | |
| **7. Did the review authors provide a list of excluded studies and justify the exclusions?** | | | | | | | | |
|  | | For Partial Yes:   provided a list of all potentially relevant studies that were read in full-text form but excluded from the review | | For Yes, must also have:   Justified the exclusion from the review of each potentially relevant study | |  Yes   Partial Yes  x No | | |
|  | | Comments: | | | | | | |
| **8. Did the review authors describe the included studies in adequate detail?** | | | | | | | | |
|  | | For Partial Yes (ALL the following):  x described populations  x described interventions   described comparators  x described outcomes  x described research designs | | For Yes, should also have ALL the following:   described population in detail   described intervention in detail (including doses where relevant)   described comparator in detail (including doses where relevant)   described study’s setting   timeframe for follow-up | |  Yes   Partial Yes  x No | | |
|  | | Comments: | | | | | | |
| **9. Did the review authors use a satisfactory technique for assessing the risk of bias (RoB) in individual studies that were included in the review?** | | | | | | | | |
|  | | **RCTs**  For Partial Yes, must have assessed RoB from   unconcealed allocation, *and*   lack of blinding of patients and assessors when assessing outcomes (unnecessary for objective outcomes such as all-cause mortality) | | For Yes, must also have assessed RoB from:   allocation sequence that was not truly random, *and*   selection of the reported result from among multiple measurements or analyses of a specified outcome | |  Yes   Partial Yes  x No   Includes only NRSI | | |
|  | | **NRSI**  For Partial Yes, must have assessed RoB:   from confounding, *and*   from selection bias | | For Yes, must also have assessed RoB:   methods used to ascertain exposures and outcomes, *and*   selection of the reported result from among multiple measurements or analyses of a specified | |  Yes   Partial Yes  x No  Includes only RCTs | | |
| Comments: | | | | | | | | |

| **10. Did the review authors report outcomes on the sources of funding for the studies included in the review?** | | | | | | |
| --- | --- | --- | --- | --- | --- | --- |
| For Yes:  x Must have reported on the sources of funding for individual studies included in the review.  Note: Reporting that the reviewers looked for this information but it was not reported by study authors also qualifies. | | | | x Yes   No |  |  |
| Comments: | | | |  |  |  |
| **11. If meta-analysis was performed did the review authors use appropriate methods for statistical combination of results?** | | | | | | |
|  | **RCTs**  For Yes:   The authors justified combining the data in a meta-analysis   AND they used an appropriate weighted technique to combine study results and adjusted for heterogeneity if present.   AND investigated the causes of any heterogeneity | |  Yes   No  x No meta-analysis conducted | | |  |
|  | **For NRSI**  For Yes:   The authors justified combining the data in a meta-analysis   AND they used an appropriate weighted technique to combine study results, adjusting for heterogeneity if present   AND they statistically combined effect estimates from NRSI that were adjusted for confounding, rather than combining raw data, or justified combining raw data when adjusted effect estimates were not available   AND they reported separate summary estimates for RCTs and NRSI separately when both were included in the review | |  Yes   No  x No meta-analysis conducted | | |  |
|  | Comments: | | | | |  |
| **12. If meta-analysis was performed, did the review authors assess the potential impact of RoB in individual studies on the results of the meta-analysis or other evidence synthesis?** | | | | | | |
|  | For Yes:   included only low risk of bias RCTs   OR, if the pooled estimate was based on RCTs and/or NRSI at variable RoB, the authors performed analyses to investigate possible impact of RoB on summary estimates of effect. | |  Yes   No  x No meta-analysis conducted | | |  |
|  | Comments: | | | | |  |
| **13. Did the review authors account for RoB in individual studies when interpreting/ discussing the results of the review?** | | | | | | |
|  | For Yes:   included only low risk of bias RCTs   OR, if RCTs with moderate or high RoB, or NRSI were included the review provided a discussion of the likely impact of RoB on the results | |  Yes  x No | | |  |
|  | Comments: | | | | |  |
| **14. Did the review authors provide a satisfactory explanation for, and discussion of, any heterogeneity observed in the results of the review?** | | | | | | |
|  | For Yes:   There was no significant heterogeneity in the results   OR if heterogeneity was present the authors performed an investigation of sources of any heterogeneity in the results and discussed the impact of this on the results of the review | |  Yes  x No | | |  |
|  | Comments: | | | | |  |
| **15. If they performed quantitative synthesis did the review authors carry out an adequate investigation of publication bias (small study bias) and discuss its likely impact on the results of the review?** | | | | | | |
|  | For Yes:   performed graphical or statistical tests for publication bias and discussed the likelihood and magnitude of impact of publication bias | |  Yes   No  x No meta-analysis conducted | | |  |
|  | Comments: | | | | |  |
| **16. Did the review authors report any potential sources of conflict of interest, including any funding they received for conducting the review?** | | | | | | |
|  | For Yes:  x The authors reported no competing interests OR   The authors described their funding sources and how they managed potential conflicts of interest | x Yes   No | | | |  |
|  | Comments: | | | | |  |

# Study 32 - Willard et al

| **1. Did the research questions and inclusion criteria for the review include the components of PICO?** | | | | | | | | |
| --- | --- | --- | --- | --- | --- | --- | --- | --- |
| For Yes:  x  Population  x  Intervention   Comparator group   Outcome | | | Optional (recommended)   Timeframe for follow-up | | x | | Yes No |  |
| Comments: | | | | | | | |  |
| **2. Did the report of the review contain an explicit statement that the review methods were established prior to the conduct of the review and did the report justify any significant deviations from the protocol?** | | | | | | | | |
|  | For Partial Yes:  The authors state that they had a written protocol or guide that included ALL the following:  x review question(s)  x a search strategy  x inclusion/exclusion criteria   a risk of bias assessment | | For Yes:  As for partial yes, plus the protocol should be registered and should also have specified:   a meta-analysis/synthesis plan, if appropriate, *and*   a plan for investigating causes of heterogeneity   justification for any deviations from the protocol | |   x | | Yes  Partial Yes  No |  |
|  | Comments: | | | | | | |  |
| **3. Did the review authors explain their selection of the study designs for inclusion in the review?** | | | | | | | | |
|  | For Yes, the review should satisfy ONE of the following:   *Explanation for* including only RCTs   OR *Explanation for* including only NRSI   OR *Explanation for* including both RCTs and NRSI | | | | x | | Yes No |  |
|  | Comments: | | | | | | |  |
| **4. Did the review authors use a comprehensive literature search strategy?** | | | | | | | | |
|  | For Partial Yes (all the following): | | For Yes, should also have (all the following):  x searched the reference lists / bibliographies of included studies   searched trial/study registries   included/consulted content experts in the field  x where relevant, searched for grey literature   conducted search within 24 months of completion of the review | |  | |  |  |
|  | x searched at least 2 databases (relevant to research question)  x provided key word and/or search strategy  x justified publication restrictions | |  |  |   x  | | Yes  Partial Yes No |  |
|  | (e.g. language) | |  |  |  | |  |  |
|  | Comments: | | | | | | |  |
|  | **5. Did the review authors perform study selection in duplicate?** | | | |  | | |  |
|  | For Yes, either ONE of the following:  x at least two reviewers independently agreed on selection of eligible studies and achieved consensus on which studies to include   OR two reviewers selected a sample of eligible studies and achieved good agreement (at least 80 percent), with the remainder selected by one reviewer. | | | | x | | Yes No |  |
|  | Comments: | | | | | | |  |
| **6. Did the review authors perform data extraction in duplicate?** | | | | | | | | |
| For Yes, either ONE of the following:  x at least two reviewers achieved consensus on which data to extract from included studies   OR two reviewers extracted data from a sample of eligible studies and achieved good agreement (at least 80 percent), with the remainder extracted by one reviewer. | | | | | | x Yes   No | | |
| Comments: | | | | | | | | |
| **7. Did the review authors provide a list of excluded studies and justify the exclusions?** | | | | | | | | |
|  | | For Partial Yes:   provided a list of all potentially relevant studies that were read in full-text form but excluded from the review | | For Yes, must also have:   Justified the exclusion from the review of each potentially relevant study | |  Yes   Partial Yes  x No | | |
|  | | Comments: | | | | | | |
| **8. Did the review authors describe the included studies in adequate detail?** | | | | | | | | |
|  | | For Partial Yes (ALL the following):   described populations   described interventions   described comparators   described outcomes   described research designs | | For Yes, should also have ALL the following:   described population in detail   described intervention in detail (including doses where relevant)   described comparator in detail (including doses where relevant)   described study’s setting   timeframe for follow-up | |  Yes   Partial Yes  x No | | |
|  | | Comments: | | | | | | |
| **9. Did the review authors use a satisfactory technique for assessing the risk of bias (RoB) in individual studies that were included in the review?** | | | | | | | | |
|  | | **RCTs**  For Partial Yes, must have assessed RoB from   unconcealed allocation, *and*   lack of blinding of patients and assessors when assessing outcomes (unnecessary for objective outcomes such as all-cause mortality) | | For Yes, must also have assessed RoB from:   allocation sequence that was not truly random, *and*   selection of the reported result from among multiple measurements or analyses of a specified outcome | |  Yes   Partial Yes  x No   Includes only NRSI | | |
|  | | **NRSI**  For Partial Yes, must have assessed RoB:   from confounding, *and*   from selection bias | | For Yes, must also have assessed RoB:   methods used to ascertain exposures and outcomes, *and*   selection of the reported result from among multiple measurements or analyses of a specified | |  Yes   Partial Yes  x No  Includes only RCTs | | |
| Comments: | | | | | | | | |

| **10. Did the review authors report outcomes on the sources of funding for the studies included in the review?** | | | | | | |
| --- | --- | --- | --- | --- | --- | --- |
| For Yes:   Must have reported on the sources of funding for individual studies included in the review.  Note: Reporting that the reviewers looked for this information but it was not reported by study authors also qualifies. | | | |  Yes  x No |  |  |
| Comments: | | | |  |  |  |
| **11. If meta-analysis was performed did the review authors use appropriate methods for statistical combination of results?** | | | | | | |
|  | **RCTs**  For Yes:   The authors justified combining the data in a meta-analysis   AND they used an appropriate weighted technique to combine study results and adjusted for heterogeneity if present.   AND investigated the causes of any heterogeneity | |  Yes   No  x No meta-analysis conducted | | |  |
|  | **For NRSI**  For Yes:   The authors justified combining the data in a meta-analysis   AND they used an appropriate weighted technique to combine study results, adjusting for heterogeneity if present   AND they statistically combined effect estimates from NRSI that were adjusted for confounding, rather than combining raw data, or justified combining raw data when adjusted effect estimates were not available   AND they reported separate summary estimates for RCTs and NRSI separately when both were included in the review | |  Yes   No  x No meta-analysis conducted | | |  |
|  | Comments: | | | | |  |
| **12. If meta-analysis was performed, did the review authors assess the potential impact of RoB in individual studies on the results of the meta-analysis or other evidence synthesis?** | | | | | | |
|  | For Yes:   included only low risk of bias RCTs   OR, if the pooled estimate was based on RCTs and/or NRSI at variable RoB, the authors performed analyses to investigate possible impact of RoB on summary estimates of effect. | |  Yes   No  x No meta-analysis conducted | | |  |
|  | Comments: | | | | |  |
| **13. Did the review authors account for RoB in individual studies when interpreting/ discussing the results of the review?** | | | | | | |
|  | For Yes:   included only low risk of bias RCTs   OR, if RCTs with moderate or high RoB, or NRSI were included the review provided a discussion of the likely impact of RoB on the results | |  Yes  x No | | |  |
|  | Comments: | | | | |  |
| **14. Did the review authors provide a satisfactory explanation for, and discussion of, any heterogeneity observed in the results of the review?** | | | | | | |
|  | For Yes:   There was no significant heterogeneity in the results   OR if heterogeneity was present the authors performed an investigation of sources of any heterogeneity in the results and discussed the impact of this on the results of the review | |  Yes  x No | | |  |
|  | Comments: | | | | |  |
| **15. If they performed quantitative synthesis did the review authors carry out an adequate investigation of publication bias (small study bias) and discuss its likely impact on the results of the review?** | | | | | | |
|  | For Yes:   performed graphical or statistical tests for publication bias and discussed the likelihood and magnitude of impact of publication bias | |  Yes   No  x No meta-analysis conducted | | |  |
|  | Comments: | | | | |  |
| **16. Did the review authors report any potential sources of conflict of interest, including any funding they received for conducting the review?** | | | | | | |
|  | For Yes:   The authors reported no competing interests OR  x The authors described their funding sources and how they managed potential conflicts of interest | x Yes   No | | | |  |
|  | Comments: | | | | |  |

# Study 33 - Zanin et al

| **1. Did the research questions and inclusion criteria for the review include the components of PICO?** | | | | | | | | |
| --- | --- | --- | --- | --- | --- | --- | --- | --- |
| For Yes:  x  Population  x Intervention  x  Comparator group  x  Outcome | | | Optional (recommended)   Timeframe for follow-up | | x | | Yes No |  |
| Comments: | | | | | | | |  |
| **2. Did the report of the review contain an explicit statement that the review methods were established prior to the conduct of the review and did the report justify any significant deviations from the protocol?** | | | | | | | | |
|  | For Partial Yes:  The authors state that they had a written protocol or guide that included ALL the following:  x review question(s)  x a search strategy  x inclusion/exclusion criteria  x a risk of bias assessment | | For Yes:  As for partial yes, plus the protocol should be registered and should also have specified:   a meta-analysis/synthesis plan, if appropriate, *and*   a plan for investigating causes of heterogeneity   justification for any deviations from the protocol | |   x | | Yes  Partial Yes  No |  |
|  | Comments: | | | | | | |  |
| **3. Did the review authors explain their selection of the study designs for inclusion in the review?** | | | | | | | | |
|  | For Yes, the review should satisfy ONE of the following:   *Explanation for* including only RCTs   OR *Explanation for* including only NRSI  x OR *Explanation for* including both RCTs and NRSI | | | | x | | Yes No |  |
|  | Comments: | | | | | | |  |
| **4. Did the review authors use a comprehensive literature search strategy?** | | | | | | | | |
|  | For Partial Yes (all the following): | | For Yes, should also have (all the following):   searched the reference lists / bibliographies of included studies  x searched trial/study registries  x included/consulted content experts in the field   where relevant, searched for grey literature   conducted search within 24 months of completion of the review | |  | |  |  |
|  | x searched at least 2 databases (relevant to research question)  x provided key word and/or search strategy  x justified publication restrictions | |  |  |   x  | | Yes  Partial Yes No |  |
|  | (e.g. language) | |  |  |  | |  |  |
|  | Comments: | | | | | | |  |
|  | **5. Did the review authors perform study selection in duplicate?** | | | |  | | |  |
|  | For Yes, either ONE of the following:  x at least two reviewers independently agreed on selection of eligible studies and achieved consensus on which studies to include   OR two reviewers selected a sample of eligible studies and achieved good agreement (at least 80 percent), with the remainder selected by one reviewer. | | | | x | | Yes No |  |
|  | Comments: | | | | | | |  |
| **6. Did the review authors perform data extraction in duplicate?** | | | | | | | | |
| For Yes, either ONE of the following:  x at least two reviewers achieved consensus on which data to extract from included studies   OR two reviewers extracted data from a sample of eligible studies and achieved good agreement (at least 80 percent), with the remainder extracted by one reviewer. | | | | | | x Yes   No | | |
| Comments: | | | | | | | | |
| **7. Did the review authors provide a list of excluded studies and justify the exclusions?** | | | | | | | | |
|  | | For Partial Yes:   provided a list of all potentially relevant studies that were read in full-text form but excluded from the review | | For Yes, must also have:   Justified the exclusion from the review of each potentially relevant study | |  Yes   Partial Yes  x No | | |
|  | | Comments: | | | | | | |
| **8. Did the review authors describe the included studies in adequate detail?** | | | | | | | | |
|  | | For Partial Yes (ALL the following):  x described populations  x described interventions  x described comparators  x described outcomes  x described research designs | | For Yes, should also have ALL the following:  x described population in detail  x described intervention in detail (including doses where relevant)  x described comparator in detail (including doses where relevant)  x described study’s setting   timeframe for follow-up | |  Yes  x Partial Yes   No | | |
|  | | Comments: | | | | | | |
| **9. Did the review authors use a satisfactory technique for assessing the risk of bias (RoB) in individual studies that were included in the review?** | | | | | | | | |
|  | | **RCTs**  For Partial Yes, must have assessed RoB from  x unconcealed allocation, *and*  x lack of blinding of patients and assessors when assessing outcomes (unnecessary for objective outcomes such as all-cause mortality) | | For Yes, must also have assessed RoB from:   allocation sequence that was not truly random, *and*   selection of the reported result from among multiple measurements or analyses of a specified outcome | |  Yes  x Partial Yes   No   Includes only NRSI | | |
|  | | **NRSI**  For Partial Yes, must have assessed RoB:  x from confounding, *and*  x from selection bias | | For Yes, must also have assessed RoB:   methods used to ascertain exposures and outcomes, *and*   selection of the reported result from among multiple measurements or analyses of a specified | |  Yes  x Partial Yes   No  Includes only RCTs | | |
| Comments: | | | | | | | | |

| **10. Did the review authors report outcomes on the sources of funding for the studies included in the review?** | | | | | | |
| --- | --- | --- | --- | --- | --- | --- |
| For Yes:   Must have reported on the sources of funding for individual studies included in the review.  Note: Reporting that the reviewers looked for this information but it was not reported by study authors also qualifies. | | | |  Yes  x No |  |  |
| Comments: | | | |  |  |  |
| **11. If meta-analysis was performed did the review authors use appropriate methods for statistical combination of results?** | | | | | | |
|  | **RCTs**  For Yes:   The authors justified combining the data in a meta-analysis   AND they used an appropriate weighted technique to combine study results and adjusted for heterogeneity if present.   AND investigated the causes of any heterogeneity | |  Yes   No  x No meta-analysis conducted | | |  |
|  | **For NRSI**  For Yes:   The authors justified combining the data in a meta-analysis   AND they used an appropriate weighted technique to combine study results, adjusting for heterogeneity if present   AND they statistically combined effect estimates from NRSI that were adjusted for confounding, rather than combining raw data, or justified combining raw data when adjusted effect estimates were not available   AND they reported separate summary estimates for RCTs and NRSI separately when both were included in the review | |  Yes   No  x No meta-analysis conducted | | |  |
|  | Comments: | | | | |  |
| **12. If meta-analysis was performed, did the review authors assess the potential impact of RoB in individual studies on the results of the meta-analysis or other evidence synthesis?** | | | | | | |
|  | For Yes:   included only low risk of bias RCTs   OR, if the pooled estimate was based on RCTs and/or NRSI at variable RoB, the authors performed analyses to investigate possible impact of RoB on summary estimates of effect. | |  Yes   No  x No meta-analysis conducted | | |  |
|  | Comments: | | | | |  |
| **13. Did the review authors account for RoB in individual studies when interpreting/ discussing the results of the review?** | | | | | | |
|  | For Yes:   included only low risk of bias RCTs   OR, if RCTs with moderate or high RoB, or NRSI were included the review provided a discussion of the likely impact of RoB on the results | |  Yes  x No | | |  |
|  | Comments: | | | | |  |
| **14. Did the review authors provide a satisfactory explanation for, and discussion of, any heterogeneity observed in the results of the review?** | | | | | | |
|  | For Yes:  x There was no significant heterogeneity in the results   OR if heterogeneity was present the authors performed an investigation of sources of any heterogeneity in the results and discussed the impact of this on the results of the review | | x Yes   No | | |  |
|  | Comments: | | | | |  |
| **15. If they performed quantitative synthesis did the review authors carry out an adequate investigation of publication bias (small study bias) and discuss its likely impact on the results of the review?** | | | | | | |
|  | For Yes:   performed graphical or statistical tests for publication bias and discussed the likelihood and magnitude of impact of publication bias | |  Yes   No  x No meta-analysis conducted | | |  |
|  | Comments: | | | | |  |
| **16. Did the review authors report any potential sources of conflict of interest, including any funding they received for conducting the review?** | | | | | | |
|  | For Yes:  x The authors reported no competing interests OR   The authors described their funding sources and how they managed potential conflicts of interest | x Yes   No | | | |  |
|  | Comments: | | | | |  |
