# Supplementary material for: The Current Status of Telemedicine Technology Use Across the World Health Organization European Region: An Overview of Systematic Reviews
Source: J Med Internet Res. 2022 Oct 27;24(10):e40877. doi: 10.2196/40877 (PMC9650581; doi:10.2196/40877)
Supplement: Multimedia Appendix 5 [file jmir_v24i10e40877_app5.docx]

**Table 1.** Main characteristics of included reviews

| **Review ID** | **Included Countries** | **Objective** | **Telemedicine Specialty** | **Medical Specialty or Medical Focus** | **Setting, Scenario** | **Included Databases** | **Number of Included Studies** |
| --- | --- | --- | --- | --- | --- | --- | --- |
| [21] | Germany | To identify publications related to telemedicine projects in rural areas of Germany and to examine these studies according to scientific aspects of implementation. | Telemedicine in general | Multi-focal study | Rural areas | PubMed, Web of Science, ACM Digital Library and Google Scholar | 16 |
| [22] | Denmark, Italy and the Netherlands | To measure the real effect of pre-hospital triage with telemedicine in case of acute myocardial infarction in a meta- analysis study. | Telecardiology | Chapter IX - Diseases of the circulatory system | Pre-hospital triage/ Emergency Medicine | MEDLINE and PubMed | 11 |
| [23] | Austria, Belgium, Finland, Sweden, Italy and Germany | To identify telehealth initiatives described in the literature as a strategy for national health policies. | Telecardiology | Chapter IX - Diseases of the circulatory system | Home patients | PubMed, CINAHL, The Cochrane Library and Embase | 11 |
| [24] | Italy, the United Kingdom and Germany | To investigate the effectiveness of screen-to-screen therapy via a videoconferencing system compared to conventional face-to-face therapy of adult aphasia patients. | Tele-phonoaudiology | Chapter XVIII - Symptoms, signs and abnormal clinical and laboratory findings, not elsewhere classified | Medical office and home-based | The Cochrane Library, PubMed, The American Speech-Language-Hearing Association and Web of Science | 5 |
| [25] | The United Kingdom, Denmark and the Netherlands | To provide a comprehensive description of the methodologies used in home telemonitoring interventions for chronic obstructive pulmonary disease and to explore patients’ adherence, and satisfaction with the use of telemonitoring systems. | Telemedicine applied to respiratory diseases | Chapter X - Diseases of the respiratory system | In-hospital patients or those receiving specialised care at hospital or at home | MEDLINE, Embase, Web of Science and B-on Online Knowledge Library | 17 |
| [26] | Italy, the United Kingdom, France and Spain | To explore the use, accessibility and feasibility of telemedicine in older adults with dementia, as well as examine the potential mental health impacts of these technologies, by reviewing evidence from studies conducted during COVID-19. | Telepsychiatry and telepsychology | Chapter V - Mental and behavioural disorders | Home-based assessment | PubMed, Scopus and Web of Science | 7 |
| [27] | Belgium, Denmark, the United Kingdom, the Netherlands and Europe in general | To systematically review the economic evaluation studies that compared telemedicine with usual care for cardiovascular patients. | Telecardiology | Chapter IX - Diseases of the circulatory system | Multiple settings | PubMed, Embase, Scopus, Global Health, Google Scholar, Magiran and SID | 20 |
| [28] | European countries only | To examine the provision and efficacy of telehealth for chronic obstructive pulmonary disease management to guide future decision-making. | Telemedicine applied to respiratory diseases | Chapter X - Diseases of the respiratory system | Stable patients as well as patients experiencing exacerbations leading to hospital admission | The Cochrane Library, EBSCOHost CINAHL and Scopus | 12 (Overviews) and 16 (Systematic Reviews) |
| [29] | Poland | To present a review based on the literature and proceedings from selected telemedicine conferences. | Telemedicine apps in general | Multi-focal study | Primary, secondary and tertiary levels | PubMed, Embase and two medical conference proceedings platforms | 129 |
| [30] | The Netherlands | To provide an overview of all eHealth apps used in Dutch chronic obstructive pulmonary disease care and to assess these apps on a number of relevant criteria. | Telemedicine applied to respiratory diseases | Chapter X - Diseases of the respiratory system | Primary, secondary and tertiary levels | PubMed, Google Scholar and eHealth application platforms | 13 |
| [31] | Norway, the Netherlands and the United Kingdom | To assess the extent to which shared decision-making can take place in telemedicine (remote shared decision-making). | Telemedicine in general | Multi-focal study | Primary, secondary and tertiary levels | MEDLINE, Embase, The Cochrane Library, Web of Science and Scopus | 12 |
| [32] | Denmark, Norway and Sweden | To examine the status of research on the use of teleconsultation in chronic wound management. | Tele-endocrionology | Chapter XII - Diseases of the skin and subcutaneous tissue | Not specified | The Cochrane Library and PubMed | 6 |
| [33] | * Austria, Belgium, Denmark, Germany, Greece, Italy, Ireland, the Netherlands, Norway, Sweden and the United Kingdom | To present the state of the art of electronic implementations of patient-reported outcome (ePRO)-based digital health interventions and highlight the contribution of such interventions to palliative cancer care. | Teleoncology | Multi-focal study | Not specified | PubMed and Web of Science | 24 |
| [34] | Denmark | To survey telemedicine services currently in operation across Denmark. The study specifically seeks to answer the following questions: What initiatives are deployed within the different regions? What are the motivations behind the projects? What technologies are being utilised? What medical disciplines are being supported using telemedicine systems? | Telemedicine in general | Multi-focal study | Multiple settings | Telemedicinsk Landkort (a database of telemedicine projects in Denmark hosted by MedCom) | 118 |
| [35] | * Albania, Greece, Iceland, Italy, Russia, Turkey and Ukraine | To answer these questions: (1) What type of studies on providing telehealth services to families of children with neurodevelopmental disabilities in Europe have been conducted in the last five years? (2) What were the main behaviours targeted for change and intervention strategies used? (3) What were the main telehealth components? (4) What barriers were faced? (5) What future work needs to be done to support children and families impacted by neurodevelopmental disabilities in Europe looking to access behavioural services via a telehealth model? | Telepsychiatry and telepsychology | Chapter V - Mental and behavioural disorders | Specialist behavioural interventions for children | Web of Science, Scopus, PsycInfo and Google Scholar | 6 |
| [36] | Greece | To review recently published literature regarding teleophthalmology in Europe, describe screening methods and equipment depending on the examined eye disease and evaluate cost-effectiveness, patients’ compliance with treatment and satisfaction with telemedicine services. | Teleophthalmology | Chapter VII - Diseases of the eye and adnexa | Not specified | PubMed, Google Scholar and Springer | 44 |
| [37] | Italy | To evaluate the role of telerehabilitation in Italy, with regard to the motor and cognitive rehabilitation programmes applied to neurological pathologies, in both paediatric and adult patients. | Teleneurorehabilitation | Chapter VI - Diseases of the nervous system | Not specified | PubMed, Web Of Science and The Cochrane Library | 38 |
| [38] | The United Kingdom, the Netherlands, Italy and Norway | To evaluate the effectiveness and patient-level impact of, and patient and clinician satisfaction with, networked communication technologies associated with meeting the healthcare needs of adolescents and young adults with diagnosed mental health disorders. | Telepsychiatry | Chapter V - Mental and behavioural disorders | Home-based and self-care | MEDLINE, Embase, ASSIA, Sociological Abstract, Social Studies Abstract, PsycInfo, The Cochrane Library, Dissertation Abstracts and Current Controlled Trials | 12 |
| [39] | * The United Kingdom | To conduct a systematic review of evidence of cost-effectiveness of telehealth interventions in the community compared to usual care, and its impact on quality of life. | Telemedicine in general | Multi-focal study | Home-care-based | MEDLINE and CINAHL | 9 |
| [40] | * The United Kingdom, Sweden, Finland, Denmark and Italy | To assess the evidence of delivering e-consultations using secure email, messaging or video links in primary care. | Telemedicine for primary care | Multi-focal study | Primary care and outpatient care settings | MEDLINE, Embase, CINAHL, The Cochrane Library, PsycInfo, EconLit and Web of Science | 52 |
| [41] | * Austria, Belgium, Denmark, Finland, France, Germany, the Netherlands, Norway, Spain, Switzerland and the United Kingdom | To review empirical studies of the use of digitally administered PROs in routine care and examine the stated reasons for patients’ non-use of digital PROs. | Telemedicine in general | Multi-focal study | Multiple settings | PubMed, Embase, Web of Science and PsycInfo | 51 |
| [42] | The United Kingdom | To find out what healthcare settings in the United Kingdom teleconsultations have been used in. | Telemedicine in general | Multi-focal study | General healthcare setting | PubMed, Scopus, The Cochrane Library and CINAHL | 101 |
| [43] | France | To describe regional acute telestroke implementation at a national level in France. | Telestroke | Chapter VI - Diseases of the nervous system | Inter-Hospital (Hub/Spoke) | PubMed and ScienceDirect | 24 |
| [44] | * The United Kingdom, Belgium, Germany, France, Italy, the Netherlands, Finland and Austria | To conduct a systematic review of the available published evidence on the safety, effectiveness and cost-effectiveness of Internet-based device-assisted remote monitoring systems for therapeutic cardiac implantable electronic devices such as pacemakers, implantable cardioverter-defibrillators and cardiac resynchronisation therapy devices. | Telecardiology | Chapter IX - Diseases of the circulatory system | Multiple settings | PubMed, MEDLINE, Embase, CINAHL, The Cochrane Library and the International Agency for Health Technology Assessment | 48 |
| [45] | Norway, the United Kingdom and Denmark | To map a body of literature and to summarise and discuss research findings concerning historical telehealth and digital development over the last 20 years that people 75 years and older in European countries have been part of. Moreover, to identify research gaps in the existing literature in order to inform future research. | Multiple telemedicine specialties | Multi-focal study | Home care, test centre, home surroundings and outdoor environment | Embase, CINAHL, MEDLINE, Scopus and OpenGrey | 13 |
| [46] | The United Kingdom | To conduct a meta-analysis review of the gold standard evidence of the acceptability and clinical effectiveness of e-therapies recommended for use in the National Health Service in the United Kingdom. | Telepsychiatry | Chapter V - Mental and behavioural disorders | Online home-based interventions | PsycInfo, Web of Science and PubMed | 24 |
| [47] | The Netherlands, Denmark, Poland and the United Kingdom | To review the use of telehealth in subjects with diabetic foot ulcers and evaluate its impact on clinical outcomes, diagnostic accuracy, cost-effectiveness and behavioural perceptions. | Tele-endocrinology and Teledermatology | Chapter XII - Diseases of the skin and subcutaneous tissue | Multiple settings | MEDLINE/PubMed and Google Scholar | 11 |
| [48] | The Netherlands, Denmark and Spain | To examine the efficacy of telemedicine-based aftercare interventions for breast cancer survivors regarding specific outcomes of functioning. | Teleoncology | Multi-focal study | Home care or any aftercare facility | PubMed and The Cochrane Library | 11 |
| [49] | The United Kingdom, Spain, the Netherlands, Austria, Italy, Switzerland, Germany, Australia, Turkey, Norway, Sweden, Finland, France, Denmark and Iceland | To identify the use and current status of teledermatology across the world with regard to geographical distribution of published studies, treated indications, research questions, and its reliability in diagnosis and therapy compared to classic face-to-face consultations, by a systematic search of the literature. | Teledermatology | Chapter XII - Diseases of the skin and subcutaneous tissue | Referral, diagnosis or consultation between general practitioner and dermatologist, and monitoring or specialist consultation in home settings | PubMed and Embase | 204 |
| [50] | Denmark | To synthesise the evidence on telehealth for patients with COPD and draw on generally accepted criteria for cost and cost-effectiveness assessment. | Telemedicine applied to respiratory diseases | Chapter X - Diseases of the respiratory system | Home- and hospital-based | PubMed, Embase, The Cochrane Library, CINAHL, Web of Science, NHS Economic Evaluation Database, Scopus, EconLit and Google Scholar | 6 |
| [51] | The United Kingdom, Belgium, Germany, Italy, Norway and Romania | To synthesise the literature exploring patients’ and physicians’ experiences with remote consultations in primary care during the pandemic, with the further aim of informing their future delivery. | Telemedicine for primary care | Multi-focal study | Experiences with remote consultations in primary care | PubMed and PsycInfo | 24 |
| [52] | The Netherlands | To answer the question: Which online care platforms for older adults are available in the Netherlands and what are their characteristics? | Telegerontology | Chapter XXI - Factors influencing health status and contact with health services | Online care platforms for community-dwelling older adults | PubMed and PsycInfo and Google Scholar | 21 platforms - no scientific articles included. 21 documents retrieved from websites or from online descriptions of the included platforms |
| [53] | Italy | To describe the availability, psychometric properties and feasibility of t-NPs tools currently available in Italy. | Teleneuropsychology (t-NPs) | Chapter VI - Diseases of the nervous system | Telephone-, videoconference- or web-based NPs assessment tools being remotely administered with or without supervision | PubMed, PsycInfo, Embase  and Scopus | 14 |

Legend: ACM (Association for Computing Machinery), CINAHL (Cumulative Index to Nursing and Allied Health Literature), SID (State Inpatient Database), ePRO (electronic patient-reported outcome), ASSIA (Applied Social Sciences Index & Abstracts), COPD (Chronic obstructive pulmonary disease), NHS (National Health Service), t-NPs (Transactional NPs), PRO (Patient reported outcomes)
