# Supplementary material for: The Current Status of Telemedicine Technology Use Across the World Health Organization European Region: An Overview of Systematic Reviews
Source: J Med Internet Res. 2022 Oct 27;24(10):e40877. doi: 10.2196/40877 (PMC9650581; doi:10.2196/40877)
Supplement: Multimedia Appendix 6 [file jmir_v24i10e40877_app6.docx]

**Table 2** - Summary of findings (SOF) table for main outcomes

|  |  | | **Certainty of the evidence (GRADE)** | | | | | |
| --- | --- | --- | --- | --- | --- | --- | --- | --- |
| **Outcome** | **Summary effect by chapter and the number of studies (*n*)** | | **Methodology Limitations*** | **Inconsistency**^†^ | **Indirectness^‡^** | **Imprecision^&^** | **Publication bias^\|\|^** | **Overall Quality** |
| Effects of telemedicine interventions in multiple medical conditions  (*n = 5*) | **Improve the delivery of care and positevely impacts cliical and diagnostic outcomes** | Chapter V – Mental, behavioral, or developmental disorders (*n = 6*) | Critical | Not serious | Serious indirectness | Not relevant | Potential publication bias detected | ⨁◯◯◯ VERY LOW^a^ |
|  |  | Chapter IX – Diseases of the circulatory system (*n = 5*) | Critical | Not serious | Serious indirectness | Not relevant | Potential publication bias detected | ⨁◯◯◯ VERY LOW^a^ |
|  |  | Chapter X – Diseases of the respiratory system (*n = 4*) | Critical | Not serious | Serious indirectness | Not relevant | Potential publication bias detected | ⨁◯◯◯ VERY LOW^a^ |
|  |  | Chapter XII – Diseases of skin and subcutaneous tissue (*n = 3*) | Critical | Not serious | Serious indirectness | Not relevant | Potential publication bias detected | ⨁◯◯◯ VERY LOW^a^ |
|  |  | Chapter II – Neoplasms (*n = 2*) | Critical | Not serious | Not serious | Not relevant | Not detected | ⨁⨁◯◯ LOW^b^ |
|  |  | Chapter VII – Diseases of the eye and adnexa (*n = 1*) | Critical | Not serious | Serious indirectness | Not relevant | Not detected | ⨁⨁◯◯ LOW^b^ |
|  |  | Chapter VI – Diseases of the nervous system (*n = 1*) | Critical | Not serious | Serious indirectness | Not relevant | Not detected | ⨁⨁◯◯ LOW^b^ |
|  |  | Chapter XVIII – Symptoms, signs, and abnormal clinical and laboratory findings, not elsewhere classified (*n = 1*) | Critical | Not serious | Serious indirectness | Not relevant | Not detected | ⨁⨁◯◯ LOW^b^ |
|  | **cost-effectiveness** | Reduction of health-related cost (*n = 9*) | Critical | Serious imprecision | Not serious | Relevant | Publication bias detected | ⨁⨁◯◯ LOW^c^ |
| **EXPLANATIONS**  a) Downgraded due to methodological limitations, indirectness, and publication bias among the included systematic reviews. For each domain, the overall judgement was based upon the respective included studies analyses; therefore, the final domain-specific judgment was established in accordance with most studies (> 50%).  b) Downgraded due to methodological limitations of the studies (most included reviews had an overall critically low methodological quality).  c) Downgraded due to methodological limitations of the studies (most included reviews had an overall critically low methodological quality), imprecision, inconsistency, and publication bias.  *Methodology limitations were essentially associated with the overall AMSTAR 2 rating.  †Inconsistency was judged by evaluating the consistency of the direction and primarily the difference in the magnitude of effects across studies (since statistical measures of heterogeneity are not available). For most of outcomes, we did not find differing results for each outcome across included studies. Therefore, we considered “not serious” risk for inconsistency. Nevertheless, for the “Cost-effectiveness” outcome, our search yielded widely differing estimates of effect, reason why we downgraded the certainty of the evidence.  ‡We downgraded the indirectness domain for most diseases and conditions because although the included systematic reviews met our eligibly criteria, they moderately addressed a particular version of the main review question in terms of population, comparator, and/or outcomes. For the “cost-effectiveness” outcome, we did not judge it serious as the evidence found and included was not likely to occur.  ^&^We downgraded imprecision if the findings suggest relevant differences among studies or with the number of studies assessing a particular outcome is very low.  \|\| We downgraded the publication bias domain if the body of literature appears to selectively evidence certain topic or trend from a specific outcome.  **GRADE Working Group grades of evidence** **High certainty:** The summary rating of included studies provides a very good indication of the likely effect. The likelihood that the effect will be substantially different is low. **Moderate certainty:** The summary rating of the included studies provides a good indication of the likely effect. The likelihood that the effect will be substantially different is moderate. **Low certainty:** The summary rating of the included studies provides some indication of the likely effect. The likelihood that the effect will be substantially different is high.  **Very low certainty:** The summary rating of the included studies does not provide a reliable indication of the likely effect. The likelihood that the effect will be substantially different is very high. | | | | | | | | |
