# Supplementary material for: The Current Status of Telemedicine Technology Use Across the World Health Organization European Region: An Overview of Systematic Reviews
Source: J Med Internet Res. 2022 Oct 27;24(10):e40877. doi: 10.2196/40877 (PMC9650581; doi:10.2196/40877)
Supplement: Multimedia Appendix 7 [file jmir_v24i10e40877_app7.docx]

**Table S1.** Main findings from included reviews.

| Review ID (reference) | Primary study design | | Number of patients | | Publication design | | Data type and collection | | Main results |
| --- | --- | --- | --- | --- | --- | --- | --- | --- | --- |
| **Chapter V—Mental and behavioral disorders** | | | | | | | | | |
| Elbaz et al [26] | Cross-sectional or longitudinal studies, cross-sectional survey, and collaborative action research | | 231 | | Systematic review without meta-analysis | | Data from psychological assessment transmitted via videoconferencing and telephones | | 1. Regarding cognitive and mental health outcomes, the studies showed discrepancies in the various assessment tests of individuals with cognitive impairment receiving both telephone and video calls compared with the telephone service only groups (some studies found a significant difference; others did not).  2. Individuals receiving additional telemedicine video calls were able to achieve higher cognitive assessment scores and quality of life ratings.  3. Older participants’ ability to access telemedicine consultations or services was still limited because of poor connection issues and dependence on a young caregiver to handle devices.  4. Only 1 study found that telemedicine calls between care homes provided some residents with renewed energy and self-purpose.  5. Furthermore, 1 study suggested that remote neuropsychological testing was a feasible endeavor. |
| Kingsdorf and Pancocha [35] | Single-subject experimental designs, randomized controlled trial, and group comparison | | 72 children and 95 parents | | Scoping review | | Data from behavioral intervention instruments | | 1. This review found a limited number of studies in this area. However, of the studies available, Iceland and Albania were the most targeted locations.  2. The participants were typically children with autism and their mothers.  3. The intervention strategies for training caregivers to implement procedures more often or more stringently often used components of behavioral skills training or coaching to increase their children’s communication responses.  4. A single-subject methodology was used most to assess increases in those measurable behaviors.  5. Videoconferencing was the most used telehealth component and had a high level of acceptability.  6. Overall, outcome data, when collected on children and caregiver behaviors, were positive. However, several barriers to, and opportunities for, future research existed. |
| Martin et al [38] | Qualitative studies, randomized experimental trials, and quantitative observational studies | | – | | Systematic review without meta-analysis | | Clinical outcomes, patient-level impacts, patient and health care professional satisfaction, and costs transmitted via any type of digital technology | | 1. In total, 7 (58%) of the 12 included studies reported improvements in young adults’ mental health symptoms. It was recognized that these changes correlated with the patients’ and clinicians’ motivation to use technologies.  2. The impact of network communication interventions on mental health is inconclusive.  3. Studies found that most of the children, adolescents, and parents in the studies were satisfied with networked communication technologies.  4. Adolescents (17/19, 89%) said they preferred the videoconferencing system to traveling for an assessment, and the same number said that they would use telepsychiatry again.  5. All parents “liked” the telepsychiatry assessment and would use the system again. |
| Simmonds-Buckley et al [46] | Randomized controlled trials | | 7075 | | Systematic review with meta-analysis | | Web-based or smartphone app intervention | | 1. All 6 web-based e-therapies had both clinical and academic personnel adding expertise during technological development, but the smartphone-based e-therapy had no clinical or academic personnel involved in its technological development phase.  2. The e-therapy treatments lasted between 10 and 70 days, comprising between 3 and 45 sessions lasting 10-60 minutes each.  3. The e-therapies were delivered for symptoms of depression, anxiety, panic, phobia or stress, or a combination of anxiety and depression symptoms.  4. Dropout rates ranged from 0% to 64%. The average e-therapy dropout rate was 31%, and the average dropout rate for controls was 17%.  5. There was a significant treatment effect in favor of greater depression reductions following e-therapy.  6. Meta-regression analyses found that variations in e-therapy treatment effects were not explained by gender, age, number of sessions, or study quality at posttreatment period or follow-up.  7. There was a small to moderate, significant treatment effect in favor of greater anxiety reductions following e-therapy. |
| **Chapter VI—Diseases of the nervous system** | | | | | | | | | |
| Maresca et al [37] | Randomized control trials, comparative studies, and systematic review with and without meta-analyses | | – | | Scoping review | | Motor and cognitive improvement outcomes transmitted via remote consultation systems | | 1. This review showed that telerehabilitation is a promising health care tool as it guaranteed the continuity of care over time (after discharge) and in space (from hospital to a patient’s home), especially in patients with stroke.  2. Furthermore, it allowed the frequency and intensity of rehabilitation programs to be increased, individualized rehabilitation treatment to be provided in a comfortable and familiar environment for patients, patients’ needs and progress to be monitored and assessed, patient motivation to be stimulated, better patient satisfaction to be achieved, patient outcomes to be verified, and service costs to be potentially reduced. |
| Ohannessian et al [43] | Medical and economic modeling, medical and economic prospective cost-benefit study, observational analytic retrospective case-control studies, observational studies, and quasi-randomized and randomized clinical trials | | – | | Systematic review without meta-analysis | | Clinical outcomes transmitted via remote consultation systems | | 1. Implementation of regional acute telestroke activities was proven to be feasible and safe with no significant difference between care with or without telemedicine.  2. The implementation of acute telestroke was shown to be effective in improving regional access to acute stroke care.  3. Limited support and funding for public health policy evaluation and interventional research at national and regional health levels may have influenced the lack of published results in France. |
| Zanin et al [53] | Cohort based study | | – | | Systematic review without meta-analysis | | Multidomain screening tools for cognition, behavior, mood/anxiety, and quality of life were the most represented ones. Findings regarding validity, reliability, sensitivity, specificity, and clinical usability were reported for cognitive screenings (over the telephone), videoconference-based Mini-Mental State Examination, and Telephone Interview for Cognitive Status | | 1. The review suggests the validity, reliability, and usability of telephone-, videoconference-, and web-based short screening instruments for cognitive and behavioral impairment in the Italian population.  2. Overall, remotely administered instruments showed moderate to high internal consistency and both construct and criterion/ecological validity with respect to both standardized and ad hoc semistructured tools. |
| **Chapter VII—Diseases of the eye and adnexa** | | | | | | | | | |
| Labiris et al [36] | Descriptive and comparative studies (prospective and retrospective studies) | | – | | Systematic review without meta-analysis | | Ophthalmic examination via videoconferencing platforms | | 1. Most studies (35/44, 80%) indicated the method for care provision in ophthalmology patients, which delivered outcomes comparable with traditional examination methods.  2. All published studies (27/44, 61%) indicated that telemedicine provided comparable outcomes when compared with conventional examination methods in hospital settings. |
| **Chapter IX—Diseases of the circulatory system** | | | | | | | | | |
| Brunetti et al [22] | Longitudinal studies | | 7552 | | Systematic review with meta-analysis | | Electrocardiogram traces transmitted to a medical center | | 1. In the meta-analysis study, the overall relative reduction in time to treatment with prehospital triage and telemedicine was 40%, and in random effect analysis, it was 38%.  2. Absolute time reduction was significantly correlated to time to treatment in control groups, while relative time reduction was independent.  3. A nonsignificant trend toward shorter absolute time to treatment was observed over the years and in studies enrolling a larger number of patients.  4. Funnel plot inspection revealed a publication bias, with a larger relative time reduction in smaller studies. |
| Carbo et al [23] | Randomized clinical trials | | 1206 | | Systematic review without meta-analysis | | Mostly mobile devices | | 1. Mobile devices were most commonly used to transmit data as part of the monitoring systems.  2. Adherence to data transmission during the studies’ follow-up period was >80% of the time in most cases.  3. Medical personnel not empowered to make clinical decisions often routed out-of-limit alerts to clinicians who evaluated the data and took further action.  4. Several studies provided heart failure education for all enrolled patients.  5. Of the 4 studies that examined heart failure–related admissions, 2 (50%) found a reduction of 44% and 48%, respectively, in the mHealth^b^ group. Out of the 3 studies, 1 (33%) found a 65% reduction in the number of emergency department visits.  6. A total of 3 studies found significant improvement between 14% and 17% in left ventricular ejection fraction in the mHealth group from baseline to the end of the study.  7. A total of 2 studies observed a reduction of 30%-57% in the intervention group compared with baseline levels of brain natriuretic peptide. |
| Farabi et al [27] | Modeling studies, cost-utility analyses, and cost-effectiveness assessments | | 24,099 (including hypothetical patients) | | Systematic review without meta-analysis | | Economic data | | 1. Telemedicine improved clinical outcomes and resulted in considerable cost savings.  2. Using telemedicine concurrent with usual care for service delivery was more cost-effective.  3. Studies used different analysis models (Markov model, Cox regression model, and linear mixed models).  4. All studies included direct medical costs in their analysis, but they did not include nonmedical costs and direct nonmedical costs.  5. Both screening and identifying people with high blood pressure using telemedicine were cost-effective, reduced the mortality rate, and led to an increase in patients’ quality of life.  6. Remote monitoring of implantable devices not only improved patients’ quality of life and reduced the years of life lost but also had many secondary consequences (reduced hospitalization, unnecessary visits, and patients’ absence from work). |
| Ohannessian et al [43] | Systematic reviews, randomized controlled trials, reports, and cohort studies | | – | | Systematic review without meta-analysis | | Therapeutic cardiac implantable electronic device data transmitted via remote monitoring systems | | 1. There was limited clinical trial information on the effectiveness of RMSs^c^ for pacemakers. However, for RMSs for implantable cardioverter-defibrillator devices, multiple cohort studies and 2 large multicenter randomized control trials demonstrated feasibility and significant reductions in in-office clinic follow-ups with RMSs in the first year after implantation.  2. The detection rates of clinically significant events (and asymptomatic events) were higher, and the time to clinical decision for these events was significantly shorter in the remote follow-up groups than in the in-office follow-up groups.  3. However, the earlier detection of clinical events in the remote follow-up groups was not associated with lower morbidity or mortality rates in the 1-year follow-up.  4. The substitution of almost all the first-year in-office clinic follow-ups with RMS was also not associated with increased health care use such as emergency department visits or hospitalizations.  5. Patients’ acceptance of and satisfaction with RM^d^ were reported to be high, but the impact of RM on patients’ health-related quality of life, particularly psychological aspects, was not evaluated thoroughly.  6. Patients who are not technologically competent or having hearing or other physical/mental impairments were identified as potentially disadvantaged with RM.  7. Cohort studies consistently identified subgroups of patients who preferred in-office follow-up.  8. The evaluation of costs and workflow impact on the health care system were evaluated in European or American clinical settings but only in a limited way. |
| **Chapter X—Diseases of the respiratory system** | | | | | | | | | |
| Cruz et al [25] | Randomized clinical trials, uncontrolled before-and-after study, and nonrandomized clinical studies | | 391 | | Systematic review without meta-analysis | | Performed manually using touchscreen monitors. Data were transmitted daily in almost all studies | | 1. Two important dropout reasons were patients’ financial limitations on recharging the device batteries every 2 days (devices had short-lived duration) and the fact that the device was too difficult to carry around.  2. Additional dropout reasons were related to usability problems, readability difficulties, poor fine motor control issues, and technical problems with the system or telephone line.  3. Overall, patients found the technology easy to learn or use and useful.  4. The system improved self-management of their health condition, as they had a better understanding of their disease, its symptoms, and the way to control them; they were more involved in their health care and recognized signs of exacerbation earlier.  5. Systems also improved the care received from health professionals.  6. Patients felt a sense of security and reassurance when using the system because they knew their health conditions were being monitored by real individuals. |
| Gaveikaite et al [28] | Systematic reviews | | – | | Other | | Clinical and epidemiological data via remote monitoring devices | | 1. The use of telemedicine reduced the number of hospital admissions and the number of visits to the emergency department and significantly decreased the exacerbation rate.  2. The use of telemedicine had a potentially significant improvement effect on the Saint George Respiratory Questionnaire. |
| Hallensleben et al [30] | – | | – | | Scoping review | | Clinical and epidemiological data; mostly mobile devices | | 1. A total of 47 eHealth apps were found (divided into care programs, patient care, and informative eHealth platforms).  2. Of the 13 studies, 5 (38%) included eHealth care programs and patient platforms that showed positive results in terms of improving quality of life or reducing hospital admissions.  3. Most care programs were still part of ongoing research, mainly situated in secondary health care (essentially involving patients with severe conditions, under pulmonologist care).  4. Scientific evidence for the effectiveness of various self-care and informative websites was limited or completely absent. The uptake of certain information depends on accessibility, advertising, and recommendation by individual care providers.  5. A total of 15 apps found focused on COPD^e^ care, medication adherence, smoking cessation, and information about disease-related topics.  6. A total of 6 apps were found to be effective and free, and most apps were available to all patients on both iOS and Android systems. |
| Udsen et al [50] | Randomized controlled trials | | – | | Systematic review without meta-analysis | | – | | 1. The review demonstrated potential cost savings from telehealth for patients with COPD.  2. The cost savings accrued mainly to hospitals and the health care sector according to 6 studies. However, caution was advised about the use of telehealth in routine clinical practice because the large-scale effect of telehealth is unknown, as the quality of economic evidence is poor.  3. The clinical effectiveness of the large-scale implementation of telehealth with follow-up exceeding 12 months has not yet been demonstrated. |
| **Chapter XII—Diseases of the skin and subcutaneous tissue** | | | | | | | | | |
| Hrynyschyn et al [32] | Randomized clinical trials and controlled clinical trials | | 2711 | | Systematic review without meta-analysis | | Clinical and epidemiological data as well as photos and videos transmitted via mobile devices/mostly mobile devices | | 1. Studies examined the use of telemedicine to treat diabetic foot ulcers and general chronic wounds.  2. Most studies (5/6, 83%) saved captured images on a laptop or in a web-based database.  3. Overall, 50% (3/6) of the included studies found a positive effect of teleconsultation on wound healing. Only 1 study reported a significantly shortened wound healing time in the telemedicine group compared with the control group. In addition, a higher rate of wound healing was observed in another study.  4. In total, 2 studies reported no significant difference among interventions in terms of mortality rate.  5. A study found no significant differences between the intervention and control groups regarding amputation rate. Smith-Strøm et al reported a significantly lower number of amputations in the telemedicine group.  6. The use of digital photos and videos was found to be suitable for making a diagnosis or formulating therapy recommendations. |
| Singh et al [47] | Nonrandomized, randomized, quasi-experimental, and feasibility studies | | Approximately 380 | | Systematic review without meta-analysis | | Clinical/economic, diagnostic, diagnostic/economic, and behavioral | | 1. Telehealth was used as a stable mode of contact between all stakeholders to ensure continuity of care in chronic disease management in a cost-effective manner.  2. Some studies (2/11, 18%) reported good diabetic foot ulcer healing rate in rural areas using telehealth.  3. Telehealth assessment of diabetic foot ulcer using 3D images yielded reproducible results with higher accuracy compared with traditional face-to-face manual foot ulcer examination. |
| Trettel et al [49] | General evaluation (not specified), patient management referral/triage, diagnosis/consultation of patients in remote/peripheral locations, monitoring/consultation in nursing-home/home-care settings and emergency diagnosis | | – | | Systematic review without meta-analysis | | Several skin diseases and conditions transmitted via remote consultation systems | | 1. Patient satisfaction was usually high, and the use of teledermatology was part of daily routine.  2. Telemedical consultation of skin cancer was proven equivalent to classical face-to-face examination. This was important because, given its high incidence rates across the world, an urgent assessment was usually necessary.  3. Telemedical wound management showed similar results, and teledermatology might become an essential tool for medical institutions such as nursing homes or for in-home care. |
| **Chapter XVIII—Symptoms, signs, and abnormal clinical and laboratory findings, not elsewhere classified** | | | | | | | | | |
| Cordes et al [24] | Cross-over and prospective comparative studies and a quasi-randomized clinical trial | | 42 | | Scoping review | | Electronic medical records | | 1. In the Italian study, both face-to-face and screen-to-screen therapy showed significant clinical improvement in speech performance among treated patients. However, no difference was observed between the 2 interventions.  2. In the German study, a significant before-and-after intervention effect could only be verified among patients treated using the face-to-face methodology.  3. In the British study, both interventions improved clinical performance compared with the control group. In an extended assessment, the screen-to-screen therapy practice group achieved a significantly higher value of the naming performance for trained and untrained words than the other groups. |
| **Chapter XXI - Factors influencing health status and contact with health services** | | | | | | | | | |
| Willard et al [52] | – | – | | Scoping review | | Characteristics and features of included platforms. | | The results have provided insight into how these platforms should be classified, what is the nature and the capabilities of these online platforms and how many online platforms exist according to the potential users concerned (older adults, professionals, informal caregivers, and municipalities). | |
| **Multifocal study** | | | | | | | | | |
| Allner et al [21] | Longitudinal studies | | – | | Systematic review without meta-analysis | | – | | 1. Included studies were mostly related to telemedicine in stroke care and management.  2. Telemedicine was used in rural areas as a structured intervention guide for stroke treatment, which was essentially available to physicians and neurology specialists.  3. Furthermore, some studies (7/16, 44%) highlighted the usability of telemedicine for diabetes management, home monitoring of locked-in patients with multiple morbidities, and medical emergency purposes.  4. Some studies (6/16, 38%) emphasized concerns about patient and staff safety and technology reliability, and some (7/16, 44%) also considered sociocultural, ethical, and legal aspects. |
| Glinkowski et al [29] | – | | – | | Systematic review without meta-analysis | | – | | 1. Telemedicine and eHealth were mostly used only in selected clinical disciplines such as cardiology (21/130, 16%), family medicine (20/130, 15%), and pathology (14/130, 11%).  2. Among studies published for medicine-oriented conferences, other medical specialties including orthopedics, rehabilitation, radiology, and psychiatry had a higher annual volume of publication frequency compared with standard article publication. |
| Hartasanchez et al [31] | Observational, feasibility and clinical pilot studies, and randomized clinical trials | | 412 (including simulated patients) | | Systematic review without meta-analysis | | Patients’ demographic features and synchronous and asynchronous data use of any digital technology | | 1. The literature suggested that, in addition to the need for improved access and patient training, there was still a need to integrate patients and clinicians in the use of technology and internet-based care within clinical workflows.  2. Participants’ willingness to use and adopt a technology was key to its successful use, as was offering a choice in terms of selecting which remote approach would be used.  3. Video consultations improved patient and clinician satisfaction with the encounter, particularly when they perceived that more time was spent, and more attention was paid.  4. The digital tool’s main purpose was to facilitate communication, promote shared care planning, and reduce treatment burden.  5. The asynchronous approach might reduce the burden placed on patients in terms of collecting and organizing the information used to make visits more efficient and conversations with the clinician more productive. |
| Karamanidou et al [33] | Study protocols, technical tools/solutions, pilot/feasibility/acceptability studies, and an evaluation study | | – | | Systematic review without meta-analysis | | ePRO^f^ (defined as measurements electronically reported directly by the patient about their health status, without amendment or interpretation by a physician or anyone else) | | 1. The proposed digital health interventions were rated positively by end users with respect to usability, user acceptance, and satisfaction, with few concerns.  2. Findings demonstrated that ePRO interventions could have a significant positive impact on health outcomes.  3. Patients with cancer are generally in favor of ePRO-based interventions, and ePRO interventions could contribute to improved health outcomes, such as an improvement in physical activity; a reduction in anxiety and drowsiness; and lower levels of fatigue, nausea, insomnia, and pain intensity as well as a significant improvement in emotional and social functioning. |
| Kierkegaard [34] | – | | – | | Other | | Telemedicine project mapping | | 1. The results of this study suggested that a growing number of telemedicine initiatives were currently in operation across Denmark but that considerable variations existed in terms of regional efforts.  2. Despite such differences, some similarities were also found; all regions identified clinical/professional benefits as a primary motivator.  3. Financial benefit was the primary driver for urban areas, implying that high population density areas were motivated by cost-benefit.  4. Low population density areas sought benefits for patients/citizens and organizations.  5. The findings implied that population density played a role in the reasoning behind an organization’s use of telemedicine. |
| McFarland et al [39] | Qualitative papers, single-site cohort, multisite cluster trials, and randomized clinical trials | | 2611 | | Systematic review with meta-analysis | | Quality of life–related data and health outcomes | | 1. Telehealth was not statistically significantly different from standard home care for quality of life, psychological well-being, physical function, anxiety, depression, disease-specific outcomes, or bed days of care at 3, 6, 9, and 12 months.  2. The differences in QALYs^g^ among the intervention arms were negligible at 0.549 and 0.564 for standard home care and telehealth, respectively.  3. Qualitative findings showed that patients found telehealth beneficial for providing peace of mind and increased access to health care, improving ownership, and promoting self-management. |
| Mold et al [40] | Surveys, quasi-experimental and randomized clinical trials, cohorts, cross-sectional designs, case studies, interviews, focus groups, and mixed methods studies | | – | | Systematic review without meta-analysis | | Clinical and behavioral outcomes as well as organizational issues | | 1. There were disparities in uptake and use toward more use by younger, employed adults.  2. Patients reported satisfaction with services and improved self-care, communication, and engagement with clinicians.  3. Evidence for acceptability and ease of use was strong, especially among patients with long-term conditions and those located in remote regions. However, patients were concerned about the privacy and security of their data.  4. A range of studies found specific advantages to using e-consultations including improved access to care, both in the delivery of care outside of standard working hours and to remote areas, time savings, and cost savings, including lost wages.  5. Joint e-consultations among GPs^h^, specialists, and patients resulted in significantly higher levels of patient satisfaction.  6. Greater e-consultation use was associated with winter months, especially for patients (and families) using video consultations in rural and remote communities.  7. Survey evidence suggested that telemedicine was as good as or even better than face-to-face consultation regarding the explanation of care to patients.  8. Email consultations were also shown to be clinically feasible in terms of diagnostic accuracy.  9. Several studies reported clinicians’ reluctance to use email with their patients because of increased workload concerns.  10. Costs of clinicians’ time to support joint consultations were unlikely to be offset against subsequent savings to health care services in the short term. |
| Nielsen et al [41] | Various quantitative and qualitative studies | | 14,712 | | Scoping review | | PRO^i^ data. Specifically, data on nonparticipant and dropout rates, reasons for nonparticipation or dropout and the characteristics of nonparticipants and dropouts. Also reviewed were study population, intervention, type of PROs, intervention duration, and use of reminders | | 1. Most studies reported problems with both nonparticipation and dropout.  2. In general, patients unwilling to give informed consent to participate in clinical studies were younger and more likely to be women.  3. Patients’ reasons for declining participation and dropping out during studies were manifold and included the ability to use PRO, emotional distress, variable levels of engagement (owing to the lack of symptoms, time pressures, did not see the benefit, or not interested), technical issues, usability, and data security and trust. |
| O’Cathail et al [42] | Pilots, audits, service reports, case series/reports, and randomized clinical trials | | – | | Scoping review | | Technical feasibility, user acceptability, clinical effectiveness, economic assessment, and logistical and operational considerations transmitted via videoconferencing | | 1. Overall, the studies were heterogeneous in design, in the specialty assessed, and reported outcome measures.  2. The technology used for teleconsultations changed over time with earlier studies using bespoke, often expensive, solutions.  3. Later studies transitioned to web-based commercial solutions such as Skype.  4. Five outcome measures were assessed: technical feasibility, user satisfaction, clinical effectiveness, cost, and logistical and operational considerations.  5. Owing to the changing nature of technology over time, there were differing technical issues across the studies.  6. Generally, teleconsultations were acceptable to patients, but this was less consistent among health care professionals. However, among both groups, face-to-face consultations were still seen as the gold standard.  7. A wide range of clinical scenarios found teleconsultations to be clinically useful but potentially limited to more straightforward clinical interactions.  8. Owing to the wide array of study types and changes in technology over time, it was difficult to draw definitive conclusions on the cost involved. However, cost savings for health care providers have been demonstrated by goal-directed implementation of teleconsultations. |
| Raja et al [45] | Quantitative and qualitative studies | | – | | Scoping review | | – | | 1. Information from older people about their needs in telehealth interventions was important for successful implementation.  2. There was likely to be a range of barriers needing to be overcome to get older people to use and benefit from telehealth and technological shifts. Functional limitations due to age were mentioned several times, including difficulties remembering instructions, and these were seen as important barriers. |
| Verma and Kerrison [51] | Quantitative study, qualitative study, mixed methods study, and multimethods study | | – | | Other | | Qualitative and quantitative data regarding the experiences of PCPs^j^ and patients | | 1. Patients and PCPs were generally satisfied with remote consultations and believed them to be preferable for specific appointments, such as follow-up of a previous face-to-face appointment.  2. The results suggested specific situations in which remote consultations could be used in a post–COVID-19 pandemic world. However, while remote consultations were more convenient and protected patients and staff against COVID-19 infections, they resulted in a loss of valuable nonverbal communication and were not accessible to all.  3. Several situations where remote consultations should not be used were noted, such as when a patient was presenting to a GP for the first time. |
| Tokgoz and Dockweiler [48] | Randomized clinical trials | | 1418 | | Systematic review without meta-analysis | | Web-based or smartphone app intervention | | 1. Although the power of certainty was still low, studies suggested positive effects in certain parameters of functioning, including therapy-induced menopausal symptoms, fatigue, and sleep functions.  2. Included studies also found improvements in cognitive functions following telemedicine interventions.  3. The results associated with the effect of telemedicine on quality of life outcomes were discrepant and inconclusive (no specific trend was observed). |

^a^ –: Data is not available.

^b^mHealth: mobile health.

^c^RMS: remote monitoring system.

^d^RM: remote monitoring.

^e^COPD: chronic obstructive pulmonary disease.

^f^ePRO: electronic patient-reported outcome.

^g^QALY: quality-adjusted life year.

^h^GP: general practitioner.

^i^PRO: patient-reported outcome.

^j^PCP: primary care physician.
