# Supplementary material for: The Current Status of Telemedicine Technology Use Across the World Health Organization European Region: An Overview of Systematic Reviews
Source: J Med Internet Res. 2022 Oct 27;24(10):e40877. doi: 10.2196/40877 (PMC9650581; doi:10.2196/40877)
Supplement: Multimedia Appendix 8 [file jmir_v24i10e40877_app8.docx]

**Table 3.** List of barriers, facilitators, limitations and current challenges

| **Review ID, Publication Year** | **Listed Barriers** | | **Highlighted Facilitators** | | **Main Limitations Pinpointed** | | **Current Challenges** | |  |
| --- | --- | --- | --- | --- | --- | --- | --- | --- | --- |
| **Chapter V - Mental and behavioural disorders** | | | | | | | | | |
| [26] | 1. Lack of access to a helpful caregiver. 2. Individuals with different grades of dementia, who were not used to technologies. 3. Connectedness. 4. Organisational issues creating barriers to long-term implementation. 5. Situational loneliness needing to be overcome. | | None | | 1. Few quantitative mental health assessments were included. 2. Most telemedicine assessments or interventions were video conference-based. 3. Studies were primarily selected after the pandemic. | | 1. Future studies should examine the evolution and changes that telemedicine has undergone since the arrival of COVID-19 and examine individuals’ experiences longitudinally. | |  |
| [35] | 1. High-speed Internet access was a challenge for participants, therefore, technology usage was kept simple. 2. Attrition was a pervasive barrier across all studies. 3. Most of the studies noted that there was collaboration with professionals based in North America to support their work; this highlights Europe’s barrier of access to high-quality information and services grounded in behavioural science and its telehealth modality. | | 1. Should consider creating incentive programmes to sustain families’ participation in telehealth services. 2. To strengthen male involvement in child-focused telehealth services, fathers should be treated as co-parents, increasing fathers’ knowledge and awareness of the intervention. 3. Future work on behavioural interventions delivered to families via telehealth should continue to take into consideration the bandwidth needed to access certain technological modalities, alternative ways to access information in the case of Internet malfunctions, and even technology confidence prior to intervention. | | 1. Only one study used the gold standard of research design: a randomised controlled trial. 2. All the studies had relatively small sample sizes which made the generalisability of the research questionable. 3. Most included articles were peer-reviewed, however, one thesis was included. 4. Only studies in English were targeted. 5. Narrow date range used for the search. 6. Studies all specifically discussed large losses of participants during their research. | | 1. Study quality needs to be more rigorous to build a solid foundation of behavioural telehealth in Europe. 2. Future work should consider increasing the number of study participants, increasing data collection methods to include more outcome measures focusing on direct data collection, extending methods to provide data validation, and increasing assessments of social validity to see the impact of the intervention on not just perceived effectiveness, but also on subsequent applications of skills after training. 3. The need to form partnerships with study participants and the community by including them in planning to support intervention buy-in, and recruiting local interventionists to support the sustainability of the outcomes. | |  |
| [38] | Identified concerns regarding confidentiality resulting from lack of computer privacy/security. | | The provision of frequent and multi-modal communication (integration of verbal, written, and other non-verbal methods) between practitioner and patient seemed important for recovery or symptom reduction in the therapeutic relationships observed. Successful interventions particularly incorporated written communication. | | The research area was rapidly changing; terminology and technologies had not become established and used routinely, thus making comparisons difficult. A relatively small number of technologies was identified despite using a broad search strategy. The review was limited to publications in English. 2. There was a lack of high quality qualitative evidence, while response rates in experimental and observational quantitative studies were weak. | | Further research is needed to explore patient and clinician acceptability and satisfaction issues and to determine whether technologies are reliable, helpful and easy to use. Future studies should clearly document the training needs of patients and providers to master the equipment, as well as the implications of implementing technology in mental care. These new technologies involve changes and adaptations in identified roles and mutual expectations, which are context-dependent. | |  |
| [46] | The acceptability of e-therapies could be called into question because of the higher dropout rates compared to controls. | |  | | 1. Although the included studies were restricted to high-quality randomised controlled trials, the GRADE approach highlighted issues with inconsistency across results, treatment comparisons, and some imprecision resulting in meta-analytic comparisons of moderate-to-low quality. 2. There were limitations concerning the generalisability of the findings. This review was limited to the treatment of depression, anxiety and stress with e-therapies and therefore could not comment on applicability to other clinical presentations. | | 1. Unique and standardised identification of e-therapies among e-therapy developers and researchers. 2. Trials of e-therapies should also be reported according to the CONSORT-EHEALTH checklist. 3. Health economic evaluations that are embedded in clinical trials need to be increased. 4. Treatment adherence (i.e., time spent and number of eHealth modules completed by participants) needs to be more consistently reported. 5. The role of moderating factors of treatment outcome in e-therapies needs to be better researched, particularly the role of variables such as blended vs pure e-therapy approaches, time spent on the app, and the theoretical approach. | |  |
| **Chapter VI - Diseases of the nervous system** | | | | | | | | | |
| [37] | The difficulty of creating software suitable for a remote therapeutic setting. Flexible devices adaptable to different types of shortcomings, and broad connectivity were needed to better reach users at home. | | 1. Considering the growing burden of care and the need to provide adequate and continuous services to chronic patients, telerehabilitation was becoming an interesting and promising model of care. | | 1. The samples in these studies were small, thus rendering the results not especially relevant for a substantial economic analysis. 2. All the research on telerehabilitation considered in this review highlighted the need to standardise the procedures, purposes and targets characterising this therapeutic modality. | | Improving the quality of the research in this area, especially for gathering more information about cost-effectiveness. | |  |
| [43] | The lack of a dedicated countrywide funding model from the health authorities.  The lack of a clearly defined funding model might hinder a full national expansion of sustainable acute telestroke models in France. | | None | | 1. Published results were not available from all regions of France, and reported results were limited in terms of the ability to conclude on safety and effectiveness at a national level.  2. A standardised research methodology for acute telestroke evaluation was not used across all regions of France, which may have restricted the interregional and international comparability of the results.  3. The methodologies used by the different studies precluded specific identification of telestroke as a direct causal factor for improved access to acute stroke care.  4. Definitions of study indicators were also not always provided, limiting interpretation of the results.  5. No conclusions could be drawn from the various medical and economic studies concerning the efficiency of regional acute telestroke activities.  6. There was a lack of national- or regional-based digital stroke registries in France used in evaluating new stroke care models that included acute telestroke. | | The development of thrombectomy in France, supported by published national guidelines, and the potential development of mobile stroke units, may eventually impact the existing territorial acute telestroke organisation. Further development and evaluation of regional acute telestroke activities should be considered. | |  |
| [53] | Both the self-report modality adopted by remotely assessed clinical scales and the complexity of the target constructs made any comparison with the reported evidence on the psychometric quality of cognitive tests challenging. | | 1. Convenience was the primary reported reason for patients choosing an e-consultation. 2. Timeliness of responses was important to patients using email, which was associated with satisfaction. | | 1. Scarce diffusion of web-based instruments. 2. A lack of studies on this topic. | | 1. The need for scientific, evidence-based recognition of t-NPs practice within current national guidelines. 2. Further studies should be carried out by researchers in order to examine feasibility and statistics of domain-specific t-NPs tools. 3. Future explorations might also clarify, within the country context, the relevance of t-NPs to clinical practice beyond the COVID-19 pandemic. | |  |
| **Chapter VII - Diseases of the eye and adnexa** | | | | | | | | | |
| [36] | 1. Low motivation for using telemedicine among professionals. | | 1. Cost reduction for health services but also for patients. 2. Reduction in the number of visits. 3. Patient satisfaction. 4. Clinical staff’s predisposition to use telemedicine. | | 1. The huge diversity of screening/diagnostic tools used, mainly cameras for capturing images, made the assessment of these devices difficult. 2. The lack of protocols to assess evaluation rates, gradeability of photographs, quality of grading and follow-up of screen-positive individuals. 3. Photographers and graders should be continuously educated and certified. | | 1. Improving the quality of image capturing in those interventions based on image analysis. 2. Generating cooperation among expert teams should play a pivotal role in the provision of telemedical care with comparable quality to that in conventional clinical settings. | |  |
| **Chapter IX - Diseases of the circulatory system** | | | | | | | | | |
| [22] | None | | None | | Preliminary data from non-randomised studies. A publication bias was found with smaller studies associated with higher relative time reductions that were not significant, however, in multi-variable analysis. | | None | |  |
| [23] | 1. An elevated cost of telemonitoring system implementation remained. 2. There was still a need to move from obsolete telemonitoring technologies towards mHealth devices (including mobile phones). | | None | | High heterogeneity of the studies given the wide range of mHealth interventions. | | Future studies should focus on quality measures in order to gather substantial evidence and determine the effectiveness and cost-effectiveness of mHealth. | |  |
| [27] | High cost of telemedicine technologies, as well as difficulties in implementation and follow-up over a longer time period. | | None | | 1. Heterogeneity of the evaluation methods and tools used as telemedicine in the final study, leading to challenging inter-study comparability. 2. Only studies in the English and Persian were included. | | The need for further studies in non-developed countries. | |  |
| [44] | The broader issues surrounding infrastructure, impacts on existing clinical care systems, and regulatory concerns needed to be considered for the implementation of Internet-based remote monitoring systems in jurisdictions involving different clinical practices. | | None | | The economic analysis was limited in that it only examined the impact on physician costs of providing remote monitoring of cardiac implantable electronic devices. Neither hospital nor clinic costs were examined, and were therefore unavailable. | | Insufficient information to evaluate the overall impact on the healthcare system. | |  |
| **Chapter X - Diseases of the respiratory system** | | | | | | | | | |
| [25] | Usability, financial and technical problems. The main barriers to implementation were described in the main results section. | | None | | 1. The study was restricted to studies published in English, Spanish and Portuguese. 2. The inclusion of studies not restricted to chronic obstructive pulmonary disease (mixed population). 3. Patients’ satisfaction was explored regardless of clinical outcomes and healthcare utilisation. | | 1. The need for more training sessions on how patients can properly use the implemented systems. 2. Before implementation of telemonitoring technologies, an assessment of patients’ needs, characteristics and acceptance of a telemonitoring app should be considered. 3. Studies should consider the inclusion of easy-to-use technologies for patients. 4. Frequency of data collection and transmission should be flexible to improve adherence to teleinterventions. | |  |
| [28] | Patient recruitment barriers and subsequent low rates of patient participation regarding studies using telehealth apps. | | None | | Overall, the results were brief, shallow and mostly qualitative. 2. Results only give a general view from a European perspective. | | 1. Future studies must include a cost-analysis assessment in their reporting to provide financial insights related to the implementation of telehealth. 2. An extensive list of telehealth alternatives and approaches should be considered and used in future research. | |  |
| [30] | None | | None | | 1. Inherent study design (scoping review). 2. Search was conducted by one author only. 3. Several eHealth app owners did not display data for numerical evaluation and processing. 4. Broad variety of included eHealth apps and difficulty in comparing them on the basis of the assessment criteria. | | 1. The need to investigate the long-term effects of eHealth apps for chronic obstructive pulmonary disease patients, in addition to the short-term effects. 2. The effectiveness of eHealth interventions should be studied to find out which are the most effective. 3. International collaboration is also required, either at the primary or secondary care level. | |  |
| [50] | N/A | | N/A | |  | | First, cost-effectiveness analyses are needed, which can relate incremental costs to a combined measure of mortality and morbidity (quality-adjusted life years, QALYs). Second, studies with broad analytical perspectives are needed. These studies may confirm or dismiss the risk that the introduction of telehealth involves, such as passing additional costs on to others responsible for caring for COPD patients. Third, studies with longer follow-up times are needed. The longest post-randomisation follow-up was 12 months. Since COPD is a chronic disease, we could reasonably expect that costs and outcomes will accrue later. Finally, cost studies with larger sample sizes and more detailed characteristics of the included patients are needed to allow for cost-of-illness studies and subgroup analysis. | |  |
| **Chapter XII – Diseases of the skin and subcutaneous tissue** | | | | | | | | | |
| [32] |  | | None | | 1. Timespan, publication design, and language limitations. 2. The literature search was limited to two databases. 3. Publication bias due to selective publication of patients with chronic wounds. 4. During the execution of trials in some studies, there were deviations from the intended medical treatment due to patient decompensation. | | 1. The need to understand and evaluate the health-related and economic implications of teleconsultation. 2. Innovative projects are crucial for early scientific development. 3. The real-time system should be critically analysed as it does not represent a reliable app in clinical practice. | |  |
| [47] |  | | N/A | | 1. No study investigated telehealth intervention against all clinical outcomes assessed, making it difficult to assess each individual intervention holistically with respect to all variables. 2. No quality assessment protocol was employed in the analysis of each individual study. 3. A more comprehensive search strategy including other databases might have provided additional relevant studies. | | 1. Future research should compare agreement between patient-based assessments and professional assessments. 2. Favourable results in this regard would add to the cost-effectiveness of telehealth and encourage patient autonomy.  3. Further studies should be aimed at establishing reproducible methods to diagnose important early signs of diabetic foot ulcers. 4. Further studies are required to develop efficient data transfer protocols and establish how these can be applied to, and implemented in, telehealth systems. | |  |
| [49] | Physicians’ confidence in clinical consultations remained higher, possibly to avoid misdiagnosis and legal issues, or because there was little information on clinical outcomes and long-term patient management. | | Financial and legal frameworks for teledermatology needed to be created to establish telemedical consultation as a permanent tool in daily practice, especially in rural areas.  Guidelines should be mandatory to avoid patient mistrust in the new technique. | | Publication bias (i.e., studies showing negative effects or inferiority of teledermatology might not have been published). | | 1. Future research should consider quality of life as an important outcome variable in healthcare. 2. Further studies that include more patients from peripheral locations, and especially from nursing homes, who are in serious need of professional medical assistance, should be conducted. 3. Further systematic studies, especially from countries with a geographically challenging healthcare situation, are needed to assess the value of teledermatology under such circumstances. 4. Teledermatological online apps need to be investigated with regard to medical safety. | |  |
| **Chapter XVIII - Symptoms, signs and abnormal clinical and laboratory findings, not elsewhere classified** | | | | | | | | | |
| [24] | | 1. Limitation regarding accessibility to electronic devices, including iPads, computers and laptops. 2. Internet connection might be a significant issue for some users. 3. Older adults may be affected by inexperience in dealing with technologies. 4. Programmers should be aware that planned apps ought to be user-friendly. 5. There is an additional limitation associated with individuals with visual and hearing impairments. | | None | | 1. Naming performance analysis was only conducted independently from other language modalities (including writing, reading, and language comprehension). 2. Selection and participant bias (mostly enrolment of older individuals). 3. Small samples in included studies. | | 1. Indispensable tactile stimuli should be transmitted from speech therapy to screen-to-screen therapy mode. 2. Digitalisation requires new tele-based methods for speech teletherapy in the sense of the Digital Healthcare Act. |  |
| **Chapter XXI - Factors influencing health status and contact with health services** | | | | | | | | | |
| [52] | | 1. The difficulty of discovering what the focus of a platform is and what services it has available, especially on the part of older adults. | | A system integrator or an open-source platform has the potential to interconnect with a variety of functionalities and systems, reducing the number of separate platforms and applications for older adults by integrating them within one single platform. | | 1. Based solely on grey-literature reports. | | It is challenging to successfully exploit these online care platforms, as they have not yet been adopted on a large scale and are not yet widely used. No research conducted by the platform owners, or by research institutes, has yet focused on the impact, or on the effects that are related to the initial objectives of the online platforms. There is a lack of evidence on the added value of these online care platforms, and what impact they have on aging, social cohesion, care coordination or management, and self-sufficiency of older adults. |  |
| **Multi-focal study** | | | | | | | | | |
| [21] | | 1. Patient satisfaction, patient empowerment, organisational factors and cost-effectiveness. 2. Social, cultural, and ethical aspects. | | None | | 1. Safety aspects were superficially examined; a more conscientious approach would be expected when trying out technical innovations in healthcare. 2. Study selection may have been biased. 2. Results were remarkably heterogeneous and could not be numerically aggregated. | | 1. Future studies should determine the extent to which human and financial resources are planned (and funded) to execute a comprehensive scientific evaluation of the use of telemedicine in Germany. 2. Patient satisfaction, patient empowerment, organisational or economic benefits should be considered when planning new technologies. 3. Social, cultural or ethical aspects, which are becoming more and more important, should also be significantly expanded and approached when developing telemedicine tools. |  |
| [29] | | N/A | | None | | 1. Use of only two scientific databases and two medical conference proceedings platforms. 2. Use of a limited set of telemedicine associated terms. 3 | | 1. There are few studies in this field. 2. The selected profile of represented disciplines favours certain specialties. |  |
| [31] | | Access to broadband, usability factors, and accessibility support for communication technologies. | | 1. Access and patient training. 2. Integrating patients and clinicians in the use of technology and virtual care within clinical workflows. 3. Participants’ willingness to use and adopt technology. 4. Access to broadband, usability factors and accessibility support for communication technologies. | | 1. Some relevant studies may have been missed. 2. There was high heterogeneity among included studies. 3. Studies that indirectly evaluated the use of remote shared decision-making systems were included. | | 1. The need to enhance cooperation between patients and clinicians in terms of research development. 2. The training of both patients and researchers is crucial for the success of any remote tool. 3. There is a constant challenge of integrating remote encounters into patients’ daily routines and clinicians’ agendas. 4. Regulatory features require appropriate attention from the scientific community and lawmakers. |  |
| [33] | |  | | The most commonly identified methodological strengths of the reviewed literature include (1) participatory design; (2) multi-centre, randomised controlled trials of adequate duration and sample size, usually preceded by a feasibility or pilot study; (3) interventions developed and/or performed by a multi-disciplinary palliative care team, addressing multiple aspects of palliative care; (4) use of validated ePROs measuring symptoms, QoL, and supportive care needs; and (5) evaluation of the eHealth system in terms of usability and user satisfaction. | | 1. Studies were methodologically diverse (including unfinished lines of research such as protocols and pilot/feasibility studies), meaning that the full impact of ePRO interventions on health outcomes could not be fully ascertained. | | The aim of future studies should be to assess the applicability and effectiveness of digital health interventions developed for patients with cancer in need of palliative care in its broader scope. Specifically, they should aim to facilitate or promote the delivery of palliative care from the point of diagnosis and throughout the continuum of the disease. |  |
| [34] | | Telemedicine implementation faced financial barriers. | | Clinical/professional benefits and financial benefits. Financial barriers could be overcome by healthcare organisation departments having clinician drivers or telemedicine champions. | | Incomplete and inconsistent data in the Telemedicinsk Landskort resulting in limited ability to compare projects, inability to determine the number of patients per project, and various other limitations. | | Federal-level authorities should mandate a common standard for interoperable telemedicine databases to provide critical information to organisations hoping to dialogue with other organisations, form knowledge-sharing networks for specific telemedicine apps, and avoid implementation pitfalls. It is therefore important to enforce strict regulations in order to ensure complete, accurate, and up-to-date information and optimal sharing of telemedicine knowledge across international boundaries. |  |
| [39] | | Some people voiced the negative aspects of less personal contact through telemedicine, as well as invasiveness. | |  | | 1. Quality limitations of included studies (small sample sizes, high dropout rates, lack of heterogeneity among quantitative studies, and short duration of studies). To limit the impact on reliability of results, data were combined in a sub-analysis and reported separately. 2. The random-effects model used in the meta-analyses had known limitations (overestimation of effect size and wider confidence intervals). 3. Only one researcher was responsible for the search strategy and decisions on the inclusion and exclusion of studies. To minimise selection bias, the search strategy was nevertheless checked and approved by two members of the supervisory team, and final studies for inclusion were agreed upon with one supervisor. 4. No language restriction was used when carrying out the search strategy but, despite that, all included studies in this review were in English. | | 1. Telehealth may offer reassurance to those living in the community with long-term conditions. 2. Cost-utility was calculated using QALYs in one study only. 3. More high-quality randomised controlled trials are needed, which are specific to a technological intervention and disease status for results to be clinically meaningful and truly reflective of usability and impact. |  |
| [40] | | 1. Older and lower socio-economic populations were less willing to use e-consultations. 2. Perceived level of seriousness and severity of health issue influenced the use of e-consultations, with patients being less willing to use e-consultation for more serious health issues. 3. Clinicians’ reluctance to use email with their patients because of increased workload. | | Convenience was the primary reported reason for patients choosing an e-consultation across multiple studies. Timeliness of response was important to patients using email and was associated with satisfaction. | | In a fast-moving field, it was impossible for reviews to always include the latest developments, and some of them may have been commercialised without there being publications on them. In addition, the authors faced the challenge of appraising whether recent studies carried out in outpatient clinics were relevant to primary care. In conducting the review, the authors also found some technology and infrastructure differences between the countries, including limitations in regard to using emails to communicate with patients. That may also have limited the reporting of results, especially if some studies could not be translated into English. | | For primary healthcare staff, e-consultation delivers challenges around time management, having the correct technological infrastructure, whether it offers a comparable standard of clinical quality, and whether it improves health outcomes. There is a lack of good quality evidence demonstrating positive patient outcomes from e-consultations because of the heterogeneity of evidence, which makes an accurate assessment of benefits difficult. In addition, there are limitations as to the longevity of follow-up data in trial material, again limiting the generalisability of any findings. |  |
| [41] | | Major barriers to the use of PRO include older age, lack of symptoms, tediousness of data entry, not thinking it is useful, being too busy, poor eyesight, language barriers, technical barriers and errors, and data security concerns. | | The need to design digital PRO interventions in collaboration with patients, for example, in participatory design studies. | | 1. Search strategy challenges due to the broad nature of the question. 2. Potential bias due to the exclusion criteria. 3. In most included studies, clinicians invited patients to participate in the intervention; the sample of patients may therefore have been biased before the recruitment process started and clinicians themselves may have been a significant barrier to the use of digital PROs. | | Some reasons given for non-participation prior to the start of an intervention may be related to fear or expectations. These can be addressed by providing patients with information and by screening for identified concerns. The digital solution is often not a stand-alone one, and interaction with the clinic and surrounding factors may play a significant part in a successful intervention. |  |
| [42] | | IT processes required major changes to speed up the introduction of new technologies into practice. No tariff existed for teleconsultations, which meant that managers were often unwilling or unable to justify diverting the cost of such services from increasingly stretched clinical budgets. The lack of integration of technology into routine practice, funding and hospital reimbursement, IT infrastructure, and integration into clinicians’ workflow. | | Teleconsultations appeared safe and effective in the right clinical situations. Where offered, it was likely that patients would be keen to engage, although teleconsultations should only be offered as an option to support traditional care models rather than replace them outright. Healthcare staff should be encouraged and supported in using teleconsultations to diversify their practice. Healthcare organisations need to consider developing a digital technology strategy and implementation groups to assist healthcare staff to integrate digitally enabled care into routine practice. The introduction of new technologies should be assessed after a set period with service evaluations, including feedback from key stakeholders. | | Scoping reviews are not intended to assess the quality of the literature included; therefore, the conclusions of this review were based on the existence of published research rather than its quality. | | It is necessary to integrate technology into usual practice and clinicians’ workflow, with the establishment of hospital financing and reimbursement systems. |  |
| [45] | | 1. The system was not completely reliable. 2. Technical problems. 3. Technology made life more difficult. 4. Low level of education. Knowledge was insufficient. Lack of appropriate training. 5. Teachers were too quick. 6. Difficulties with new systems. 7. Feeling that there was no need to use technology. 8. Expensive. 9. Assistance was needed when using it. 10. Functional limitations due to age. 11. Concerns about privacy due to the monitoring system’s technology. | | 1. Feeling safe with technological support. 2. Feeling of having power over technology. 3. Easy-to-use remote control unit. 4. Possibility for direct help, if needed. 5. An experienced older person taught other older people. 6. User-friendly web design. 7. Repeated practice provided skills and confidence. 8. High level of education. 9. Younger age. 10. More experience with technology. 11. Financial benefit. 12. Safety factor. 13. Communication with family and friends. 14. Technology gave a sense of security, contact with family, independence, helped to reach help. 15. Help from family about how to use the device. | | The results obtained correspond to experiences in Nordic countries. Scarce evidence relating to people aged 75 years and over on this topic. | | More research on this age group is needed. There should be a balance between the potential benefits and the effort required because it was found that older people were more open to new devices if the potential advantages of the new technology outweighed the effort involved in adopting a new strategy. Solutions should be designed by taking into consideration certain age-related physical and cognitive issues (motor disturbances, memory loss, etc.). |  |
| [51] | | 1. Difficulty in making clinical decisions. 2. Lack of motivation/support. 3. Changes to consultation type. 4. Attitudes towards future use of remote consultation. 5. Workload increased. 6. Concerns about patients’ privacy. 7. Monetary concerns. 8. Physicians missing appointments. 9. Changes in difficulty booking appointments and in waiting times. 10. Lack of confidence in, and access to, the technical skills/ technology required for remote consultations. 11. Loss of non-verbal communication and doctor-patient rapport. 12. Loss of physical and visual assessment of symptoms. 13. Face-to-face consultation required for complex issues. 14. Communication barrier due to language/hearing difficulties. 15. Insufficient consultation time. 16. Satisfaction level with remote consultation. 17. Consultation preferences. | | PCPs: Reimbursement for previously ‘free’ services; remote consultations enabled better monitoring of cases; increased appointment adherence; increased involvement by family members and insights into a patient’s home environment; patients felt empowered to discuss more personal issues via remote consultation; attitudes towards future use of remote consultation; workload decreased. Patients: Changes in difficulty booking appointments and in waiting times. Patients and PCPs: Convenience; reduced risk of COVID-19; satisfaction level with remote consultation; consultation preferences. | | Only two databases were searched. Only studies in English were included. No formal quality assessment was conducted, and the results were taken at face value. | | Further research is required in developing nations, where they tend to have different health systems to those available in developed countries. |  |
| [48] | | 1. Intervention studies carried out in a certain region could be applied, with some limitations, to another region. 2. There is still a limitation on conducting double-blinded studies in the field of telemedicine. | |  | | 1. High heterogeneity among included studies in terms of publication designs, performance assessment tools, and intervention and control groups. | | 1. The established endpoints were very heterogeneous. 2. Intervention performance tools as well as the high number of potential study protocols made the grouped and final assessments challenging. Researchers should therefore focus on standardising intervention protocols and effect evaluation documents. |  |
